# Diversity‐Oriented Synthesis and Antibiofilm Evaluation of Furan‐2‐Carboxamides

**DOI:** 10.1002/cmdc.202400879

**Published:** 2025-01-31

**Authors:** Ana C. Muñoz‐Estrada, Cesar E. Tovar‐Roman, Carlos D. García‐Mejía, Rodolfo García‐Contreras, Eduardo Hernández‐Vázquez

**Affiliations:** ^1^ Departamento de Química Orgánica Instituto de Química Universidad Nacional Autónoma de México (UNAM) CDMX México; ^2^ Departamento de Microbiología y Parasitología Facultad de Medicina Universidad Nacional Autónoma de México (UNAM) CDMX México

**Keywords:** Furan-2-carboxamides, antibiofilm, *Pseudomonas aeruginosa*, LasR

## Abstract

A diversity‐oriented collection of furan‐2‐carboxamides with antibiofilm activity against *P. aeruginosa* is reported. The design involved the bioisosteric replacement of the labile furanone ring by a furan‐2‐carboxamide moiety to explore its influence on biological activity. After evaluation, carbohydrazides and triazoles showed significant antibiofilm activity, and **4b** resulted in the most remarkable compound (58 % inhibition). Furthermore, treating *P. aeruginosa* with three active carboxamides reduced some virulence factors (pyocyanin and proteases), confirming the anti‐quorum sensing properties of the derivatives and suggesting LasR as a plausible target. Molecular docking proposed that carbohydrazides share a similar binding mode to related furanones inside LasR with an excellent docking score, while higher derivatives diminished *in silico* affinity.

## Graphical abstract

Navigating through the chemical space of antibiofilm agents! Furan‐2‐carboxamides inhibit the production of this extracellular shield in *Pseudomonas aeruginosa* by modulating the linkers and substituents of the central core. The discovery offers a solution to the metabolically unstable antibiofilm agents based on furanones, leading to the design of more potent derivatives.

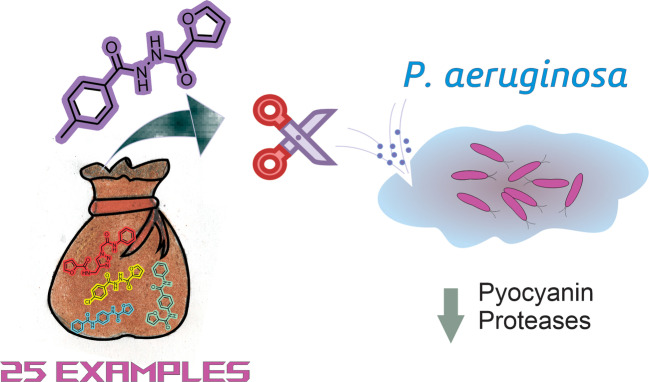

## Introduction

Biofilms are complex structures that allow pathogens to survive against harmful agents (chemical, physical, and even biological). In medicine, bacterial biofilm limits antimicrobial penetration and makes microorganisms hard to remove, thus leading to persistent infections.^[1]^ It is well known that biofilms are also responsible for colonizing medical disposables and equipment.^[2]^ Moreover, the intimate contact inside the bacterial microenvironment facilitates genetic material exchange (horizontal gene transfer),^[3]^ while increasing the rates of mutation,^[4]^ two conditions that may eventually promote drug resistance development and propagation. According to the above, there exists an urgency for discovering antibiofilm agents as an alternative for controlling multidrug‐resistant associated diseases since they caused more than 2.8 million infections and about 35,000 deaths in 2019.^[5]^ Antibiofilm approaches, as well as others focusing on controlling resistance factors, have gained huge attention due to the diminished selection pressure that excludes the possibility of drug resistance.[^6^, ^7^] Once weakened, antimicrobials and the immunologic system can control the infection.

**Figure 1:**
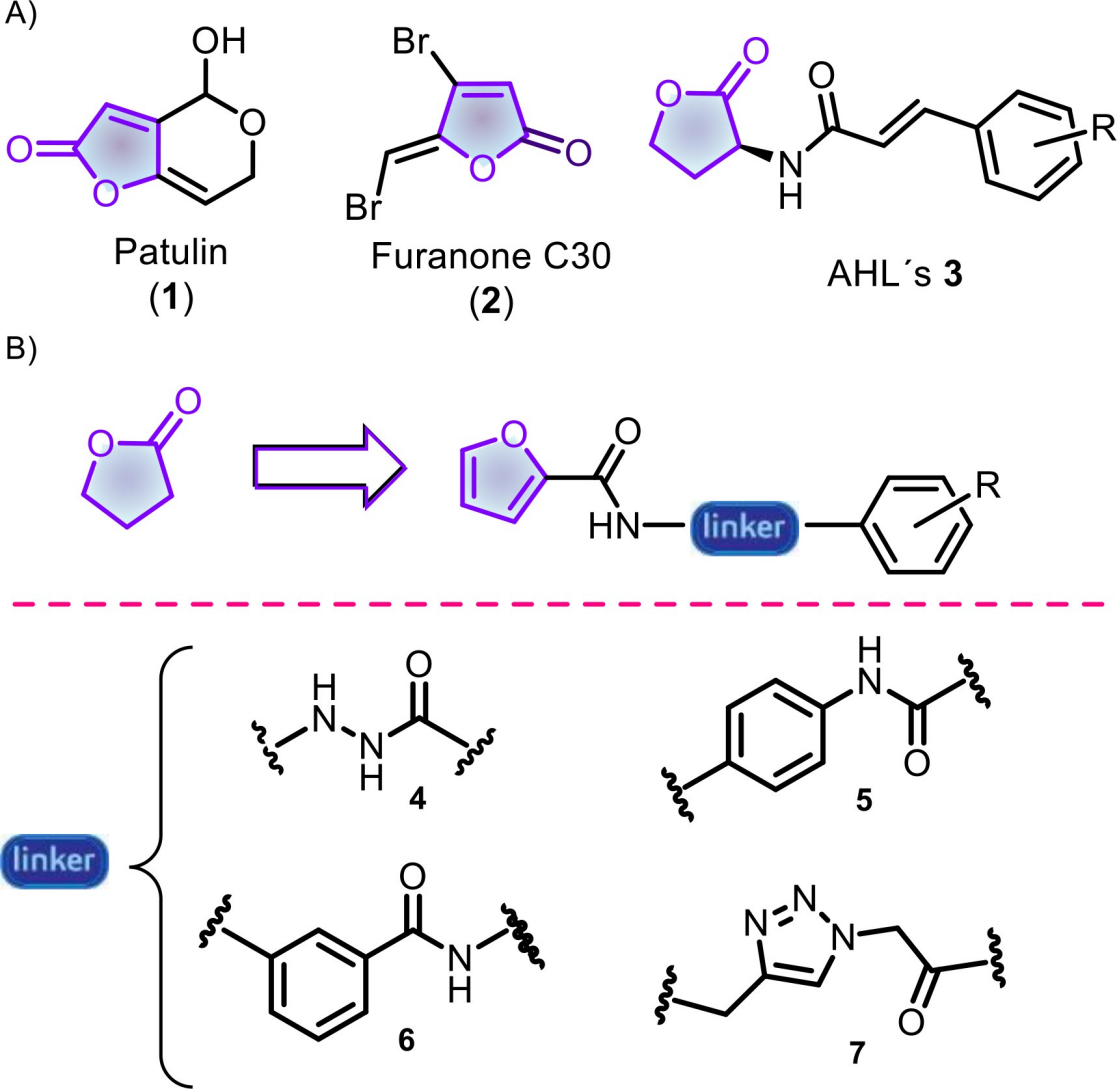
A) Relevant antibiofilm furanones; B) Furanone replacement by a furan‐2‐carboxamide led to four series of potential antibiofilm molecules.

Among the top‐listed pathogens, *Pseudomonas aeruginosa* is an opportunist that infects patients with cystic fibrosis, chronic obstructive pulmonary disease, or immunocompromised people.^[8]^ In addition, this Gram‐negative bacterium can mutate and owns an arsenal of resistant factors to avoid the actions of drugs, including biofilm‐mediated resistance.^[9]^ In *P. aeruginosa*, several quorum sensing systems regulate the biofilm cycle, being LasI/LasR the one with the highest hierarchy.^[10]^ Both natural and synthetic furanones inhibit LasR such as patulin (**1**),^[11]^ classical furanone C30 (**2**),^[12]^ or related analogs.^[13]^ Recently, we reported the synthesis and antibiofilm activity of a series of *N*‐cinnamoylhomoserine lactones capable of inhibiting biofilm and quenching quorum sensing in ESKAPE pathogens, including *P. aeruginosa* (Figure 1).^[14]^ However, lactone rings tend to be prone in aqueous media, and, in most cases, the compound loses its activity,^[15]^ thus limiting its future applications. Therefore, we envisioned the bioisosteric replacement of lactone by a furane‐2‐carboxamide to construct four series of molecules in a diversity‐oriented fashion, maintaining the carbonyl group necessary to interact with Trp60 or Tyr56 into the LasR binding cavity (interactions found in other antagonists). In addition, we preserved the aromatic ring found in **3** while replacing the alkene with different linkers: *N‐*acylcarbohydrazide (**4**), 1,4‐diaminobenzene (**5**), 3‐aminobenzoic acid (**6**), and a 1,2,3–1*H*‐triazole heterocycle (**7**).

## Results and Discussion

### Chemistry

**Scheme 1:**
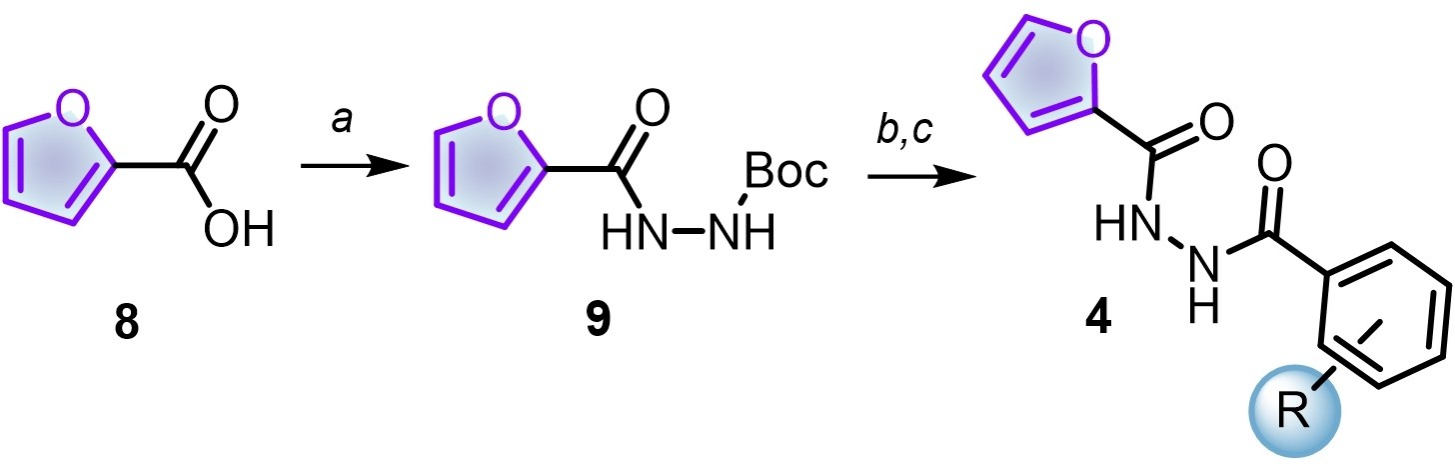
Synthesis of *N*‐acylcarbohydrazides 4. Reagents and conditions: a) CDI, THF, 45 °C, 1 h, then BocNHNH_2_; b) TFA, DCM, r.t., 3 h; c) previously activated benzoic acid, THF, 45 °C.

The approach considered a synthetic sequence involving the amide formation of furoic acid with four different linkers and then coupling with a phenyl ring bearing electron‐withdrawing, electron‐donating, and steric substituents. We started the journey by preparing the less complex series **4**, consisting of a *N*‐acylcarbohydrazide (Scheme 1). Accordingly, we used CDI (1,1'‐carbonyldiimidazole) to activate furan‐2‐carboxylic acid (**8**) and then treated it with *t*‐butylcarbazate to isolate the corresponding carbohydrazide **9** in good yield (90 %). The acidic cleavage of Boc in **9** with trifuoroacetic acid (TFA) followed by coupling with previously activated benzoic acids (again, CDI served as coupling reagent), afforded the series **4** in good yields (24–68 %).

**Scheme 2:**
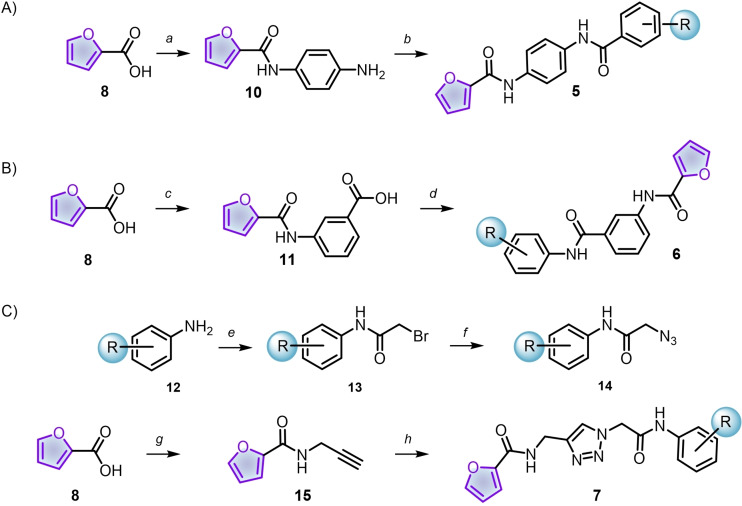
Syntheses of series **5** (A), **6** (B) and **7** (C). Reagents and conditions: a) CDI, THF, 45 °C, 2 h, then 1,4‐diaminobenzene, 45 °C, 18 h; b) previously activated benzoic acids, THF, 45 °C, 20 h; c) CDI, THF, 45 °C, 2 h, then 3‐aminobenzoic acid, 45 °C, 20 h; d) CDI, THF, 45 °C, 2 h, then, corresponding substituted aniline; e) bromoacetyl chloride, DCM, 0 °C, 2 h; f) NaN_3_, DMSO, 80 °C, 1–2 h; g) DCC, 4‐DMAP, propargylamine, DCM, 0 °C to r.t., 24 h; h) 14, CuSO_4_ ⋅ 5H_2_O, sodium ascorbate, DMSO, r.t., 10 h.

For the synthesis of diamines **5** (Scheme 2), the activated carboxylic acid **8** was coupled with 1,4‐diaminobenzene under diluted conditions and with a dropwise addition of the electrophile. Thus, the prepared amide **10** reacted with previously activated benzoic acids, leading to the diamide **5**. In the case of compounds **6**, amide **11** was prepared similarly as shown before but using 3‐aminobenzoic acid; the built benzoic acid **11** was then activated with CDI and bonded with substituted anilines to afford the desired diamides.

Finally, Scheme 2C shows the preparation of triazoles **7**. In this case, we employed a convergent tactic in which a triazole ring joined azides **14** and *N*‐(prop‐2‐yn‐1‐yl)furan‐2‐carboxamide (**15**). A straightforward protocol constructed the amidoazides **14** starting from anilines **12**. First, an acylation with bromoacetyl chloride gave access to the amides **13** that were submitted to a bimolecular substitution (S_N_2) with sodium azide. Having in hand azides **14** and alkyne **15**, classical conditions of copper(I)‐catalyzed azide‐alkyne cycloaddition (CuAAC) led to the construction of triazoles **7** (Cu^1+^ was formed *in situ* by the reduction of CuSO_4_ with sodium ascorbate).

Table 1 resumes the structures and yields of the final products. As mentioned above, we explore the effects of substituents attached to the four series: electron‐donating (methyl and methoxy), electron‐withdrawing (chlorine), and bulky (*t*‐butyl) groups. In the case of *N‐*acylcarbohydrazides (series **4**), the simplicity of the synthetic methodology allowed the preparation of more analogs.

**Table 1:**
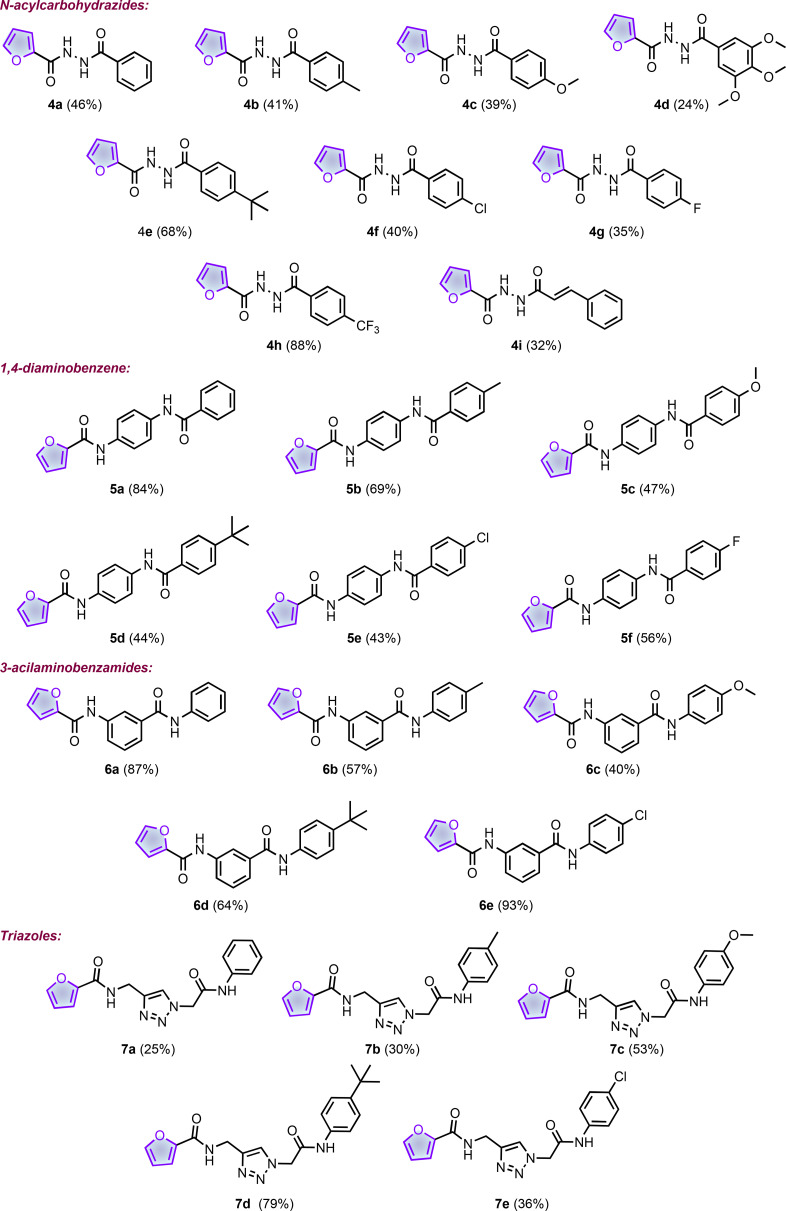
Series of furan‐2‐carboxamides. The yields correspond to the last step in the synthesis.

### Preliminary Antibiofilm Screening

Once the derivatives were completely characterized (See supporting information), we determined if compounds could reduce biofilm formation in *Pseudomonas aeruginosa* PA01. The design conserved the carbonyl moiety shown in **2** and series **3**, while we added linkers with different sizes, electronic characteristics, and lengths to evaluate the influence of the antibiofilm activity. The carbohydrazide linker owns two acidic hydrogens that could construct hydrogen bonds inside the LasR cavity, while the aromatic ring at series **5** and **6** could not only enhance the length of the molecule (mimicking the aliphatic chain of LasR agonists) but may also interact with surrounding aromatic amino acids. Finally, the triazole moiety is a commonly used linker due to its stability, the possibility of acting as an amide bioisostere, and its nitrogen atoms that might assemble hydrogen bonds with the receptor.^[16]^ Those characteristics are responsible for the addition of triazole in uncountable bioactive substances such as anti‐SARs‐CoV‐2,^[17]^ antibacterial,^[18]^ and even anti‐cancer.^[19]^

**Figure 2:**
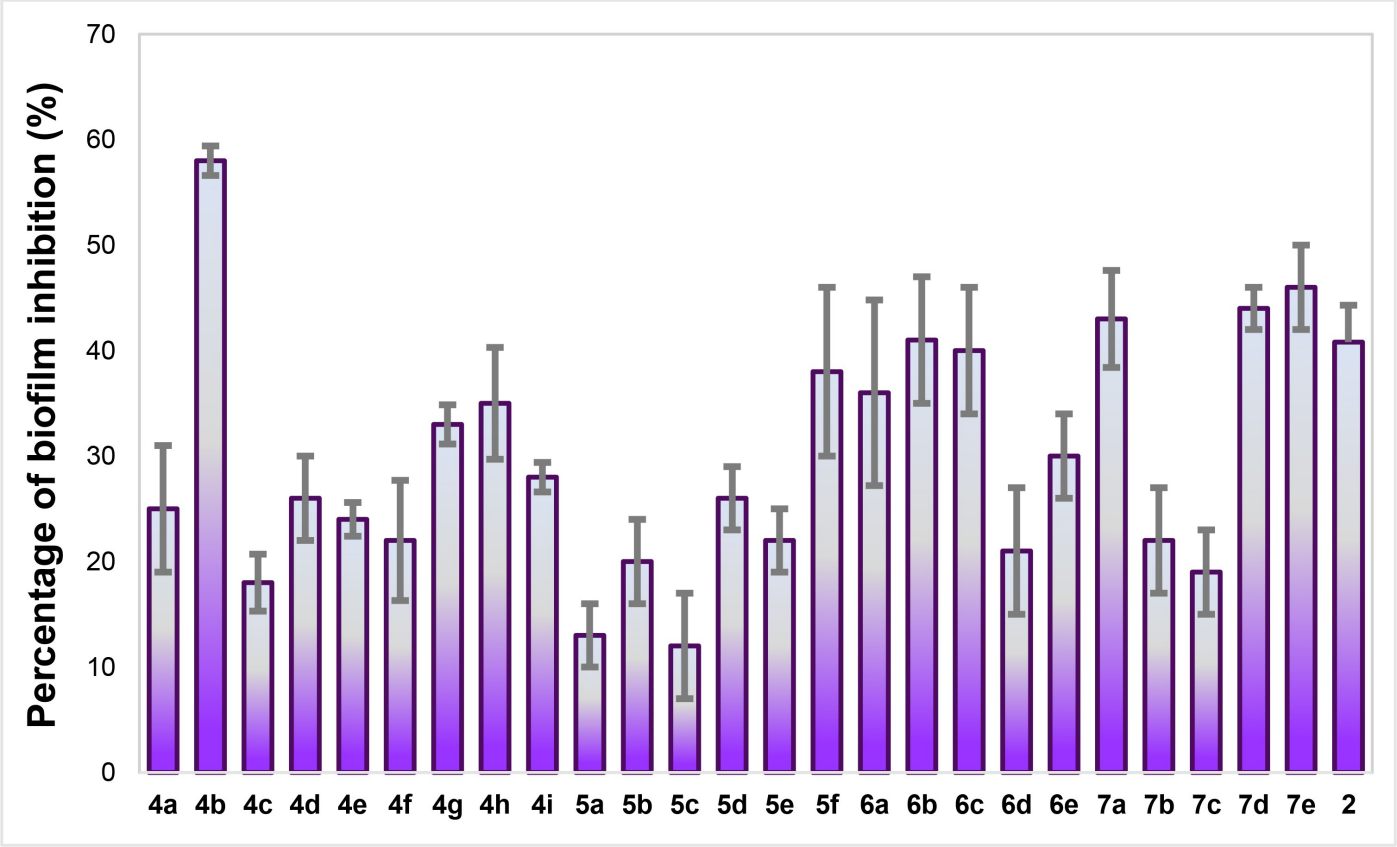
Percentage of biofilm inhibition at 50 μM of the four series. The graphic represents the mean±SEM (three different experiments).

We considered the crystal violet method, a trianiline‐based pigment that binds to the extracellular matrix of biofilm, becoming a frequent method for evaluating anti‐biofilm agents.^[20]^ The percentage of inhibition at 50 μM is resumed in Figure 2. To our delight, none of the furane‐2‐carboxamides inhibited the growth of the pathogen, therefore, the observed effect relies on antibiofilm capacity. Accordingly, all the furan‐2‐carboxamides exhibited a reduction of biofilm higher than 10 % (some of them surpassing the inhibition of furanone **2**), but the *p*‐phenylenediamines **5a**–**e** displayed the lowest inhibition (excluding for the Fluorine substituted **5f**, with 38 % of inhibition). The other series had irregular outcomes; however, the aminobenzoic acid‐derived (series **6**) and triazole analogs (series **7**) were the most interesting compounds with inhibition higher than 30 % (in most of the cases). Series **5** and **6** are closely related but show distinct inhibition; a plausible explanation may be related to the conformation adopted in the diamine derivatives, interfering with the correct fitness of the molecules inside LasR. On the other hand, triazoles (series **7**) became a surprise since they showed a significant inhibition despite being the largest furan‐2‐carboxamides of the four series. Previously, we found bulky groups reduce antibiofilm activity, probably due to steric hindrance into the LasR active site.^[14]^ Thus, the aliphatic chains at the 1 and 4 positions of the 1,2,3‐triazol core facilitate a favorable conformation in the ligand‐receptor complex, although another mechanism is not discarded.

It is worth noting that the most active substances bore a methyl chain (**4b**=58 %; **6b**=41 %), halogen atoms (**4g**=33 %; **4h**=35 %; **7e**=46 %) or were unsubstituted (**6a**=36 %; **7a**=43 %), demonstrating that the substitutions at the phenyl ring are a crucial feature to inhibit biofilm. We want to highlight the activity of carbohydrazide **4b**, which reduced 58 % of *Pseudomonas aeruginosa* biofilm and became the most active furan derivative. Attempts to determine the IC_50_ were futile, mainly attributed to the precipitation of compounds at higher concentrations; for example, **4b** showed a concentration‐response curve at 0.5, 5, 25, and 50 μM, but the activity dropped at higher concentrations (See Figure S1 in Supporting Information). Only **4a** reached 50 % of biofilm inhibition near 50 μM. Increasing concentration of carboxamides **6b**, **7a**, or **7e** contributed to reducing biofilm, but with lower efficacy than carbohydrazide 6a. Given those results, future works will focus on increasing the solubility of these potential antibiofilm agents. The calculated IC_50_ of **2** resulted in 103.7±4.8 μM (Figure S2) but had only 40 % inhibition at 50 μM; in the case of **4b**, it reached 50 % reduction at the same concentration, thus proving the potential applicability of the furan‐2‐carboxamides.

After pointing out the antibiofilm activity of the furan‐2‐carboxamides, we became interested in determining if some analogs can diminish QS‐related virulence factors in *P. aeruginosa*. For this purpose, the most active derivatives (carbohydrazide **4b** and triazoles **7d** and **7e**) underwent the study. An arsenal of virulence factors (such as pyocyanin, pyoverdine, and several proteases) facilitates disease in *P. aeruginosa*. For example, pyocyanin, a phenazine‐based pigment, plays a key role during infections by affecting the redox cycle of the host through the overproduction of oxygen‐reactive species, damaging tissues and organs.[^8^, ^21^] Analogously, *P. aeruginosa* secretes a myriad of lytic enzymes (proteases, elastases, lipases, esterases) that help during invasion and alter the cell signaling pathways.^[22]^

Table 2 resumes the inhibition of quorum sensing‐associated virulence factors such as pyocyanin^[23]^ and proteases.^[24]^ Carboxamides **4b** and **7e** reduced the proteases after incubation (18 and 16 %, respectively), while triazole **7d** had a minimum effect (6 %). Notwithstanding, we found better outcomes regarding the dye pyocyanin: the tested carboxamides lessened its production above 25 %, and **7d** resulted in the most active compound with a 33 % inhibition. Since those factors are regulated by quorum sensing, along with the considerations assumed during the design of the molecules, we conclude that the series probably acts through LasR antagonism (*vide infra*). Despite **4b** and **7e** inhibiting both virulence factors, their activity does not compare to the observed for **2** (inhibition of proteases and pyocyanin of 87 and 76 %, respectively). Moreover, the accessibility of the synthetic protocol allows the preparation of more derivatives that could eventually surpass C‐30 activity.

**Table 2:** Percentage of proteases and pyocyanin inhibition for some furan‐2‐carboxamides.

Compound	Proteases inhibition (%)	Pyocyanin inhibition (%)
**4 b**	18	26
**7 d**	6	33
**7 e**	16	30
**2**	87	76

**Figure 3:**
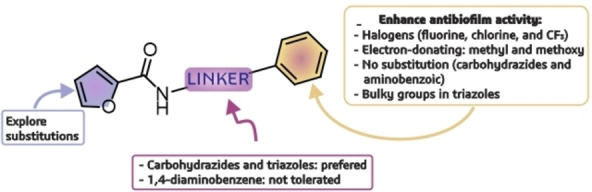
Main structural features for antibiofilm capacity of furan‐2‐carboxamides. Substitutions at the furan ring will be explored in future works.

Even though low solubility avoided the calculation of IC_50_, we can draw some assumptions about structural features that led to antibiofilm activity in furan‐2‐carboxamides (Figure 3). First, the best linker resulted in the triazole ring or the *N‐*acylcarbohydrazides (examples: **4d**, **7d**, and **7e**); some carboxamides with 3‐aminobenzoyl also displayed good activity, but the 1,4‐diaminobezene dropped the activity. In the case of the nature of the functional groups at the phenyl ring, halogens led to a better inhibition of biofilm. Electron‐donating groups like methyl or methoxy revealed similar findings. Bulky alkyl chains reduced the biological activity except for the triazole series **7**, in which the most active substance had a *t*‐butyl. Finally, no substitutions favored the inhibition of carbohydrazides **4**, and aminobenzoic derivatives **6**. For future works, exploring other functional groups at the phenyl moiety of triazoles and carbohydrazides may be an excellent starting point for modifications. Besides, we want to investigate the effects of attaching groups at the furan ring.

### Molecular Docking

We performed a molecular docking study to propose a plausible mechanism of action for the organic substances shown herein. Glide docking suit^[25]^ and LasR (PDB 2UV0) served as software and template, respectively. We selected this template due to its excellent resolution (1.8 Å) along with the co‐crystallization with *N*‐3‐oxo‐dodecanoyl‐L‐homoserine lactone (C12), one of the main autoinducers in *P. aeruginosa* QS system;^[26]^ the furanone was also docked (extra‐precision protocol) into LasR, adopting a similar binding pose –despite the flexible dodecyl chain pendant to the furanone ring‐ as the co‐crystallized ligand (RMSD value of 1.33 Å), thus validating the employed protocol (Figure S2 in Supporting Information).

**Figure 4:**
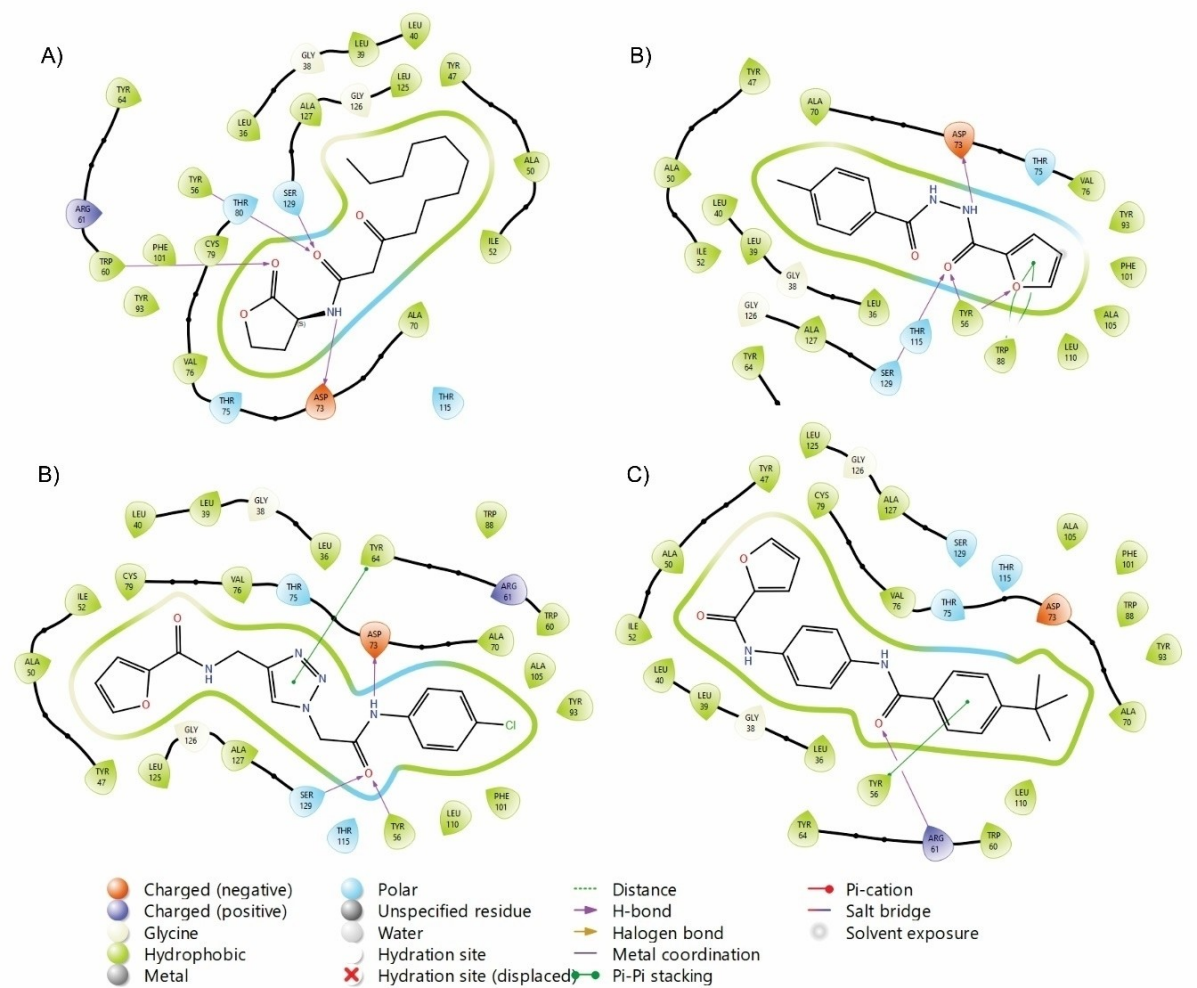
Ligand interaction plot of the most active furan‐2‐carboxamides inside LasR (PDB: 2UV0): A) C12; B)4b; C) 7d; D) 7e. Hydrogen bonds are depicted as purple arrows.

All the furan‐containing carboxamides were satisfactorily docked inside LasR, with scores ranging from −10.05 to −6.20 (Table 3), occupying the same cavity as the autoinducer C12 (Figure S3 in supporting information). In most cases, the furan heterocyclic ring fills the same region as the furanone of C12, while the phenyl ring binds mimetics the alkyl chain. We want to highlight that the smallest carboxamides (*i. e*. carbohydrazides) possess the best docking score among the series (−9.00 to −10.05), surpassing the predicted affinity of furanone‐containing autoinducer (−8.08). Since the site occupied by the autoinducer is small, furan‐derived carbohydrazides fit better than the other series. In addition, we previously found that *N*‐acylhomoserine lactones bind in a similar fashion to C12: four hydrogen bonds with Tyr56, Trp60, Asp73, and Ser129 stabilize the ligand‐receptor complex.^[14]^ Remarkably, carbohydrazides **4a**–**i** not only conserve three of the mentioned interactions (the lack of the carbonyl group deletes the hydrogen bond with Trp60) but also interact with Trp88 due to the presence of the aromatic furan. The sum of all interactions contributes to the excellent docking score (Figure 4). The other series had fluctuant results, but two features diminished affinity: *t‐*butyl substituent is not tolerated (examples **5d**, **6g**, and **7d**) and the triazole‐base analogs (**7a**–**e**) showed the lowest affinity. Since series **7** showed significant activity on the preliminary screening, (and had an opposite binding pose, see Figure 4B and C) they could probably act through a different mechanism of action like inhibition of RhlR or the quinolone‐based system.[^27^, ^28^]

**Table 3:** Results after molecular docking using LasR as the target. Relevant interactions with amino acids are shown.

Compound	Docking score	Hydrogen bond formation				π‐π Stacking (Trp88)	Other interactions
		Tyr56	Trp60	Ser129	Asp73		(hydrogen bond)
**4a**	−9.85	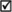	–	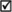	–	–	–
**4b**	−9.14	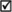	–	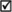	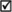	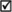	–
**4c**	−9.00	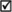	–	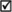	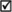	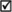	–
**4d**	−9.54	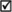	–	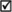	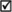	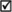	–
**4e**	−9.95	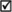	–	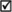	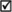	–	–
**4f**	−9.30	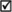	–	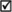	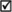	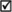	–
**4g**	−9.52	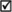	–	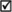	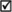	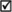	–
**4h**	−10.05	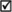	–	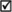	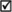	–	–
**4i**	−9.73	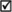	–	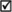	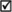	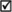	Thr75
**5a**	−8.19	–	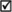	–	–	–	Tyr47
**5b**	−6.84	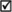	–	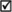	–	–	–
**5c**	−7.64	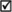	–	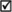	–	–	–
**5d**	−4.897	–	–	–	–	–	Arg61
**5e**	−8.00	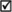	–	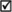	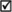	–	–
**5f**	−6.92	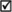	–	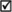	–	–	–
**6a**	−8.66	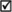	–	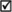	–	–	Phe101
**6b**	−6.96	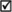		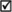	–	–	Tyr47
**6c**	−7.30	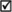		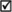		–	Tyr47 and 64
**6d**	−6.20	–	–	–	–	–	–
**6e**	−8.38	–	–	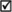	–	–	Tyr47
**7a**	−9.37	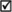	–	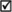	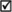	–	Phe101
**7b**	−7.13	–	–	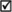	–	–	Phe101, Arg61
**7c**	−7.01	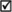	–	–	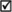	–	–
**7d**	−6.90	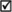	–	–	–	–	Tyr64
**7e**	−7.99	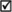	–	–	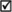	–	Phe101
**C12**	−8.08	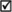	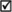	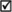	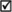	–	–

## Conclusions

A diversity‐oriented synthesis was developed to explore the potential antibiofilm capacity of furan‐2‐carboxamides, designed as bioisosteric replacements of the labile furanone ring. All compounds showed a biofilm reduction in *P. aeruginosa* cultures, but carbohydrazides (series **4**) and triazoles (series **7**) were the most remarkable groups. Carbohydrazide **4b** became the most active derivative, reaching 58 % of biofilm reduction. Furthermore, **4b** and triazoles **7d** and **7e** showed a reduction of quorum sensing‐regulated virulence factors (proteases and pyocyanin). With this evidence, and considering the design, we proposed LasR as a plausible target for the antibiofilm activity; accordingly, molecular docking confirmed this QS‐related receptor as a target for the compounds, particularly series **4**, which had the best glide scores and shared similar hydrogen bond interaction than C12 and *N*‐acylfuranones. In the case of triazoles **7**, another possible mechanism could explain the observed *in vitro* activity.

We ensure that anti‐virulence compounds, especially those focused on biofilm elimination/prevention, are reliable strategies for the control of multi‐resistant ESKAPE bacteria. In this sense, our work points out the applicability of replacing furanone with furane‐2‐carboxamide for preparing new antibiofilm derivatives. We want to highlight the carbohydrazides **4a**–**I** as novel LasR antagonists.

## Experimental Section

### General Considerations

Reagents and solvents were acquired from Merck and Química Rique. NMR spectra were recorded with a Jeol Eclipse (300 MHZ) or on a Bruker Avance III 400 MHz spectrometer using CDCl_3_ and DMSO‐*d6*. Mass spectra (MS) and High‐resolution mass spectra (HRMS) were measured using a Jeol JMS‐T100LC The AccuTOF mass spectrometer with ionization technique DART (Direct Analysis in Real Time). Infrared (IR) spectra were collected with a Thermo Fisher Scientific model Nicolet iS50 FT‐IR spectrometer using the ATR (Attenuated Total Reflection) technique. Plates were read on a BioTek Cytation™ 5 Cell Imaging Multi‐Mode Reader. Compound characterization data (including full ^1^H, ^13^C‐NMR, and HRMS) are detailed in the supporting file. Melting points were determined with a Fisher‐Johnes apparatus and are uncorrected.

### Synthesis of *Tert*‐Butyl 2‐(Furan‐2‐Carbonyl)hydrazine‐1‐Carboxylate (9)

In a round‐bottom flask, furane‐2‐carboxylic acid (2.68 mmol) and 1,1'‐carbonildiimidazole (CDI, 2.94 mmol) were added and dissolved in THF (6 mL). The mixture was stirred for 2 h at 45 °C. After the consumption of the starting material, *t*‐butylcarbazate (2.94 mmol) was added and allowed to react for 18–20 h. The carbohydrazide was obtained as a beige solid in 90 % yield. ^**1**^ **H‐NMR** (CDCl_3_, 300 MHz) δ: 8.27 (br s, 1H), 7.44 (d, *J*=1.56 Hz, 1H), 7.18 (d, *J=*3.6 Hz, 1H), 6.75 (br s, 1H), 6.48 (dd, *J*=3.6 and 1.6 Hz, 1H), 1.47 (s, 9H); ^**13**^ **C‐NMR** (CDCl_3,_ 75.5 MHz) δ: 157.9, 155.6, 146.2, 144.9, 116.1, 112.2, 82.1, 28.2; **MS** (DART+) m/z [M+H]^+^: 227 m/z; **HRMS** *m/z* calcd for ^12^C_10_^1^H_15_^14^N_2_^16^O_4_ [M+H]^+^, 227.10318; found 227.10309.

### General Synthesis of Carbohydrazides 4a–i

Carbohydrazide **9** (0.2 mmol) was dissolved in DCM (0.8 mL), and trifluoroacetic acid (TFA, 1.11 mmol) was added dropwise. The mixture was stirred for 3–4 h at r.t. After completion of the reaction, the solvent and excess of TFA were distilled under vacuum and dried under vacuum for 12 h. The salt was used without purification. Afterward, the corresponding benzoic acid (0.22 mmol) and CDI (0.265 mmol) were added to a round‐bottom flask and dissolved in THF (0.5 mL). The mixture was heated at 45 °C for 2 h and then, a solution of the deprotected carbohydrazide (0.22 mmol) and DIPEA (0.33 mmol) in THF (1 mL) was added. The heating was maintained for 20 h. Finally, the solvent was removed, redissolved in AcOEt, and consecutively washed with HCl (10 %) and NaHCO_3_ (10 %). The product was purified by flash column chromatography.

#### N′‐Benzoylfuran‐2‐Carbohydrazide (4a)

Purified by flash column chromatography (Hex‐AcOEt 7 : 3) and obtained as a white solid in 46 % yield, mp 220–222 °C. ^**1**^ **H‐NMR** (DMSO‐*d6*, 400 MHz) δ: 10.45 (s, 1H, NH), 10.39 (s, 1H, NH), 7.94–7.89 (m, 3H), 7.62–7.58 (m, 1H), 7.55–7.50 (m, 2H), 7.26 (dd, *J*=3.4 and 0.8 Hz, 1H), 6.68 (dd, *J*=3.4 and 2.0 Hz, 1H); ^**13**^ **C‐NMR** (100 MHz, DMSO‐*d6*) δ: 165.8, 157.4, 146.3, 145.8, 132.4, 131.9, 128.5, 127.5, 114.6, 111.9; **MS** (DART+) m/z [M+H]^+^: 231 m/z; **HRMS** *m/z* calcd for ^12^C_12_^1^H_11_^14^N_2_^16^O_3_ [M+H]^+^, 231.07697; found 231.07689; **IR** (ATR, cm^−1^): 3151, 3052, 3008, 2930, 1668, 1630, 1526, 1290, 592.

#### N′‐(4‐Methylbenzoyl)furan‐2‐Carbohydrazide (4b)

Purified by flash column chromatography (Hex‐AcOEt 6 : 4) and obtained as a white solid in 41 % yield, mp 209–211 °C. ^**1**^ **H‐NMR** (DMSO‐*d6*, 400 MHz) δ: 10.37 (s, 1H, NH), 10.35 (s, 1H, NH), 7.91 (dd, *J*=1.2 and 0.6 Hz, 1H), 7.83–7.80 (AA'BB', 2H), 7.33–7.31 (AA'BB', 2H), 7.26 (dd, *J*=2.7 and 0.6 Hz, 1H), 6.68 (dd, *J*=2.7 and 1.2 Hz, 1H), 2.37 (s, 3H); ^**13**^ **C‐NMR** (100.6 MHz, DMSO‐*d6*) δ: 165.7, 157.4, 146.3, 145.8, 141.9, 129.7, 129.1, 127.5, 114.6, 111.9, 21.0; **MS** (DART+) m/z [M+H]^+^: 245 m/z; **HRMS** *m/z* calcd for ^12^C_13_^1^H_13_^14^N_2_^16^O_3_ [M+H]^+^, 245.09262; found 245.09295; **IR** (ATR, cm^−1^): 3172, 2957, 2921, 2851, 1673, 1630, 1504, 1282, 742.

#### N′‐(4‐Methoxybenzoyl)furan‐2‐Carbohydrazide (4c)

Purified by flash column chromatography (Hex‐AcOEt 6 : 4) and obtained as a white solid in 39 % yield, mp 197–198 °C. ^**1**^ **H‐NMR** (DMSO‐*d6*, 300 MHz) δ: 10.36 (s, 1H, NH), 10.31 (s, 1H, NH), 7.90–7.85 (m, 3H), 7.24 (d, *J*=3.6 Hz, 1H), 7.05–7.02 (AA'BB', 2H), 6.67(dd, *J*=3.6 and 1.8 Hz, 1H), 3.81 (s, 3H); ^**13**^ **C‐NMR** (76 MHz, DMSO‐*d6*) δ: 165.9, 162.4, 157.9, 146.5, 146.1, 131.7, 129.7, 115.0, 114.1, 112.3, 55.7; **MS** (DART+) m/z [M+H]^+^: 261 m/z; **HRMS** *m/z* calcd for ^12^C_13_^1^H_13_^14^N_2_^16^O_4_ [M+H]^+^, 261.08753; found 261.08699; **IR** (ATR, cm^−1^): 3176, 3006, 2924, 2851, 1632, 1605, 1502, 1251, 1186, 593.

#### N′‐(3,4,5‐Trimethoxybenzoyl)furan‐2‐Carbohydrazide (4d)

Purified by flash column chromatography (Hex‐AcOEt 3 : 7) and obtained as a beige solid in 24 % yield, mp 199–200 °C. ^**1**^ **H‐NMR** (DMSO‐*d6*, 400 MHz) δ: 10.42 (s, 2H, NH), 7.89 (dd, *J*=1.6 and 0.8 Hz, 1H), 7.26 (dd, *J*=3.6 and 0.8 Hz, 1H), 7.24 (s, 2H), 6.68 (dd, *J*=3.6 and 1.6 Hz, 1H), 3.83 (s, 6H), 3.72 (s, 3H); ^**13**^ **C‐NMR** (100.6 MHz, DMSO‐*d6*) δ: 165.5, 157.7, 152.9, 146.4, 146.0, 140.7, 127.6, 115.0, 112.2, 105.2, 60.4, 56.2; **MS** (DART+) m/z [M+H]^+^: 320 m/z; **HRMS** *m/z* calcd for ^12^C_15_^1^H_17_^14^N_2_^16^O_6_ [M+H]^+^, 321.10866; found 321.10865; **IR** (ATR, cm^−1^): 3247, 3002, 2924, 2839, 1644, 1586, 1494, 1083, 756.

#### N′‐(4‐(Tert‐Butyl)benzoyl)furan‐2‐Carbohydrazide (4e)

Purified by flash column chromatography (Hex‐AcOEt 7 : 3) and obtained as an off‐white solid in 68 % yield, mp 253–255 °C. ^**1**^ **H‐NMR** (DMSO‐*d6*, 300 MHz) δ: 10.39 (s, 2H, NH×2), 7.93–7.86 (AA'BB', 2H), 7.83 (d, *J*=1.8 Hz, 1H), 7.58–7.51 (AA'BB', 2H), 7.27 (br s, 1H), 6.68 (dd, *J*=3.6 and 1.8 Hz, 1H), 1.30 (s, 9H); ^**13**^ **C‐NMR** (76 MHz, DMSO‐*d6*) δ: 165.8, 157.5, 154.9, 146.4, 145.9, 129.7, 127.4, 125.4, 114.6, 112.0, 34.8, 31.0; **MS** (DART+) m/z [M +H]^+^: 287 m/z; **HRMS** *m/z* calcd for ^12^C_16_^1^H_19_^14^N_2_^16^O_3_ [M+H]^+^, 287.13957; found 287.13972; **IR** (ATR, cm^−1^): 3179, 3058, 2959, 2927, 1678, 1638, 1301, 1025, 745.

#### N′‐(4‐Chlorobenzoyl)furan‐2‐Carbohydrazide (4f)

Purified by flash column chromatography (Hex‐AcOEt 1 : 1) and obtained as a yellowish solid in 40 % yield, mp 187–189 °C. ^**1**^ **H‐NMR** (DMSO‐*d6*, 300 MHz) δ: 10.56 (s, 1H, NH), 10.43 (s, 1H, NH), 7.94–7.91 (m, 3H), 7.62–7.58 (AA'BB', 2H), 7.26 (dd, *J*=3.6 Hz, 1H), 6.68 (dd, *J*=3.6 and 1.8 Hz, 1H); ^**13**^ **C‐NMR** (75.5 MHz, DMSO‐*d6*) δ: 164.9, 157.4, 146.2, 145.9, 136.8, 131.2, 129.4, 128.7, 114.7, 112.0; **MS** (DART+) m/z [M+H]^+^: 265 m/z; **HRMS** *m/z* calcd for ^12^C_12_^1^H_10_^35^Cl_1_^14^N_2_^16^O_3_ [M+H]^+^, 265.03799; found 265.03800; **IR** (ATR, cm^−1^): 3150, 2960, 2924, 1633, 1590, 1276, 741, 593.

#### N′‐(4‐Fluorobenzoyl)furan‐2‐Carbohydrazide (4g)

Purified by flash column chromatography (Hex‐AcOEt 55 : 45) and obtained as a brown solid in 53 % yield, mp 189–190 °C. ^**1**^ **H‐NMR** (DMSO‐*d6*, 300 MHz) δ: 10.51 (s, 1H, NH), 10.48 (s, 1H, NH), 7.93 (dd, *J*=8.6 and 5.2 Hz, 2H), 7.84 (br s, 1H), 7.34–7.30 (m, 2H), 7.24 (d, *J*=3.6 Hz, 1H), 6.64 (br s, 1H); ^**13**^ **C‐NMR** (100.6 MHz, DMSO‐*d6*) δ: 172.1, 166.0, 164.9 (d, *J*=250.2 Hz), 158.3, 146.5, 130.8 (d, *J*=9.3 Hz), 129.3 (d, *J*=2.3 Hz), 116.3 (d, *J*=21.9 Hz), 115.8, 112.7; **MS** (DART+) m/z [M+H]^+^: 249 m/z; **HRMS** *m/z* calcd for ^12^C_12_^1^H_10_^19^F_1_^14^N_2_^16^O_3_ [M+H]^+^, 249.06755; found 249.06837; **IR** (ATR, cm^−1^): 3155, 3013, 1631, 1602, 1500, 1282, 846.

#### N′‐(4‐(Trifluoromethyl)benzoyl)furan‐2‐Carbohydrazide (4h)

Purified by flash column chromatography (Hex‐AcOEt 55 : 45) and obtained as a beige solid in 88 % yield, mp 190–191 °C. ^**1**^ **H‐NMR** (DMSO‐*d6*, 400 MHz) δ: 10.71 (s, 1H, NH), 10.50 (s, 1H, NH), 8.11–8.09 (AA'BB', 2H), 7.93–7.91 (m, 3H), 7.28 (dd, *J*=3.4 and 0.8 Hz, 1H), 6.69 (d, *J*=3.4 and 1.6 Hz, 1H); ^**13**^ **C‐NMR** (100.6 MHz, DMSO‐*d6*) δ: 164.8, 157.3, 146.2, 145.9, 136.3, 131.8 (q, *J*=31.6 Hz), 128.5, 125.6 (q, *J*=3.8 Hz), 123.9 (q, *J*=270.9 Hz), 114.8, 112.0; ^**19**^ **F‐NMR** (376.2 MHz, DMSO‐*d6*) δ: −61.4; **MS** (DART+) m/z [M+H]^+^: 299 m/z; **HRMS** *m/z* calcd for ^12^C_13_^1^H_10_^19^F_3_^14^N_2_^16^O_3_ [M+H]^+^, 299.06435; found 299.06396; **IR** (ATR, cm^−1^): 3210, 3026, 2925, 2850, 1682, 1644, 1372, 1139, 754.

#### N′‐Cinnamoylfuran‐2‐Carbohydrazide (4i)

Purified by flash column chromatography (Hex‐AcOEt 6 : 4) and obtained as a yellow solid in 32 % yield, mp 176–178 °C. ^**1**^ **H‐NMR** (DMSO‐*d6*, 400 MHz) δ: 10.41 (s, 1H, NH), 10.18 (s, 1H, NH), 7.87 (br s, 1H), 7.62–7.61 (m, 2H), 7.55 (d, *J*=15.6 Hz, 1H), 7.43–7.41 (m, 3H) 7.24 (br s, 1H), 6.71 (d, *J*=15.6 Hz, 1H), 6.66 (br s, 1H); ^**13**^ **C‐NMR** (100.6 MHz, DMSO‐*d6*) δ: 164.8, 157.4, 146.3, 146.0, 140.8, 134.7, 130.2, 129.3, 128.0, 119.4, 115.0, 112.2; **MS** (DART+) m/z [M+H]^+^: 257 m/z; **HRMS** *m/z* calcd for ^12^C_14_^1^H_13_^14^N_2_^16^O_3_ [M+H]^+^, 257.09262; found 257.09240; **IR** (ATR, cm^−1^): 3205, 3002, 2927, 1646, 1619, 1587, 1301, 756.

#### N‐(4‐Aminophenyl)furan‐2‐Carboxamide (10)

In a round‐bottom flask, furoic acid (0.82 mmol) and CDI (0.90 mmol) were dissolved in THF (1.7 mL) and stirred for 2 h at 45 °C. In another flask, 1,4‐diaminobenzene (1.49 mmol) was dissolved in THF (2 mL) and the solution of the activated carboxylic acid was added dropwise (1 drop per 30 s). The reaction was stirred at 45 °C for 18 h. The solvent was removed under vacuum and redissolved in AcOEt, washed with an aqueous solution of NaHCO_3_ (10 %), and dried with Na_2_SO_4_. The amide product was purified by flash column chromatography (Hex‐AcOEt 7 : 3). Compound **10** was obtained as a brown solid in 59 % yield. ^**1**^ **H‐NMR** (CDCl_3_, 400 MHz) δ: 7.94 (br s, 1H, NH amide), 7.48 (s, 1H), 7.42–7.40 (AA'BB'), 7.19 (d, *J*=3.2 Hz, 1H), 6.69–6.67 (AA'BB', 2H), 6.52 (dd, *J*=3.4 and 1.6 Hz, 1H), 3.64 (br s, NH_2_); ^**13**^ **C‐NMR** (100 MHz, DMSO‐*d6*) δ: 156.1, 148.2, 144.0, 143.7, 128.8, 122.0, 115.6, 114.8, 112.6; **MS** (DART+) m/z [M+H]^+^: 203 m/z; **HRMS** *m/z* calcd for ^12^C_11_^1^H_11_^14^N_2_^16^O_2_ [M+H]^+^, 203.08205; found 203.08177.

### General Synthesis of Diamides 5a–f.

The corresponding benzoic acid (0.15 mmol) reacted with CDI (0.18 mmol) in THF (0.4 mL) for 2 h at 45 °C. After the formation of the reactive intermediate, amine **10** (0.15 mmol) was added and stirred for 20 h at 45 °C. After the completion of the reaction (monitored by TLC), the solid was filtered and washed with DCM and AcOEt to afford the pure diamide.

#### N‐(4‐Benzamidophenyl)furan‐2‐Carboxamide (5a)

Obtained as a white solid in 84 % yield, mp 284–286 °C. ^**1**^ **H‐NMR** (DMSO‐*d6*, 300 MHz) δ: 10.24 (s, 1H), 10.17 (s, 1H), 7.97–7.93 (m, 3H), 7.73 (br s, 4H), 7.61–7.51 (m, 3H), 7.32 (d, *J*=3.6 Hz, 1H), 6.70 (dd, *J*=3.6 and 1.8 Hz, 1H); ^**13**^ **C‐NMR** (75.5 MHz, DMSO‐*d6*) δ: 165.4, 156.1, 147.6, 145.6, 135.1, 135.0, 134.3, 131.5, 128.4, 127.6, 120.7, 114.6, 112.1; **MS** (DART+) m/z [M+H]^+^: 307 m/z; **HRMS** *m/z* calcd for ^12^C_18_^1^H_15_^14^N_2_^16^O^3^ [M+H]^+^, 307.10827; found 307.10814; **IR** (ATR, cm^−1^): 3327, 3137, 3055, 1647, 1537, 1313.

#### N‐(4‐(4‐Methylbenzamido)phenyl)furan‐2‐Carboxamide (5b)

Obtained as a white solid in 69 % yield, mp 276–278 °C. ^**1**^ **H‐NMR** (DMSO‐*d6*, 400 MHz) δ: 10.17 (s, 1H), 10.15 (s, 1H), 7.92 (dd, *J*=1.6 and 0.4 Hz, 1H), 7.88–7.86 (AA'BB', 2H), 7.75–7.69 (m, 4H), 7.33–7.31 (m, 3H), 6.69 (dd, *J*=3.4 and 1.6 Hz, 1H), 2.38 (s, 3H); ^**13**^ **C‐NMR** (DMSO‐*d6*, 100 MHz) δ: 165.2, 156.1, 147.6, 145.7, 141.6, 135.2, 134.2, 132.1, 129.0, 127.7, 120.7 (4 C), 114.6, 112.2, 21.0; **MS** (DART+) m/z [M+H]^+^: 321 m/z; **HRMS** *m/z* calcd for ^12^C_19_^1^H_17_^14^N_2_^16^O_3_ [M+H]^+^, 321.12392; found 321.12412; **IR** (ATR, cm^−1^): 3309, 3146, 1642, 1564, 1516, 1315, 853, 752.

#### N‐(4‐(4‐Methoxybenzamido)phenyl)furan‐2‐Carboxamide (5c)

Obtained as a white solid in 47 % yield, mp 275–277 °C. ^**1**^ **H‐NMR** (DMSO‐*d6*, 300 MHz) δ: 10.16 (s, 1H), 10.08 (s, 1H), 7.98–7.92 (m, 3H), 7.72 (s, 4H), 7.32 (d, *J*=3.6 Hz, 1H), 7.09–7.03 (AA'BB', 2H), 6.69 (dd, *J*=3.3 and 1.8 Hz, 1H), 3.84 (s, 3H); ^**13**^ **C‐NMR** (DMSO‐*d6*, 75.5 MHz) δ: 164.7, 161.9, 156.1, 147.6, 145.6, 135.3, 134.1, 129.6, 127.0, 120.67, 120.65, 114.5, 113.6, 112.1, 55.4; **MS** (DART+) m/z [M+H]^+^: 337 m/z; **HRMS** *m/z* calcd for ^12^C_19_^1^H_17_^14^N_2_^16^O_4_ [M+H]^+^, 337.11883; found 337.11823; **IR** (ATR, cm^−1^): 3320, 3129, 3003, 1648, 1537, 1406, 838.

#### N‐(4‐(4‐(Tert‐Butyl)benzamido)phenyl)furan‐2‐Carboxamide (5d)

Obtained as a white solid in 44 % yield, mp 263–264 °C. ^**1**^ **H‐NMR** (DMSO‐*d6*, 300 MHz) δ: 10.17 (s, 2H), 7.92 (dd, *J*=1.8 and 0.9 Hz, 1H), 7.91–7.87 (AA'BB', 2H), 7.72 (s, 4H), 7.56–7.53 (AA'BB', 2H), 7.32 (dd, *J*=3.6 and 0.9 Hz, 1H), 6.70 (dd, *J*=3.3 and 1.8 Hz, 1H), 1.32 (s, 9H); ^**13**^ **C‐NMR** (DMSO‐*d6*, 75.5 MHz) δ: 165.3, 156.1, 154.4, 147.6, 145.6, 135.2, 134.2, 132.3, 127.5, 125.2, 120.7, 120.6, 114.5, 112.1, 34.7, 31.0; **MS** (DART+) m/z [M+H]^+^: 363 m/z; **HRMS** *m/z* calcd for ^12^C_22_^1^H_23_^14^N_2_^16^O_3_ [M+H]^+^, 363.17087; found 363.17094; **IR** (ATR, cm^−1^): 3319, 3147, 2963, 2901, 1649, 1541, 1317, 844.

#### N‐(4‐(4‐Chlorobenzamido)phenyl)furan‐2‐Carboxamide (5e)

Obtained as an off‐ white solid in 43 % yield, mp 295–297 °C. ^**1**^ **H‐NMR** (DMSO‐*d6*, 400 MHz) δ: 10.32 (s, 1H, NH), 10.20 (s, 1H, NH), 7.98–7.96 (AA'BB', 2H), 7.90 (br s, 1H), 7.71 (s, 4H), 7.60–7.58 (AA'BB', 2H), 7.31 (d, *J*=3.6 Hz, 1H), 6.69 (dd, *J*=3.6 and 1.6 Hz, 1H); ^**13**^ **C‐NMR** (100.6 MHz, DMSO‐*d6*) δ: 164.5, 156.3, 147.7, 145.8, 136.5, 135.0, 134.6, 133.8, 129.7, 128.6, 121.0, 120.9, 114.8, 112.3; **MS** (DART+) m/z [M+H]^+^: 341 m/z; **HRMS** *m/z* calcd for ^12^C_18_^1^H_14_^35^Cl_1_^14^N_2_^16^O_3_ [M+H]^+^, 341.06929; found 341.06874; **IR** (ATR, cm^−1^): 3321, 2988, 1647, 1505, 826.

#### N‐(4‐(4‐Fluorobenzamido)phenyl)furan‐2‐Carboxamide (5f)

Obtained as a white solid in 56 % yield, mp 241–243 °C. ^**1**^ **H‐NMR** (DMSO‐*d6* and CDCl_3_, 400 MHz) δ: 10.29 (s, 1H), 10.09 (s, 1H), 8.06–8.01 (m, 2H), 7.83 (br s, 1H), 7.71 (s, 4H), 7.31–7.56 (m, 3H), 6.64 (dd, *J*=3.4 and 1.6 Hz, 1H); ^**13**^ **C‐NMR** (DMSO‐*d6* and CDCl_3_, 100 MHz) δ: 164.1, 164.0, (d, *J*=248.5 Hz), 156.0, 147.6, 145.2, 134.8, 134.3, 131.3 (d, *J*=3.6 Hz), 130.2 (d, *J*=8.6 Hz), 120.6, 120.5, 115.0 (d, *J*=21.6 Hz), 114.3, 111.9; ^**19**^ **F‐NMR** (DMSO‐*d6* and CDCl_3_, 376 MHz) δ: −108.8; **MS** (DART+) m/z [M+H]^+^: 325 m/z; **HRMS** *m/z* calcd for ^12^C_18_^1^H_14_^19^F_1_^14^N_2_^16^O_3_ [M+H]^+^, 325.09885; found 325.09834; **IR** (ATR, cm^−1^): 3327, 3151, 1654, 1564, 1241.

### Synthesis of 3‐(Furan‐2‐Carboxamido)benzoic Acid (11)

In a round‐bottom flask, furan‐2‐carboxylic acid (**8**, 0.9 mmol) and CDI (1.34 mmol) were dissolved in THF (2 mL) and heated at 45 °C for 2 h. After the formation of the intermediate, 3‐aminobenzoic acid (1.16 mmol) was added and led to react for 20 h at 45 °C. THF was removed under vacuum and dissolved in AcOEt; the organic phase was washed with aqueous NaHCO_3_ (10 %) and HCl (10 %). The crude was purified by flash column chromatography and the acid **11** was isolated as a light brown solid in 47 % yield.

### General Method for the Synthesis of Carboxamides 6a–e

Benzoic acid **11** (0.22 mmol) and CDI (0.33 mmol) were dissolved in THF (0.5 mL) The mixture was heated at 45 °C for 2 h. Then, the corresponding aniline (0.24 mmol) was added and heated for 20 h more. THF was removed under vacuum and dissolved in AcOEt; the organic phase was sequentially washed with aqueous NaHCO_3_ (10 %) and HCl (10 %). The crude was purified by flash column chromatography to obtain the corresponding carboxamides **6 a**–**e**.

#### N‐(3‐(Phenylcarbamoyl)phenyl)furan‐2‐Carboxamide (6a)

Purified by flash column chromatography (Hex‐AcOEt 7 : 3) and obtained as a light brown solid in 87 % yield, mp 235–237 °C. ^**1**^ **H‐NMR** (DMSO‐*d6*, 300 MHz) δ: 10.44 (s, 1H), 10.14 (s, 1H), 7.99–7.91 (m, 5H), 7.78 (d, *J*=8.1 Hz, 2H), 7.40 (d, *J*=3.6 Hz, 1H), 7.38–7.32 (m, 2H), 7.09 (t, *J*=7.2 Hz, 1H), 6.73 (dd, *J*=3.6 and 1.8 Hz, 1H); ^**13**^ **C‐NMR** (DMSO‐*d6*, 75.5 MHz) δ: 164.9, 156.4, 147.3, 146.1, 141.6, 139.3, 129.7, 128.6, 128.5, 123.5, 120.3, 119.5, 115.3, 112.3; **MS** (DART+) m/z [M+H]^+^: 306 m/z; **HRMS** *m/z* calcd for ^12^C_18_^1^H_15_^14^N_2_^16^O_3_ [M+H]^+^, 307.10827; found 307.10812; **IR** (ATR, cm^−1^): 3338, 3311, 3113, 2998, 1666, 1649, 1596, 1439, 1403, 760.

#### N‐(3‐((4‐Methylphenyl)carbamoyl)phenyl)furan‐2‐Carboxamide (6b)

Purified by flash column chromatography (Hex‐AcOEt 6 : 5) and obtained as a beige solid in 57 % yield, mp 202–204 °C. ^**1**^ **H‐NMR** (DMSO‐*d6*, 300 MHz) δ: 10.37 (s, 1H), 10.19 (s, 1H), 8.26 (t, *J*=2.1 Hz, 1H), 7.99 (ddd, *J*=5.9, 2.3, and 1.2 Hz, 1H), 7.95 (dd, *J*=1.8 and 0.9 Hz, 1H), 7.69–7.63 (m, 3H), 7.49 (t, *J*=8.1 Hz, 1H), 7.39 (dd, *J*=3.6 and 0.9 Hz, 1H), 7.18–7.15 (AA'BB', 2H), 6.72 (dd, *J*=3.5 and 1.8 Hz, 1H), 2.28 (s, 3H); ^**13**^ **C‐NMR** (DMSO‐*d6*, 75.5 MHz) δ: 165.3, 156.4, 147.3, 145.9, 138.7, 136.6, 135.8, 132.6, 129.0, 128.7, 123.2, 122.6, 120.4, 119.9, 114.9, 112.2, 20.5; **MS** (DART+) m/z [M+H]^+^: 321 m/z; **HRMS** *m/z* calcd for ^12^C_19_^1^H_17_^14^N_2_^16^O_3_ [M+H]^+^, 321.12392; found 321.12380; **IR** (ATR, cm^−1^): 3386, 3316, 3141, 3116, 2918, 2855, 1644, 1610, 1518, 1320, 1019, 775.

#### N‐(3‐((4‐Methoxyphenyl)carbamoyl)phenyl)furan‐2‐Carboxamide (6c)

Purified by flash column chromatography (Hex‐AcOEt 55 : 45) and obtained as a white solid in 40 % yield, mp 209–211 °C. ^**1**^ **H‐NMR** (DMSO‐*d6*, 300 MHz) δ: 10.37 (s, 1H), 10.15 (s, 1H), 8.27 (br s, 1H), 7.99–7.95 (m, 2H), 7.69–7.66 (m, 3H), 7.49 (t, *J*=7.8 Hz, 1H), 7.39 (d, *J*=3.6 Hz, 1H), 6.95–6.92 (AA'BB', 2H), 6.71 (dd, *J*=3.6 and 1.8 Hz, 1H), 3.75 (s, 3H); ^**13**^ **C‐NMR** (DMSO‐*d6*, 75.5 MHz) δ: 165.1, 156.4, 155.6, 147.3, 145.9, 138.7, 135.8, 132.2, 128.7, 123.1, 122.6, 122.0, 119.9, 114.9, 113.8, 112.2, 55.2; **MS** (DART+) m/z [M+H]^+^: 337 m/z; **HRMS** *m/z* calcd for ^12^C_19_^1^H_17_^14^N_2_^16^O_4_ [M+H]^+^, 337.11883; found 337.11888; **IR** (ATR, cm^−1^): 3293, 3138, 3068, 3002, 2957, 1638, 1656, 1567, 1532, 1242, 1029.

#### N‐(3‐((4‐(Tert‐Butyl)phenyl)carbamoyl)phenyl)furan‐2‐Carboxamide (6d)

Purified by flash column chromatography (Hex‐AcOEt 7 : 3) and obtained as a white solid in 64 % yield, mp 198–200 °C. ^**1**^ **H‐NMR** (DMSO‐*d6*, 300 MHz) δ: 10.40 (s, 1H), 10.22 (s, 1H), 8.26 (t, *J*=1.8 Hz, 1H), 7.98 (ddd, *J*=5.9, 2.3, and 1.2 Hz, 1H), 7.96 (dd, *J*=1.7 and 0.9 Hz, 1H), 7.70–7.66 (m, 3H), 7.49 (t, *J*=7.8 Hz, 1H), 7.40–7.34 (m, 3H), 6.72 (dd, *J*=3.6 and 1.8 Hz, 1H), 1.28 (s, 9H); ^**13**^ **C‐NMR** (DMSO‐*d6*, 75.5 MHz) δ: 165.4, 156.4, 147.4, 146.1, 146.0, 138.8, 136.6, 135.8, 128.8, 125.3, 123.3, 122.7, 120.2, 120.0, 115.1, 112.3, 34.1, 31.3; **MS** (DART+) m/z [M+H]^+^: 363 m/z; **HRMS** *m/z* calcd for ^12^C_22_^1^H_23_^14^N_2_^16^O_3_ [M+H]^+^, 363.17087; found 363.17077; **IR** (ATR, cm^−1^): 3292, 3109, 2951, 2901, 1646, 1609, 1565, 681.

#### N‐(3‐((4‐Chlorophenyl)carbamoyl)phenyl)furan‐2‐Carboxamide (6e)

Purified by flash column chromatography (Hex‐AcOEt 7 : 3) and obtained as an off‐white solid in 93 % yield, mp 221–223 °C. ^**1**^ **H‐NMR** (DMSO‐*d6*, 300 MHz) δ: 10.42 (s, 1H), 10.41 (s, 1H), 8.28 (t, *J*=1.8 Hz, 1H), 7.99 (ddd, *J*=8.1, 2.3 and 1.2, 1H), 7.96 (dd, *J*=1.8 and 0.9 Hz, 1H), 7.84–7.79 (AA'BB', 2H), 7.68 (dt, *J*=8.1 and 1.2 Hz, 1H), 7.51 (t, *J*=8.1 Hz, 1H), 7.44–7.40 (AA'BB', 2H), 7.39 (dd, *J*=3.6 and 0.9 Hz, 1H), 6.72 (dd, *J*=3.6 and 1.8 Hz, 1H); ^**13**^ **C‐NMR** (DMSO‐*d6*, 75.5 MHz) δ: 165.7, 156.4, 147.3, 146.0, 138.8, 138.2, 135.5, 128.8, 128.6, 127.4, 123.5, 122.8, 121.9, 119.9, 115.1, 112.3; **MS** (DART+) m/z [M+H]^+^: 341 m/z; **HRMS** *m/z* calcd for ^12^C_18_^1^H_14_^35^Cl_1_^14^N_2_^16^O_3_ [M+H]^+^, 341.06929; found 341.06961; **IR** (ATR, cm^−1^): 3294, 2954, 2924, 1642, 1609, 1517, 754.

### General Method for the Synthesis of Acetamides 13a–e

The corresponding aniline (0.68 mmol) and triethylamine (0.8 mmol) were dissolved in DCM (1 mL) and cooled to 0 °C. Then, bromoacetyl chloride (0.8 mmol) was added dropwise and stirred for 2 h. After the consumption of the starting material, a solution of aqueous NaHCO_3_ (10 %, 5 mL) was added and stirred for another 10 min. The organic phase was separated, dried over Na_2_SO_4,_ and purified by flash column chromatography.

### General Method for the Synthesis of Azides 14a–e

In a round‐bottom flask, the corresponding acetamide **13a–e** (0.2 mmol) and sodium azide (0.4 mmol) were dissolved in DMSO (0.2 mL) and stirred at 80 °C for 2 h. After this time, water (5 mL) was added and extracted with AcOEt. The organic phase was evaporated, and the corresponding organic azide was used without purification.

#### N‐(Prop‐2‐Yn‐1‐Yl)furan‐2‐Carboxamide (15)

Furoic acid (0.45 mmol), 4‐dimethylaminopyridine (0.13 mmol), and propargylamine (0.54 mmol) were dissolved in DCM (2.2 mL) and stirred at 0 °C for 10 min. After that, dicyclohexylcarbodiimide (0.54 mmol) was added and the mixture was allowed to react for 20 h. After the consumption of the reaction (monitored by TLC), the excess DCU was filtered. The organic phase was evaporated, and acetonitrile (2 mL) was added and cooled for 30 min. The solid was filtered and the solution was purified by flash column chromatography (Hex‐AcOEt 7 : 3), obtaining a beige solid in 62 % yield. ^**1**^ **H‐NMR** (CDCl_3_, 300 MHz) δ: 7.44 (d, *J*=2.1 Hz, 1H), 7.13 (d, *J*=3.3 Hz, 1H), 6.56 (br s, 1H, NH), 6.49 (dd, *J*=3.6 and 1.8 Hz, 1H), 4.22 (dd, *J*=5.4 and 2.7 Hz, 2H), 2.27 (t, *J*=2.7 Hz, 1H); ^**13**^ **C‐NMR** (CDCl_3,_ 75.5 MHz) δ: 158.0, 147.6, 144.3, 114.9, 112.3, 79.4, 72.0, 29.0; **MS** (DART+) m/z [M+H]^+^: 150 m/z.

### General Method for the Synthesis of Triazoles 7a–e.

In a round‐bottom flask, the unpurified azide (**14 a**–**e**, 0.14 mmol), alkyne **15** (0.09 mmol), CuSO_4_•5H_2_O (0.018 mmol), and sodium ascorbate (0.018 mmol) were suspended in DMSO (0.3 mL). The mixture was stirred vigorously for 10 h; then, water (5 mL) was added. The solid was filtered and purified by column chromatography to afford the corresponding triazole.

#### N‐((1‐(2‐Oxo‐2‐(Phenylamino)ethyl)‐1H‐1,2,3‐Triazol‐4‐Yl)methyl)furan‐2‐Carboxamide (7a)

Purified by flash column chromatography (AcOEt‐MeOH 9 : 1 to 95 : 5) and obtained as a white solid in 30 % yield, mp 216–219 °C. ^**1**^ **H‐NMR** (DMSO‐*d6*, 300 MHz) δ: 10.44 (s, 1H; NH), 8.93 (t, *J*=6.0 Hz, 1H, NH), 7.99 (br s, 1H), 7.83 (br s, 1H), 7.58 (d, *J*=8.1 Hz, 2H), 7.36–8.30 (m, 2H), 7.14 (t, *J*=3.3 Hz, 1H), 7.10–7.05 (m, 1H), 6.62 (br s, 1H), 5.30 (s, 2H), 4.50 (d, *J*=6.3 Hz, 2H); ^**13**^ **C‐NMR** (76 MHz, DMSO‐*d6*) δ: 164.3, 157.8, 147.8, 145.1, 144.9, 138.4, 128.9, 124.7, 123.8, 119.2, 113.6, 111.8, 52.2, 34.1; **MS** (DART+) m/z [M+H]^+^: 326 m/z; **HRMS** *m/z* calcd for ^12^C_16_^1^H_16_^14^N_5_^16^O_3_ [M+H]^+^, 326.12531; found 326.12557; **IR** (ATR, cm^−1^): 3266, 3136, 3067, 2975, 2939, 1660, 1677, 1447, 747.

#### N‐((1‐(2‐Oxo‐2‐(P‐Tolylamino)ethyl)‐1H‐1,2,3‐Triazol‐4‐Yl)methyl)furan‐2‐Carboxamide (7b)

Purified by flash column chromatography (AcOEt‐MeOH 8 : 2) and obtained as an off‐white solid in 30 % yield, mp 214–216 °C. ^**1**^ **H‐NMR** (DMSO‐*d6*, 400 MHz) δ: 10.34 (s, 1H, NH), 8.92 (t, *J*=6.0 Hz, 1H, NH), 7.97 (s, 1H), 7.83 (dd, *J*=1.9 and 0.8 Hz, 1H), 7.47–7.43 (AA'BB', 2H), 7.15–7.10 (m, 3H), 6.62 (dd, *J*=3.4 and 1.8 Hz, 1H), 5.26 (s, 2H), 4.48 (d, *J*=5.9 Hz, 2H), 3.25 (s, 3H); ^**13**^ **C‐NMR** (100 MHz, DMSO‐*d6*) δ: 164.0, 157.8, 147.8, 145.1, 144.8, 135.9, 132.8, 129.3, 124.6, 119.2, 113.6, 111.8, 52.1, 34.1, 20.4; **MS** (DART+) m/z [M+H]^+^: 340 m/z; **HRMS** *m/z* calcd for ^12^C_17_^1^H_18_^14^N_5_^16^O_3_ [M+H]^+^, 340.14096; found 340.14109; **IR** (ATR, cm^−1^): 3392, 3257, 3149, 3061, 2940, 1672, 1660, 1537, 1024, 765.

#### N‐((1‐(2‐((4‐Methoxyphenyl)amino)‐2‐Oxoethyl)‐1H‐1,2,3‐Triazol‐4‐Yl)methyl)furan‐2‐Carboxamide (7c)

Purified by flash column chromatography (AcOEt‐MeOH 8 : 2) and obtained as a white solid in 53 % yield, mp 219–220 °C. ^**1**^ **H‐NMR** (DMSO‐*d6*, 300 MHz) δ: 10.31 (s, 1H, NH), 8.92 (t, *J*=5.7 Hz, 1H, NH), 7.97 (s, 1H), 7.83 (br s, 1H), 7.50–7.47 (AA'BB', 2H), 7.14 (d, *J*=3.6 Hz, 1H), 6.91–6.88 (AA'BB', 2H), 6.62 (br s, 1H), 5.25 (s, 2H), 4.48 (d, *J*=5.7 Hz, 2H), 3.72 (s, 3H); ^**13**^ **C‐NMR** (76 MHz, DMSO‐*d6*) δ: 163.7, 157.8, 155.5, 147.8, 145.1, 144.8, 131.5, 124.6, 121.9, 120.8, 114.0, 113.5, 111.8, 55.2, 52.1, 34.1; **MS** (DART+) m/z [M+H]^+^: 356 m/z; **HRMS** *m/z* calcd for ^12^C_17_^1^H_18_^14^N_5_^16^O_4_ [M+H]^+^, 356.13588; found 356.13589; **IR** (ATR, cm^−1^): 3266, 3144, 3058, 2940, 2834, 1659, 1575, 1306, 1026, 826.

#### N‐((1‐(2‐((4‐(Tert‐Butyl)phenyl)amino)‐2‐Oxoethyl)‐1H‐1,2,3‐Triazol‐4‐Yl)methyl)furan‐2‐Carboxamide (7d)

Purified by flash column chromatography (AcOEt‐MeOH 8 : 2) and obtained as a light brown solid in 79 % yield, mp 173–175 °C. ^**1**^ **H‐NMR** (DMSO‐*d6*, 300 MHz) δ: 10.36 (s, 1H, NH), 8.92 (t, *J*=6.0 Hz, 1H, NH), 7.97 (s, 1H), 7.83 (dd, *J*=1.8 and 0.9 Hz, 1H), 7.50–7.46 (AA'BB', 2H), 7.36–7.32 (AA'BB', 2H), 7.14–7.12 (m, 1H), 6.62 (dd, *J*=3.3 and 1.8 Hz, 1H), 5.27 (s, 2H), 4.48 (d, *J*=6.0 Hz, 2H), 1.25 (s, 9H); ^**13**^ **C‐NMR** (76 MHz, DMSO‐*d6*) δ: 164.0, 157.8, 147.8, 146.1, 145.1, 144.8, 135.9, 125.5, 124.6, 119.0, 113.5, 111.8, 52.9, 34.1, 34.0, 31.2; **MS** (DART+) m/z [M+H]^+^: 382 m/z; **HRMS** *m/z* calcd for ^12^C_20_^1^H_24_^14^N_5_^16^O_3_ [M+H]^+^, 382.18791; found 382.18783; **IR** (ATR, cm^−1^): 3319, 3196, 3124, 3066, 2955, 2905, 1681, 1642, 1528, 830.

#### N‐((1‐(2‐((4‐Chlorophenyl)amino)‐2‐Oxoethyl)‐1H‐1,2,3‐Triazol‐4‐Yl)methyl)furan‐2‐Carboxamide (7e)

Purified by flash column chromatography (AcOEt‐MeOH 9 : 1) and obtained as a white solid in 36 % yield, mp 235–236 °C. ^**1**^ **H‐NMR** (DMSO‐*d6*, 300 MHz) δ: 10.58 (s, 1H, NH), 8.93 (t, *J*=6.0 Hz, 1H, NH), 7.97 (s, 1H), 7.82 (dd, *J*=1.8 and 0.8 Hz, 1H), 7.62–7.57 (AA'BB', 2H), 7.40–7.35 (AA'BB', 2H), 7.13 (t, *J*=3.5 and 0.8 Hz, 1H), 6.62 (dd, *J*=3.5 and 1.8 Hz, 1H), 5.29 (s, 2H), 4.48 (d, *J*=6.0 Hz, 2H); ^**13**^ **C‐NMR** (76 MHz, DMSO‐*d6*) δ: 164.5, 157.8, 147.8, 145.2, 144.9, 137.4, 128.9, 127.4, 124.7, 120.9, 113.6, 111.9, 52.1, 34.1; **MS** (DART+) m/z [M+H]^+^: 360 m/z; **HRMS** *m/z* calcd for ^12^C_16_^1^H_15_^35^Cl_1_^14^N_5_^16^O_3_ [M+H]^+^, 360.08634; found 360.08633; **IR** (ATR, cm^−1^): 3274, 3146, 3062, 2986, 2944, 1682, 1637, 1571, 1249.

### Synthesis of C‐30

#### Preparation of 3,5‐Dibromolevulinic Acid

In a round‐bottom flask, levulinic acid (50 mg, 431 μmol) was dissolved in CHCl_3_ (0.4 mL) and HBr solution 33 wt. % in AcOH (4 drops) was added. Subsequently, a solution of Br_2_ (48.5 μL, 947 μmol) in CHCl_3_ (0.4 mL) was added dropwise for 30 minutes at room temperature. Next, the solution was stirred for 30 min at 50 °C, then at reflux for 1 h. Afterward, the mixture was successively washed with H_2_O, Na_2_S_2_O_3_ solution, and brine. The organic phase was dried with Na_2_SO_4_ and evaporated under reduced pressure. The crude was used in the next step without further purification.

#### Preparation of (Z)‐4‐Bromo‐5‐(Bromomethylene)furan‐2(5H)‐One (2, C‐30)

3,5‐dibromolevulinic acid (80 mg, 292 μmol) was dissolved in a round‐bottom flask in H_2_SO_4_ (1 mL). The mixture was stirred for 20 minutes at 110–120 °C. After this time, the mixture was cooled to room temperature and then slowly poured onto crushed ice. The formed emulsion was extracted with DCM; the organic phase was dried with Na_2_SO_4_ and evaporated under reduced pressure. Finally, the desired product was isolated by flash column chromatography (Hex‐AcOEt 95 : 5), affording white crystals in 52 % yield. Spectroscopic data are consistent with previous reports. ^**1**^ **H‐NMR** (400 MHz, CDCl_3_) δ: 6.50 (d, *J*=0.5 Hz, 1H), 6.41 (d, *J*=0.5 Hz, 1H); ^**13**^ **C‐NMR** (100 MHz, CDCl_3_) δ: 165.5, 151.2, 135.3, 121.1, 93.8; MS (DART^+^) m/z: [M+H]^+^; 253.

### Microdilution Method for Antimicrobial Screening

The four series were evaluated following the methodology described by the CLSI M07‐A10 using a microdilution test. Briefly, *P. aeruginosa* (PA01) was grown in Müeller‐Hinton broth overnight. Subsequently, the bacterial suspension was adjusted to an optical density (OD_600_ nm) of 0.08–0.13 equivalent to 1.5×10^8^ UFC/mL, and then diluted 1/20 in the same media. Tested compounds were dissolved in DMSO at 2.5 mM. Microdilution assays were carried out in a 96‐well plate at a total volume of 100 μL. To each well, 10 μL of diluted bacterial suspension, 2 μL of molecules (final concentration of 50 μM), and 88 μL of Müeller‐Hinton broth were added, and the OD_600_ nm was measured (t0). Afterward, the plates were incubated at 37 °C for 24 h and measured again (t24, OD600 nm). Data are reported as the percentage of inhibition. Gentamicin was used as a positive control.

### Biofilm Staining and Quantification

After evaluating the growth inhibition, the microplates were poured into a recipient with water and sodium hypochlorite and finally washed with water three times. After drying, crystal violet (0.75 %, 100 μL) was added into each hole of the microplate for 15 min at room temperature. The excess of crystal violet was eliminated in a recipient containing water and ethanol, and the microplates were washed with water and dried. Finally, acetic acid at 30 % (100 μL) was added for 15 min and was quantified using a microplate reader at 570 nm. Biofilm formation percentage was calculated with the equation:

$$\%\mathrm{biofilm}\;\mathrm{formation}=(\mathrm{biofilm}\;\mathrm{formed}\;\mathrm{in}\;\mathrm{the}\;\mathrm{presence}\;\mathrm{of}\mathrm{furan}\text{-}2\text{-}\mathrm{carboxamides}/\;\mathrm{biofilm}\;\mathrm{formed}\;\mathrm{without}\;\mathrm{the}{\mathrm{presence}\;\mathrm{of}\;\mathrm{furanones})}^{*}100$$

The percentage of inhibition was calculated with the following equation:

%ofbiofilminhibition=100%-%biofilmformation

### Quantification of Virulence FactorsInoculation

In a 25x200 mm culture tube with a screw cap, 5 μL of stock solution (50 mM) was diluted with 5 mL of LB medium to obtain a final concentration of 50 μM. This step was repeated for each tested compound, solvent (DMSO), and blank (DMSO). Simultaneously, a 1 : 10 solution of a *Pseudomonas aeruginosa* culture was prepared and its OD600 (optic density at 600 nm) was measured. Next, the amount of bacteria culture (equivalent to 1x10^9^ UFC/mL) was determined with Equation 1, and added to each culture tube, except to the blank tube. Finally, test cultures were maintained at 37 °C for 20 h, with moderate shaking (200 rpm). The procedure was performed twice.

(1) $$V_{2}=\frac{\left(0.05\right)\left(5000\;\mu L\right)\;}{\left(OD\;measured\right)\left(10\right)}$$

### Protease Inhibition Assay

1.2 mL of the inoculated medium with *P. aeruginosa* was transferred to an Eppendorf tube and centrifuged (1300 rpm×2 min). Then, 5 μL of the supernatant and 100 μL of the substrate (1.25 % of azocasein in protease buffer) were added to another Eppendorf tube and incubated at 37 °C with shaking at 200 rpm for 35 minutes.

After that, a third series of Eppendorf tubes was prepared with 200 μL of a solution of HNO_3_ (1 %) and 50 μL of the previous incubated sample; then the tubes were centrifuged at 13,000 rpm for 2 minutes. Finally, 50 μL of the supernatant and 150 μL of a solution of NaOH (0.5 %) were transferred to a 96‐well plate (1 sample per well). The percentage of inhibition was determined by colorimetric quantification using a microplate reader at 440 nm.

### Pyocyanin Inhibition Test

The pyocyanin inhibition was determined using the same supernatant of the previous assay. First, the supernatant (400 μL) and CHCl_3_ (210 μL) were added to an Eppendorf tube, and the mixture was vigorously shaken with a Vortex for 1 minute. Next, the tubes were centrifuged at 13,000 rpm for 5 minutes. Subsequently, 150 μL of the organic phase (CHCl_3_) was neutralized with HCl (400 μL, 0.1 N). Again, the biphasic mixture was vigorously shaken with a Vortex for 1 minute and centrifuged at 13,000 rpm for 5 minutes. Finally, 300 μL of the aqueous phase was added to a 96‐well plate (1 sample per well), and the absorbance was measured with a microplate reader at 520 nm.

## Conflict of Interests

The authors declare no conflict of interest.

1

## Supporting information

As a service to our authors and readers, this journal provides supporting information supplied by the authors. Such materials are peer reviewed and may be re‐organized for online delivery, but are not copy‐edited or typeset. Technical support issues arising from supporting information (other than missing files) should be addressed to the authors.

Supporting Information

## Data Availability

The data that support the findings of this study are available from the corresponding author upon reasonable request.

## References

[1] C. H. N. Barros , E. Casey , ACS Appl. Nano Mater. 2020, 3, 8537–8556.

[2] J. A. Shapiro , S. J. Post , G. C. Smith , W. M. Wuest , Org. Lett. 2023, 25, 9243–9248. 38155597 10.1021/acs.orglett.3c03993 PMC10758118

[3] P. Shree , C. K. Singh , K. K. Sodhi , J. N. Surya , D. K. Singh , Med. Microecol. 2023, 16, 100084.

[4] H. Ma , J. D. Bryers , Appl. Microbiol. Biotechnol. 2013, 97, 317–328. 22669634 10.1007/s00253-012-4179-9 PMC3465625

[5] C. E. Flynn , J. Guarner , Mod. Pathol. 2023, 36, 100249. 37353202 10.1016/j.modpat.2023.100249

[6] C. de la Fuente-Núñez , M. H. Cardoso , E. de Souza Cândido , O. L. Franco , R. E. W. Hancock , Biochim. Biophys. Acta 2016, 1858, 1061–1069. 26724202 10.1016/j.bbamem.2015.12.015 PMC4809770

[7] A. Valliammai , A. Selvaraj , P. Mathumitha , C. Aravindraja , S. K. Pandian , Mater. Sci. Eng. C 2021, 121, 111863. 10.1016/j.msec.2021.111863 33579493

[8] S. Qin , W. Xiao , C. Zhou , Q. Pu , X. Deng , L. Lan , H. Liang , X. Song , M. Wu , Sig. Transduct. Target Ther. 2022, 7, 1–27. 10.1038/s41392-022-01056-1 PMC9233671 35752612

[9] Z. Pang , R. Raudonis , B. R. Glick , T.-J. Lin , Z. Cheng , Biotechnol. Adv. 2019, 37, 177–192. 30500353 10.1016/j.biotechadv.2018.11.013

[10] A. Vetrivel , P. Vetrivel , K. Dhandapani , S. Natchimuthu , M. Ramasamy , S. Madheswaran , R. Murugesan , Int. Microbiol. 2023, 26, 851–868. 36806045 10.1007/s10123-023-00338-0

[11] I. M. Hussaini , O. A. Oyewole , M. A. Sulaiman , A. I. Dabban , A. N. Sulaiman , R. Tarek , Res. Microbiol. 2024, 175, 104111. 37844786 10.1016/j.resmic.2023.104111

[12] M. Bové , X. Bao , A. Sass , A. Crabbé , T. Coenye , Antimicrob. Agents Chemother. 2021, 65, e0041321. 33903100 10.1128/AAC.00413-21 PMC8373219

[13] A.-C. Gómez , T. Lyons , U. Mamat , D. Yero , M. Bravo , X. Daura , O. Elshafee , S. Brunke , C. G. M. Gahan , M. O'Driscoll , I. Gibert , T. P. O'Sullivan , Eur. J. Med. Chem. 2022, 242, 114678. 36037789 10.1016/j.ejmech.2022.114678

[14] Á. Ramírez-Trinidad , E. Martínez-Solano , C. E. Tovar-Roman , M. García-Guerrero , J. A. Rivera-Chávez , E. Hernández-Vázquez , Bioorg. Med. Chem. Lett. 2024, 98, 129592. 38101651 10.1016/j.bmcl.2023.129592

[15] Z. Miao , L. Zhu , G. Dong , C. Zhuang , Y. Wu , S. Wang , Z. Guo , Y. Liu , S. Wu , S. Zhu , K. Fang , J. Yao , J. Li , C. Sheng , W. Zhang , J. Med. Chem. 2013, 56, 7902–7910. 24069881 10.1021/jm400906z

[16] D. Lengerli , K. Ibis , Y. Nural , E. Banoglu , Expert Opin. Drug Discovery 2022, 17, 1209–1236. 10.1080/17460441.2022.2129613 36164263

[17] S. Chatterjee , N. Kumar , H. Sehrawat , N. Yadav , V. Mishra , Curr. Res. Green Sust. Chem. 2021, 4, 100064.

[18] C. Deng , H. Yan , J. Wang , K. Liu , B. Liu , Y. Shi , Eur. J. Med. Chem. 2022, 244, 114888. 36334453 10.1016/j.ejmech.2022.114888

[19] F. Colombo , C. Tintori , A. Furlan , S. Borrelli , M. S. Christodoulou , R. Dono , F. Maina , M. Botta , M. Amat , J. Bosch , D. Passarella , Bioorg. Med. Chem. Lett. 2012, 22, 4693–4696. 22738633 10.1016/j.bmcl.2012.05.078

[20] M. Borowicz , D. M. Krzyżanowska , S. Jafra , J. Microbiol. Methods 2023, 204, 106656. 36526040 10.1016/j.mimet.2022.106656

[21] B. Morkunas , W. R. J. D. Galloway , M. Wright , B. M. Ibbeson , J. T. Hodgkinson , K. M. G. O'Connell , N. Bartolucci , M. D. Valle , M. Welch , D. R. Spring , Org. Biomol. Chem. 2012, 10, 8452. 23014532 10.1039/c2ob26501j

[22] A. S. B. Prasad , P. Shruptha , V. Prabhu , C. Srujan , U. Y. Nayak , C. K. R. Anuradha , L. Ramachandra , P. Keerthana , M. B. Joshi , T. S. Murali , K. Satyamoorthy , Lab. Invest. 2020, 100, 1532–1550. 32801335 10.1038/s41374-020-00478-1 PMC7683349

[23] D. W. Essar , L. Eberly , A. Hadero , I. P. Crawford , J. Bacteriol. 1990, 172, 884–900. 2153661 10.1128/jb.172.2.884-900.1990 PMC208517

[24] J. Charney , R. M. Tomarelli , J. Biol. Chem. 1947, 171, 501–505. 20272088

[25] R. A. Friesner , J. L. Banks , R. B. Murphy , T. A. Halgren , J. J. Klicic , D. T. Mainz , M. P. Repasky , E. H. Knoll , M. Shelley , J. K. Perry , D. E. Shaw , P. Francis , P. S. Shenkin , J. Med. Chem. 2004, 47, 1739–1749. 15027865 10.1021/jm0306430

[26] K. A. Simanek , M. L. Schumacher , C. P. Mallery , S. Shen , L. Li , J. E. Paczkowski , Nat. Commun. 2023, 14, 7986. 38042853 10.1038/s41467-023-43702-4 PMC10693556

[27] A. R. McCready , J. E. Paczkowski , B. R. Henke , B. L. Bassler , Proc. Natl. Acad. Sci. USA 2019, 116, 245–254. 30559209 10.1073/pnas.1817239116 PMC6320529

[28] J. Lee , L. Zhang , Protein Cell 2015, 6, 26–41. 25249263 10.1007/s13238-014-0100-x PMC4286720

[29] A. J. Manny , S. Kjelleberg , N. Kumar , R. de Nys , R. W. Read , P. Steinberg , Tetrahedron 1997, 53, 15813–15826.

