# Supplementary material for: Diversity‐Oriented Synthesis and Antibiofilm Evaluation of Furan‐2‐Carboxamides
Source: ChemMedChem. 2025 Jan 31;20(9):e202400879. doi: 10.1002/cmdc.202400879 (PMC12058234; doi:10.1002/cmdc.202400879)
Supplement: Supplementary file 1 — Supporting Information [file CMDC-20-e202400879-s001.pdf]

ChemMedChem

Supporting Information

Diversity-Oriented Synthesis and Antibiofilm Evaluation of Furan-2-Carboxamides

Ana C. Muñoz-Estrada, Cesar E. Tovar-Roman, Carlos D. García-Mejía, Rodolfo García-Contreras, and Eduardo Hernández-Vázquez*

–SUPPORTING INFORMATION–

Diversity-oriented synthesis and antibiofilm evaluation of furan-2-carboxamides.

Ana C. Muñoz-Estrada, Cesar E. Tovar-Roman, Carlos D. García-Mejía, Rodolfo García-Contreras, **Eduardo Hernández-Vázquez***.

CONTENT:

NMR spectra of carbohydrazides 4a-i **S2**

NMR spectra of diamides 5a-f **S23**

NMR spectra of amidoacylbenzamides 6a-e **S38**

NMR spectra of triazoles 7a-e **S50**

Figure S1 .... **S62**

Figure S2 .... **S63**

Figure S3 .... **S64**

Figure S4 .... **S65**

NMR SPECTRA OF CARBOHYDRAZIDES 4a-i

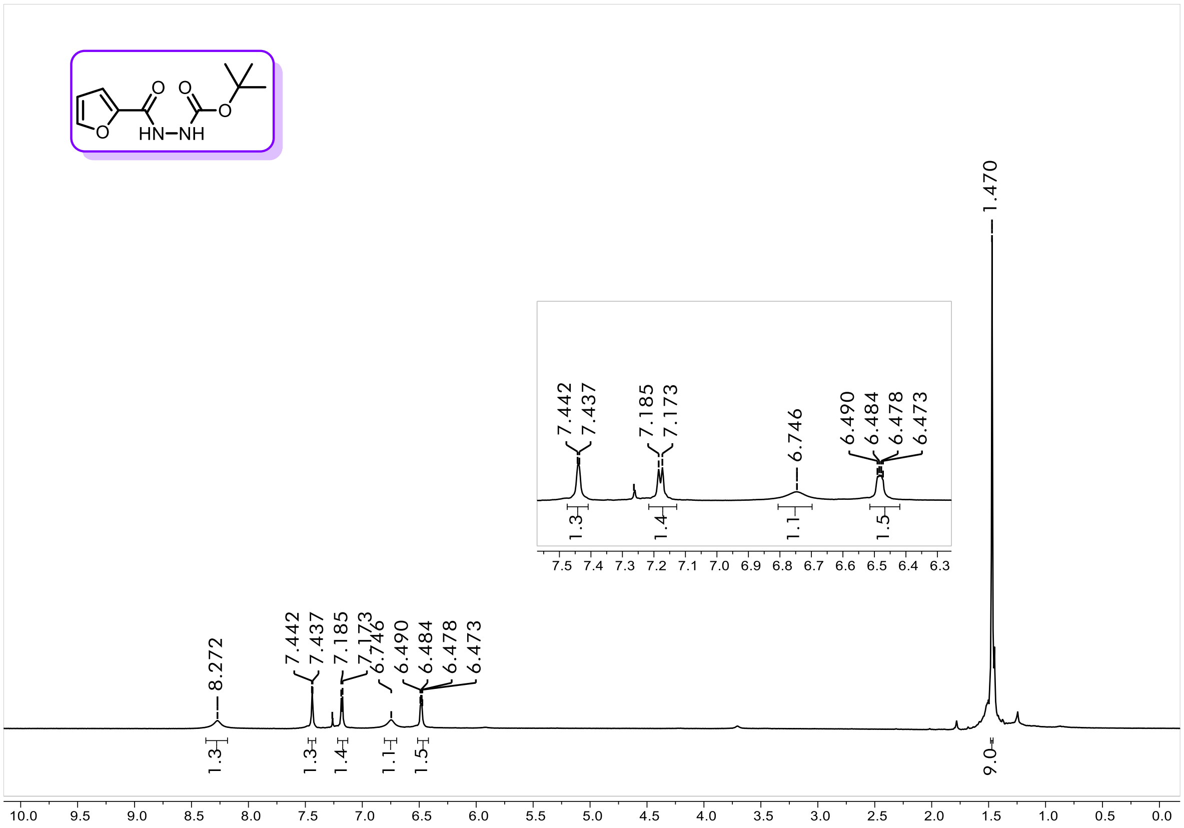

Spectrum 1. ^{1}H-NMR of *tert*-butyl 2-(furan-2-carbonyl)hydrazine-1-carboxylate **(9).**

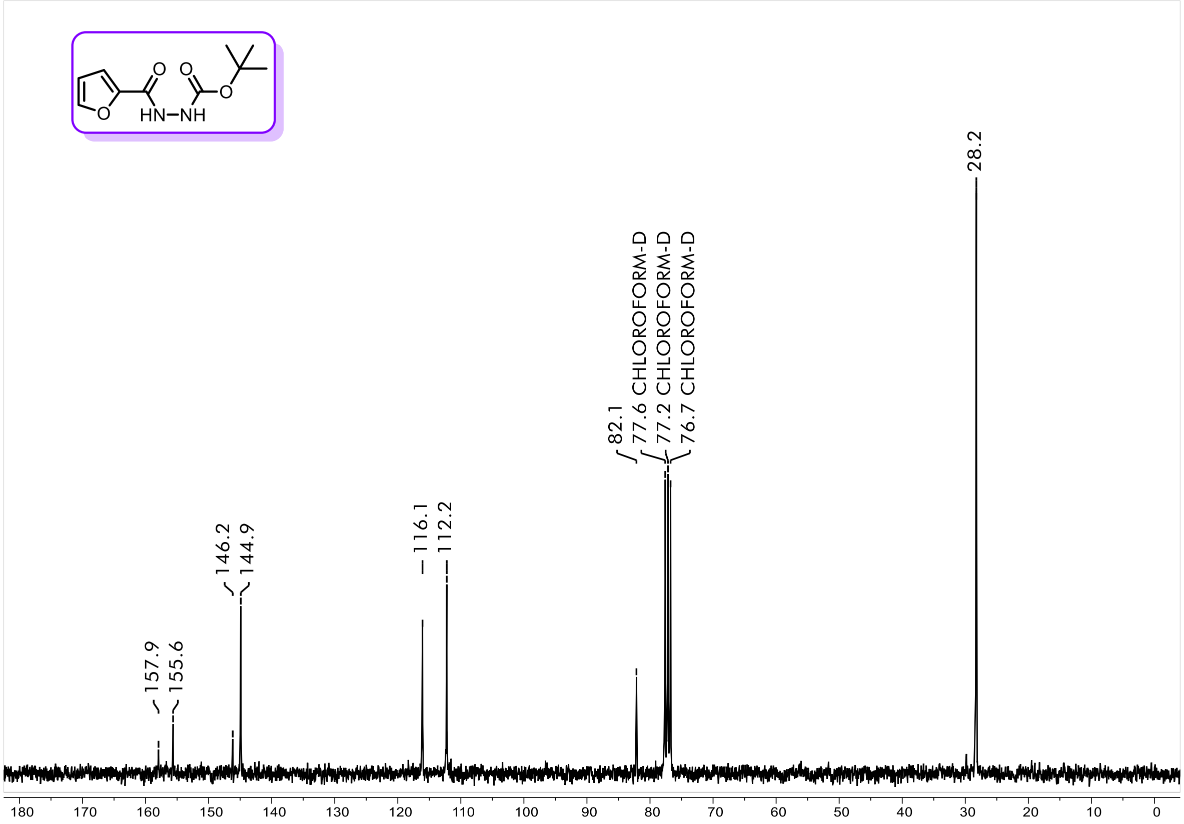

Spectrum 2. ^{13}C-NMR of *tert*-butyl 2-(furan-2-carbonyl)hydrazine-1-carboxylate **(9).**

Description:
Ionization Mode:ESI+
History:Determine m/z[Peak Detect[Centroid,30,Area];Correct Base[];Smooth[5]];Correct Base[5.0%];Average(MS[...

Mass Calibration data:Cal_PEG_600
Created:3/1/2024 10:57:46 AM
Created by:AccuTOF

Charge number:1
Element:^{12}C:0 .. 16, ^{1}H:0 .. 30, ^{14}N:2 .. 2, ^{16}O:4 .. 4

Tolerance:4.00(ppm), 5.00 .. 15.00(mmu)

Unsaturation Number:-1.0 .. 40.0 (Fraction:Both)

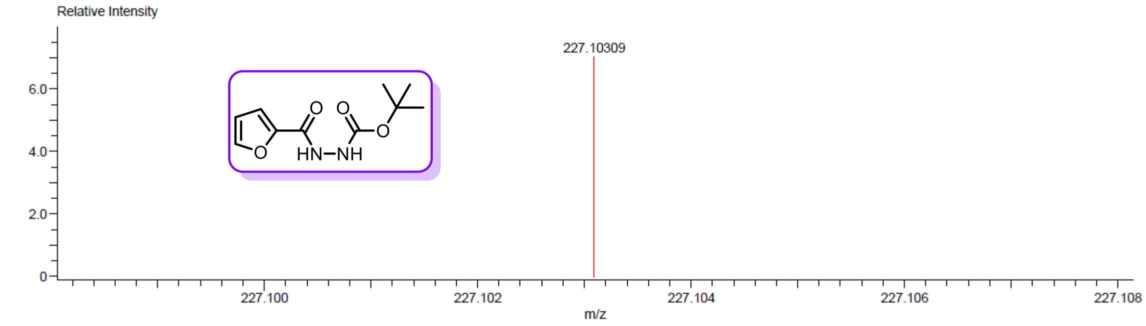

Mass	Intensity	Calc. Mass	Mass Difference (mmu)	Mass Difference (ppm)	Possible Formula	Unsaturation Number
227.10309	14337.18	227.10318	-0.09	-0.40	$^{12}C_{10}{}^{1}H_{15}{}^{14}N_2{}^{16}O_4$	4.5

Spectrum 3. HRMS of *tert*-butyl 2-(furan-2-carbonyl)hydrazine-1-carboxylate **(9).**

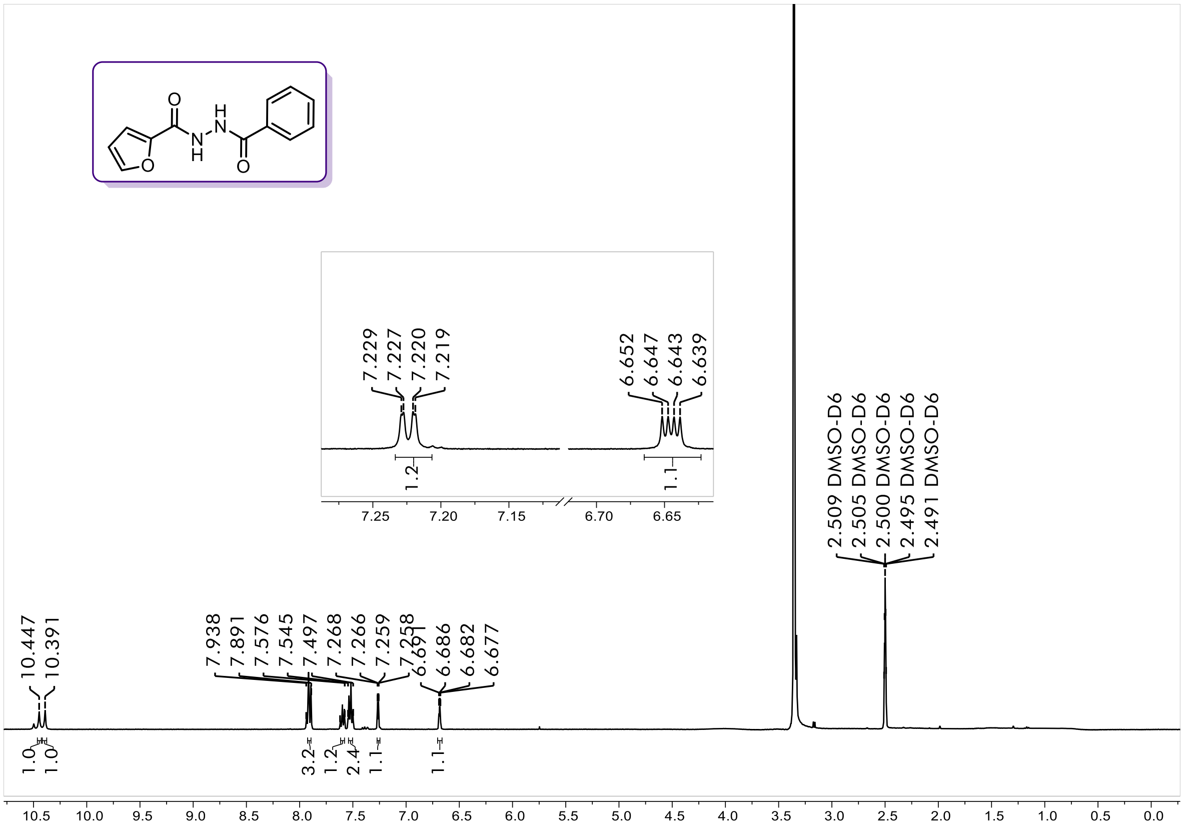

Spectrum 4. ^{1}H-NMR of *N*'-benzoylfuran-2-carbohydrazide **(4a).**

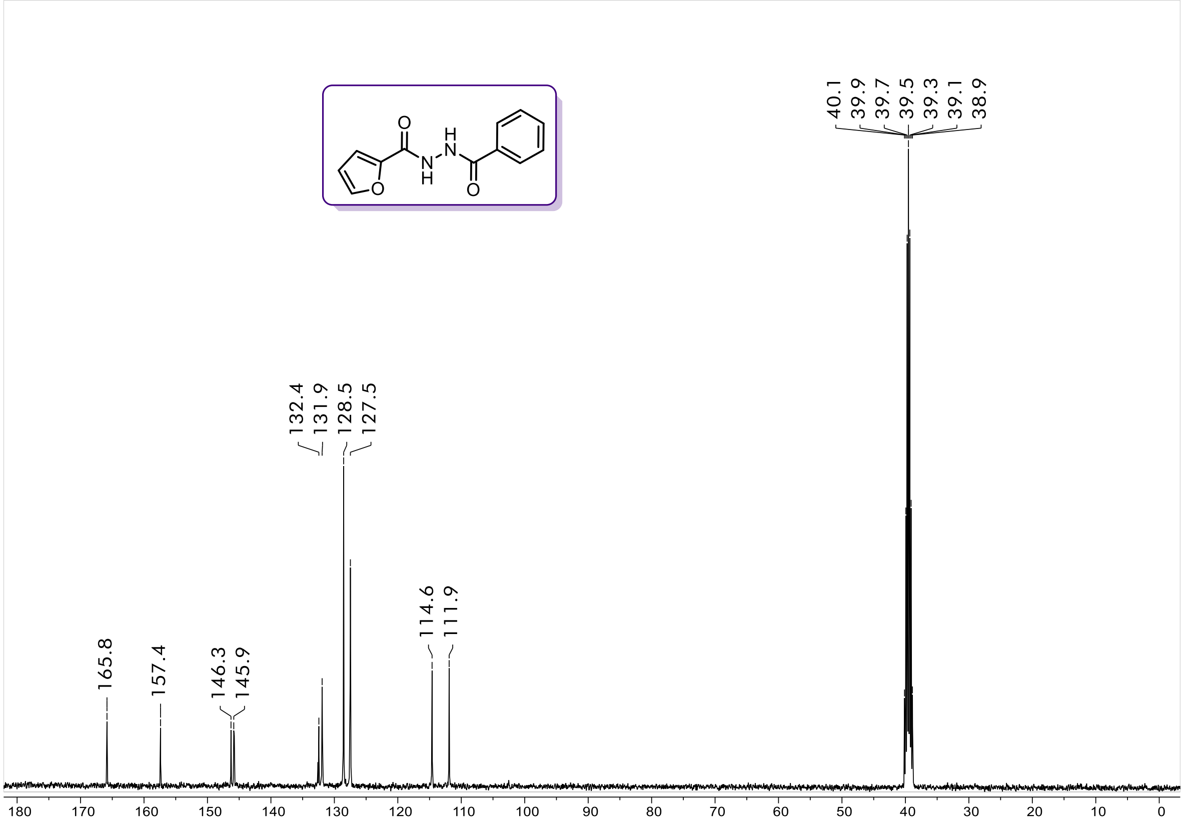

Spectrum 5. ^{13}C-NMR of *N*'-benzoylfuran-2-carbohydrazide **(4a).**

Description:
Ionization Mode:ESI+
History:Determine m/z[Peak Detect[Centroid,30,Area];Correct Base[];Smooth[5]];Correct Base[5.0%];Average(MS[...

Mass Calibration data:Cal_PEG_600
Created:8/18/2023 11:30:28 AM
Created by:AccuTOF

Charge number:1
Element:^{12}C:0 .. 12, ^{1}H:0 .. 11, ^{14}N:0 .. 2, ^{16}O:3 .. 3

Tolerance:30.00(ppm), 5.00 .. 15.00(mmu)

Unsaturation Number:-1.0 .. 50.0 (Fraction:Both)

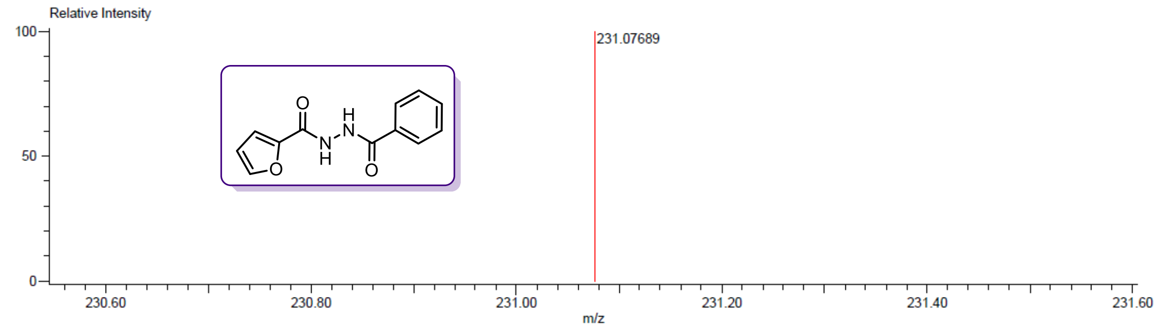

Mass	Intensity	Calc. Mass	Mass Difference (mmu)	Mass Difference (ppm)	Possible Formula	Unsaturation Number
231.07689	3886789.45	231.07697	-0.07	-0.32	$^{12}C_{12}{}^{1}H_{11}{}^{14}N_2{}^{16}O_3$	8.5

Spectrum 6. HRMS of *N*'-benzoylfuran-2-carbohydrazide **(4a).**

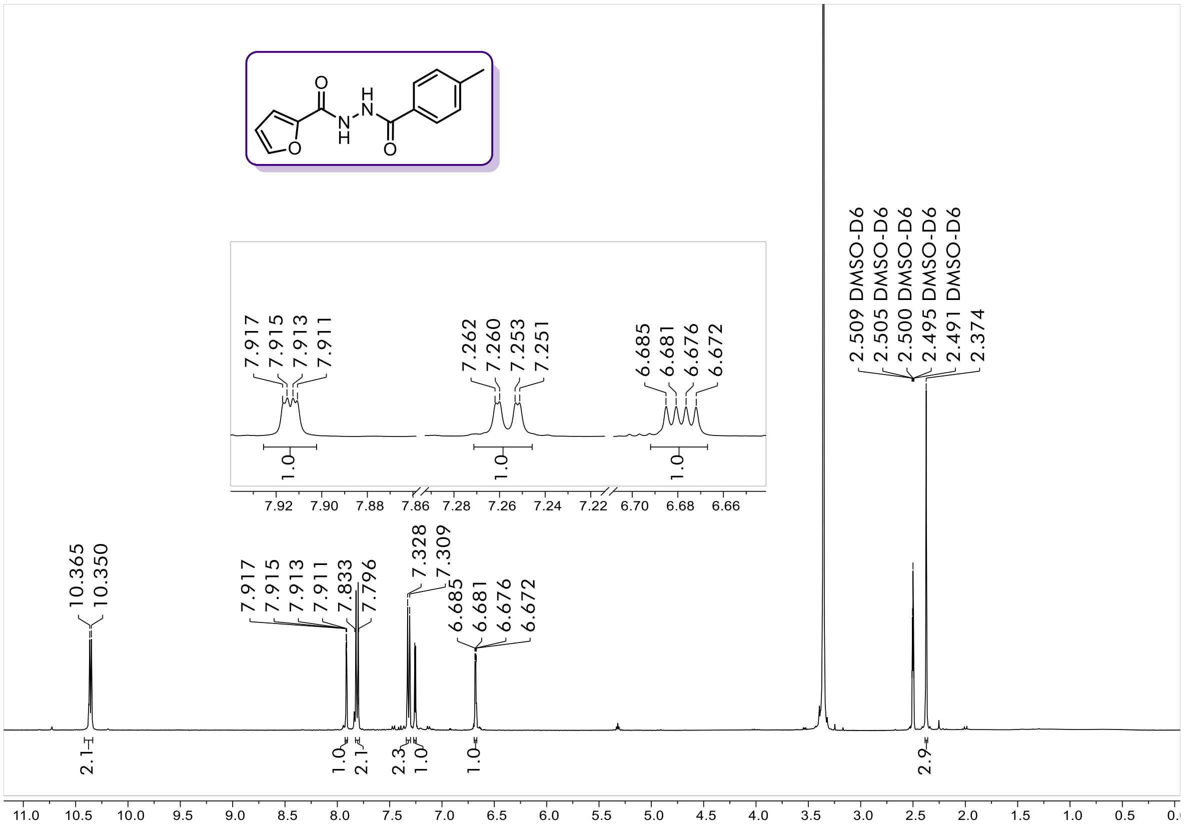

Spectrum 7. ^{1}H-NMR of *N*'-(4-methylbenzoyl)furan-2-carbohydrazide (**4b**).

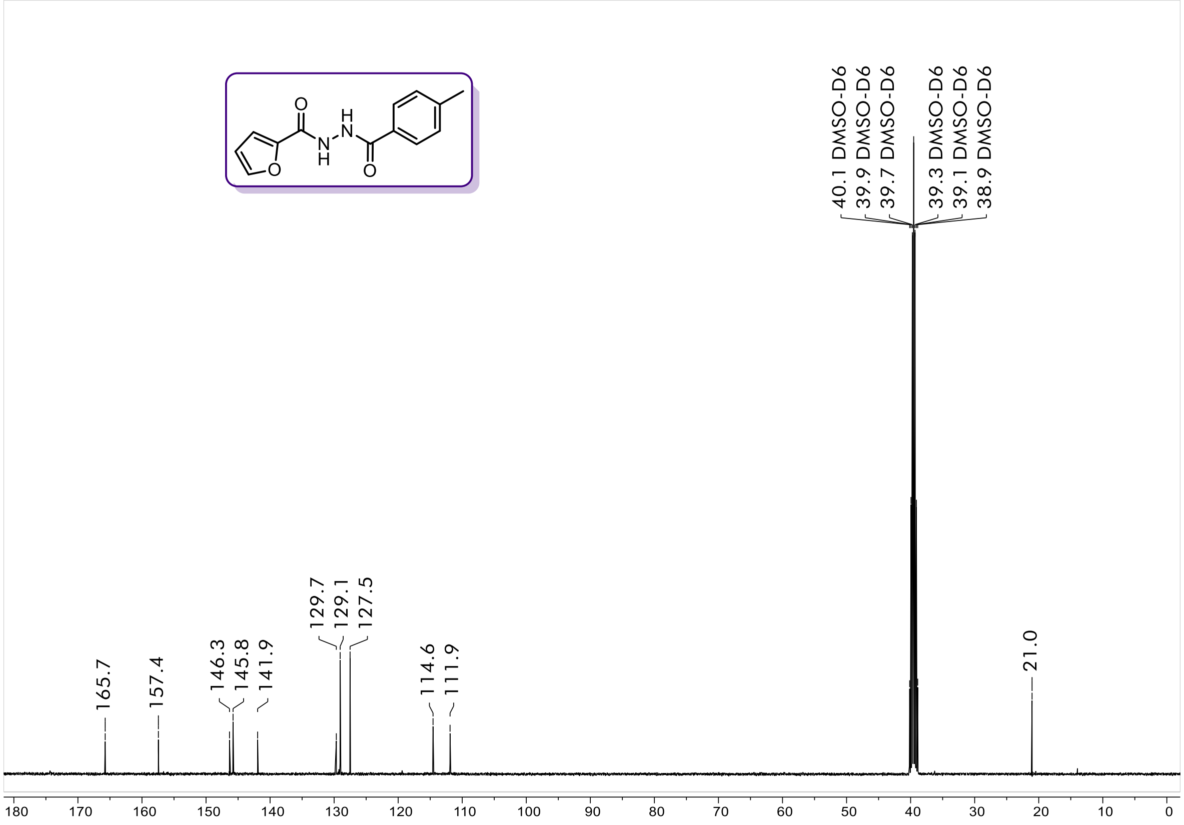

Spectrum 8. ^{13}C-NMR of *N*'-(4-methylbenzoyl)furan-2-carbohydrazide (**4b**).

Description:
Ionization Mode:ESI+
History:Determine m/z[Peak Detect[Centroid,30,Area];Correct Base[];Smooth[5]];Correct Base[5.0%];Average(MS[...

Mass Calibration data:Cal_PEG_600
Created:9/14/2023 1:34:27 PM
Created by:AccuTOF

Charge number:1
Element:^{12}C:0 .. 13, ^{1}H:0 .. 13, ^{14}N:0 .. 2, ^{16}O:0 .. 3

Tolerance:100.00(ppm), 5.00 .. 15.00(mmu)

Unsaturation Number:-1.0 .. 50.0 (Fraction:Both)

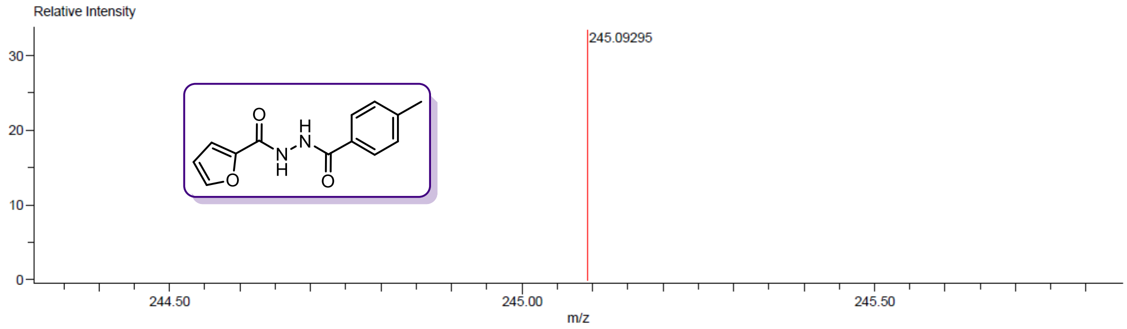

Mass	Intensity	Calc. Mass	Mass Difference (mmu)	Mass Difference (ppm)	Possible Formula	Unsaturation Number
245.09295	22587.03	245.09262	0.33	1.36	$^{12}C_{13}{}^{1}H_{13}{}^{14}N_{2}{}^{16}O_{3}$	8.5

Spectrum 9. HRMS of *N*'-(4-methylbenzoyl)furan-2-carbohydrazide (**4b**).

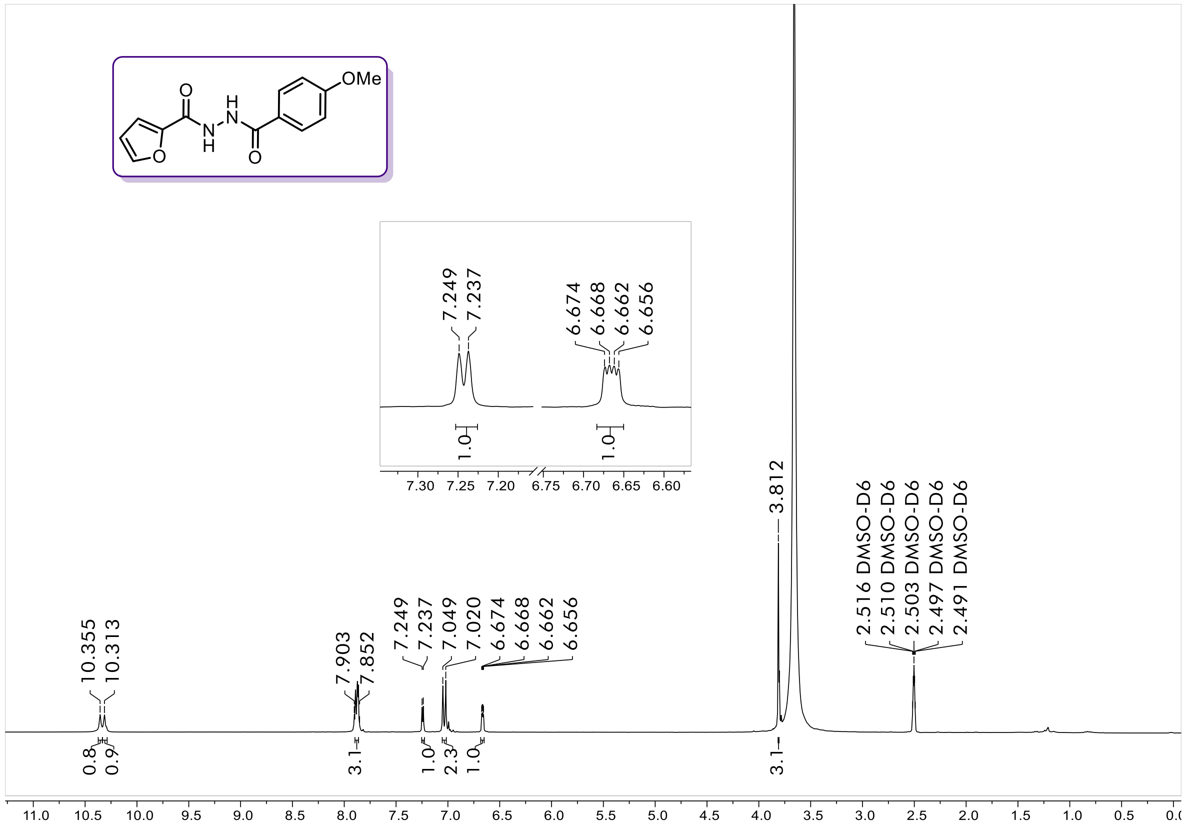

Spectrum 10. ^{1}H-NMR of *N*'-(4-methoxybenzoyl)furan-2-carbohydrazide (**4c**).

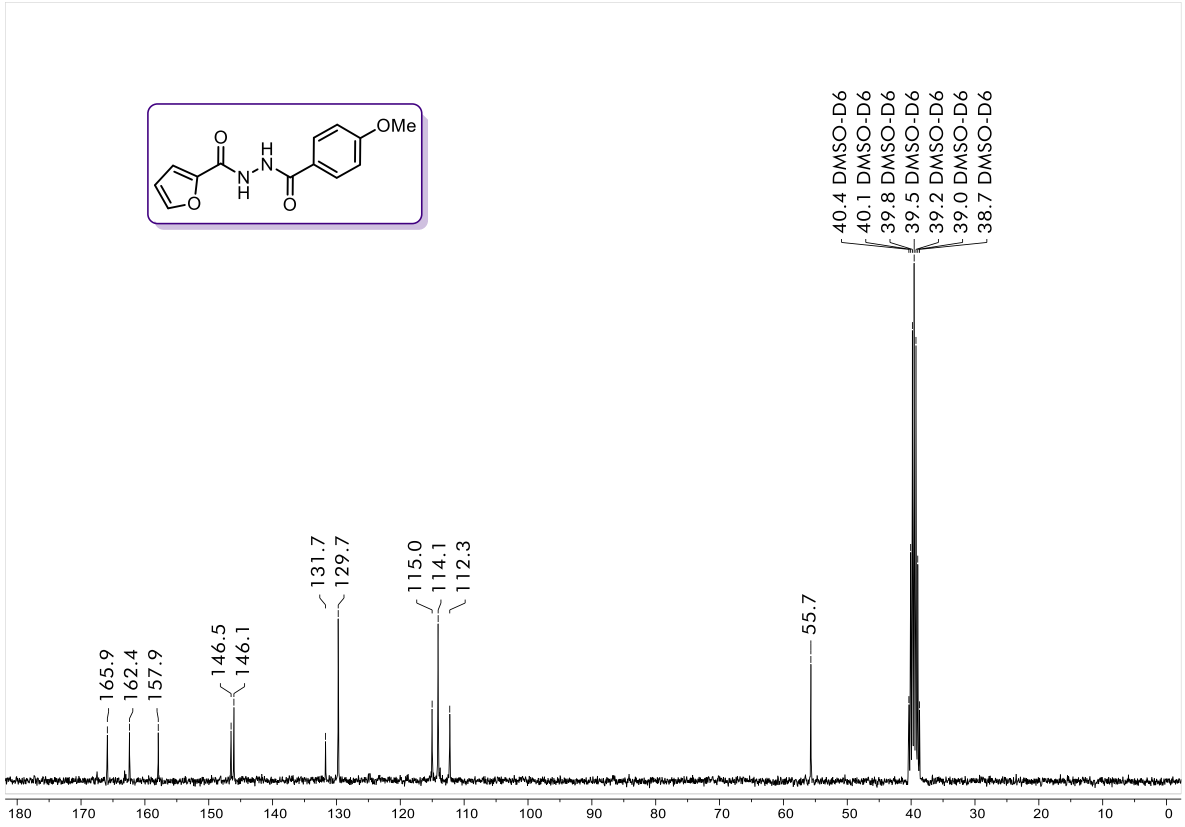

Spectrum 11. ^{13}C-NMR of *N*'-(4-methoxybenzoyl)furan-2-carbohydrazide (**4c**).

Description:
Ionization Mode:ESI+
History:Determine m/z[Peak Detect[Centroid,30,Area];Correct Base[];Smooth[5]];Correct Base[5.0%];Average(MS[...

Mass Calibration data:Cal_PEG_600
Created:10/31/2022 1:56:35 PM
Created by:AccuTOF

Charge number:1
Element:^{12}C:0 .. 13, ^{1}H:0 .. 50, ^{127}I:0 .. 0, ^{14}N:2 .. 2, ^{16}O:0 .. 4

Tolerance:6.00(ppm), 5.00 .. 15.00(mmu)

Unsaturation Number:0.0 .. 50.0 (Fraction:Both)

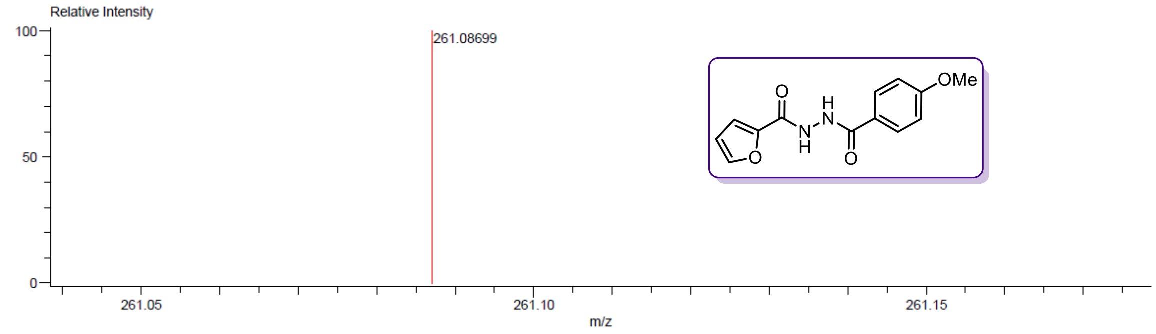

Mass	Intensity	Calc. Mass	Mass Difference (mmu)	Mass Difference (ppm)	Possible Formula	Unsaturation Number
261.08699	2306.71	261.08753	-0.54	-2.06	$^{12}C_{13}{}^{1}H_{13}{}^{14}N_2{}^{16}O_4$	8.5

Spectrum 12. HRMS of *N*'-(4-methoxybenzoyl)furan-2-carbohydrazide (**4c**).

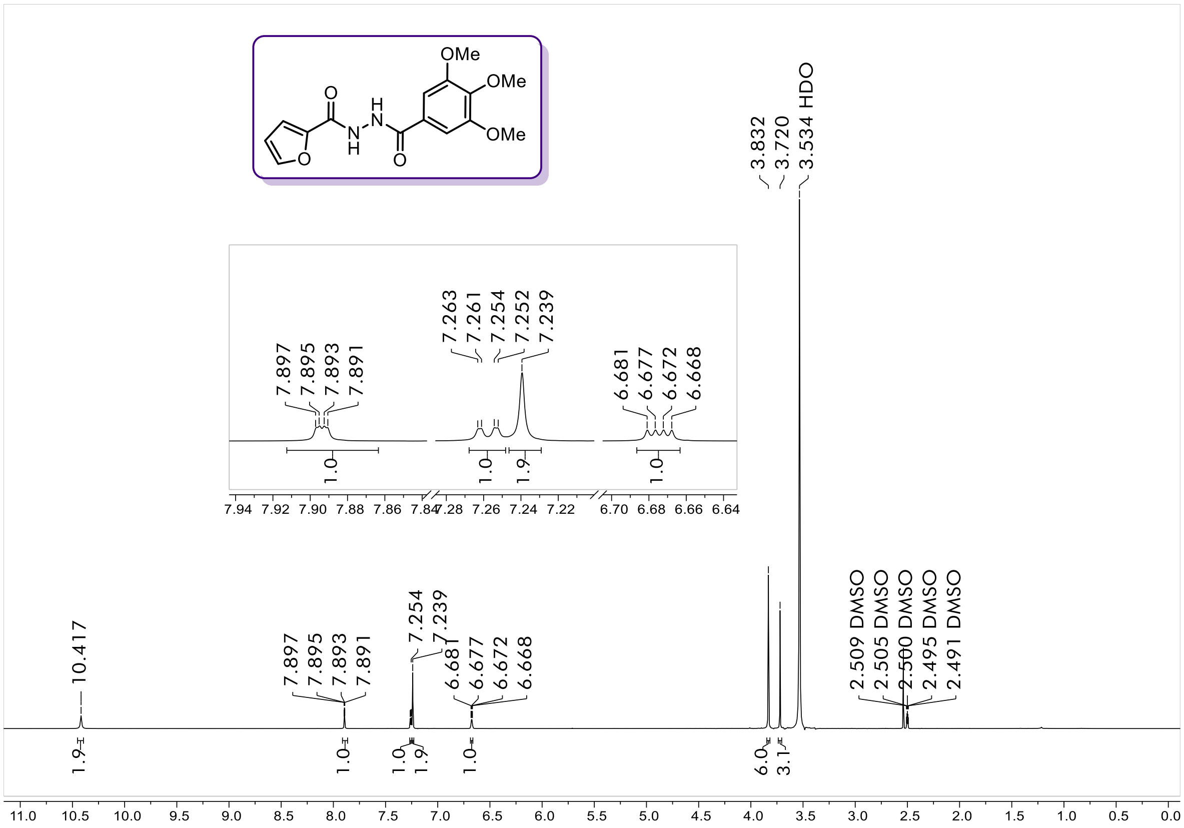

Spectrum 13. ^{1}H-NMR of N'-(3,4,5-trimethoxybenzoyl)furan-2-carbohydrazide (**4d**).

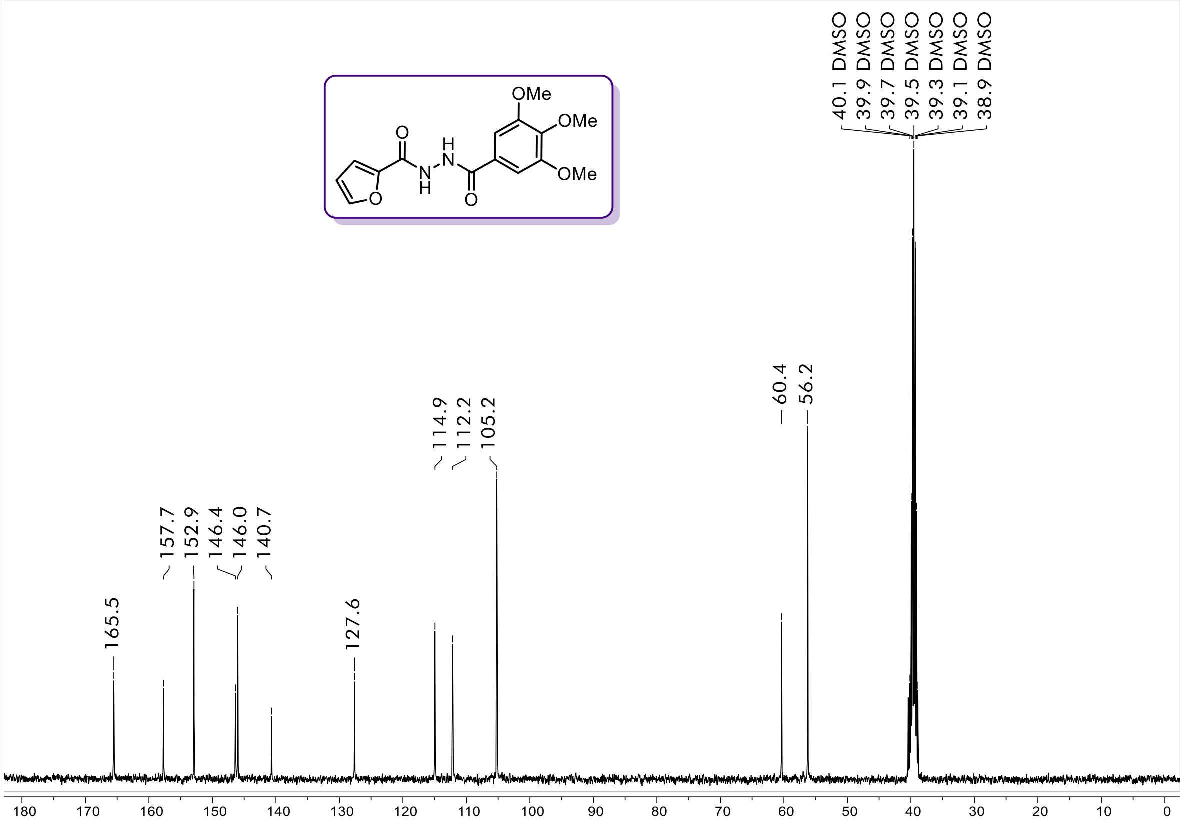

Spectrum 14. ^{13}C-NMR of N'-(3,4,5-trimethoxybenzoyl)furan-2-carbohydrazide (**4d**).

Description:
Ionization Mode:ESI+
History:Determine m/z[Peak Detect[Centroid,30,Area];Correct Base[];Smooth[5]];Correct Base[5.0%];Average(MS[...

Mass Calibration data:Cal_PEG_600
Created:1/16/2023 3:34:15 PM
Created by:AccuTOF

Charge number:1 Tolerance:5.00(ppm), 5.00 .. 15.00(mmu) Unsaturation Number:0.0 .. 100.0 (Fraction:Both)

Element:^{12}C:0 .. 15, ^{1}H:0 .. 50, ^{14}N:0 .. 2, ^{16}O:0 .. 6, ^{31}P:0 .. 0

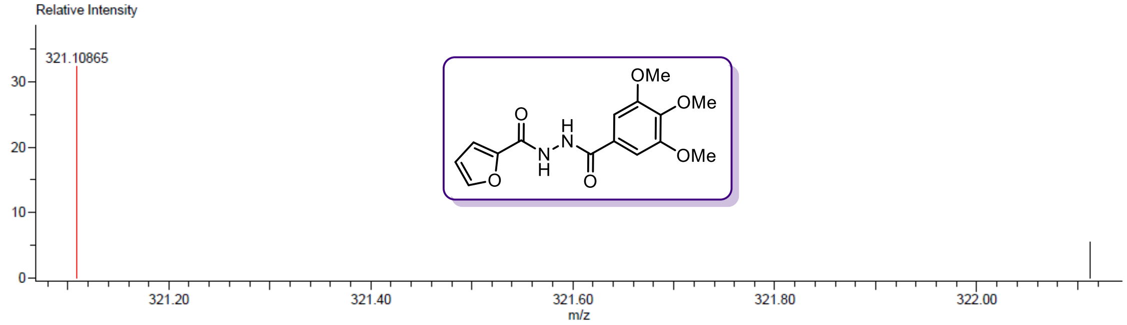

Mass	Intensity	Calc. Mass	Mass Difference (mmu)	Mass Difference (ppm)	Possible Formula	Unsaturation Number
321.10865	352159.04	321.10866	-0.01	-0.03	$^{12}C_{15}{}^{1}H_{17}{}^{14}N_2{}^{16}O_6$	8.5

Spectrum 15. HRMS of N'-(3,4,5-trimethoxybenzoyl)furan-2-carbohydrazide (**4d**).

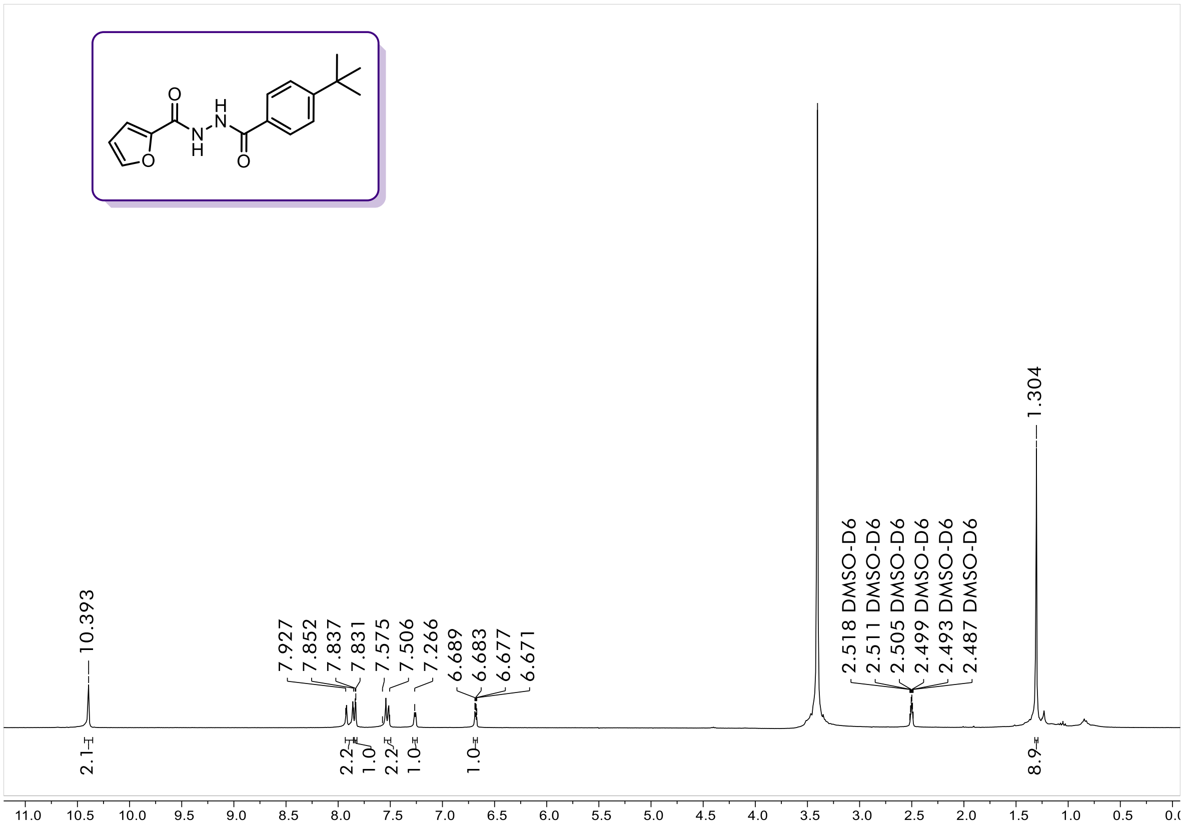

Spectrum 16. ^{1}H-NMR of N'-(4-(*tert*-butyl)benzoyl)furan-2-carbohydrazide (**4e**).

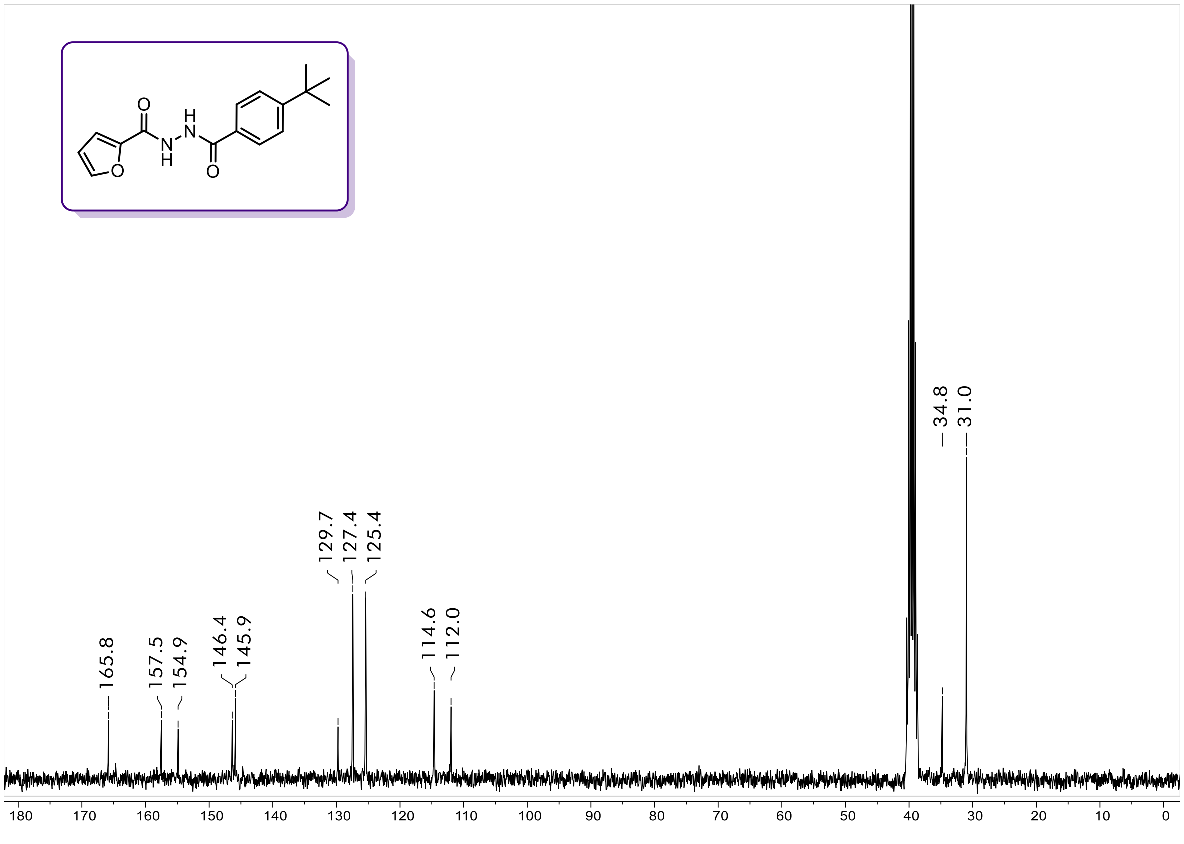

Spectrum 17. ^{13}C-NMR of N'-(4-(*tert*-butyl)benzoyl)furan-2-carbohydrazide (**4e**).

Description:
Ionization Mode:ESI+
History:Determine m/z[Peak Detect[Centroid,30,Area];Correct Base[];Smooth[5]];Correct Base[5.0%];Average(MS[...

Mass Calibration data:Cal_PEG_600
Created:11/22/2023 11:28:13 AM
Created by:AccuTOF

Charge number:1
Element:^{12}C:0 .. 16, ^{1}H:0 .. 20, ^{14}N:2 .. 2, ^{16}O:3 .. 3

Tolerance:50.00(ppm), 5.00 .. 15.00(mmu)

Unsaturation Number:-1.0 .. 70.0 (Fraction:Both)

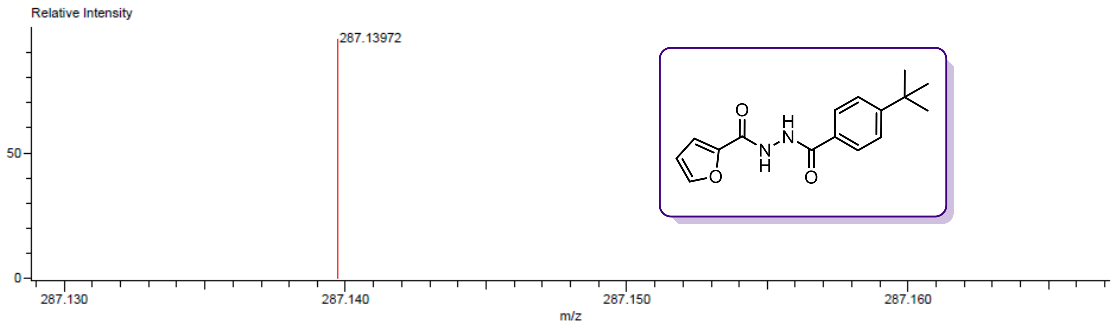

Mass	Intensity	Calc. Mass	Mass Difference (mmu)	Mass Difference (ppm)	Possible Formula	Unsaturation Number
287.13972	18963.12	287.13957	0.15	0.52	$^{12}C_{16}{}^{1}H_{19}{}^{14}N_2{}^{16}O_3$	8.5

Spectrum 18. HRMS of N'-(4-(*tert*-butyl)benzoyl)furan-2-carbohydrazide (**4e**).

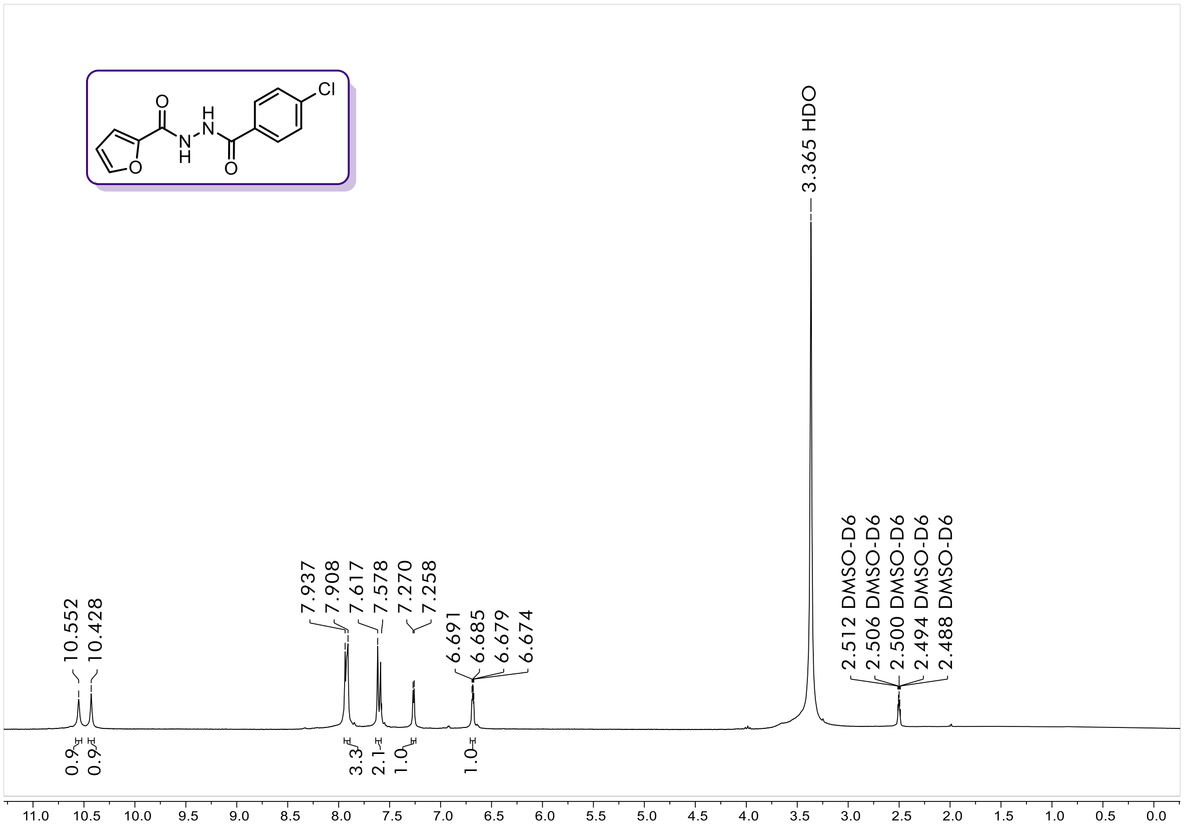

Spectrum 19. ^{1}H-NMR of N'-(4-chlorobenzoyl)furan-2-carbohydrazide (**4f**).

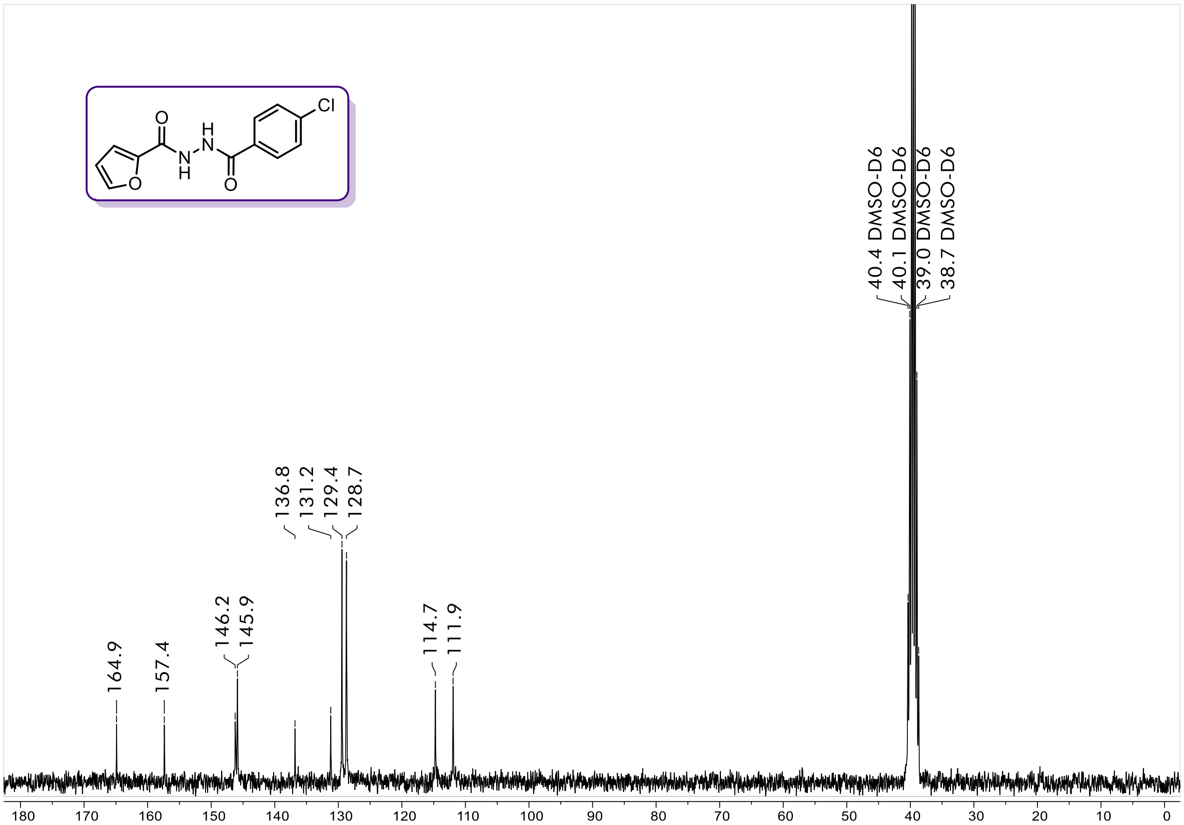

Spectrum 20. ^{13}C-NMR of N'-(4-chlorobenzoyl)furan-2-carbohydrazide (**4f**).

Description:
Ionization Mode:ESI+
History:Determine m/z[Peak Detect[Centroid,30,Area];Correct Base[];Smooth[5]];Correct Base[5.0%];Average(MS[...

Mass Calibration data:Cal_PEG_600
Created:9/19/2023 2:17:35 PM
Created by:AccuTOF

Charge number:1
Tolerance:100.00(ppm), 5.00 .. 15.00(mmu)
Unsaturation Number:-1.0 .. 50.0 (Fraction:Both)
Element:^{12}C:0 .. 12, ^{1}H:0 .. 20, ^{35}Cl:0 .. 1, ^{14}N:0 .. 2, ^{16}O:0 .. 3

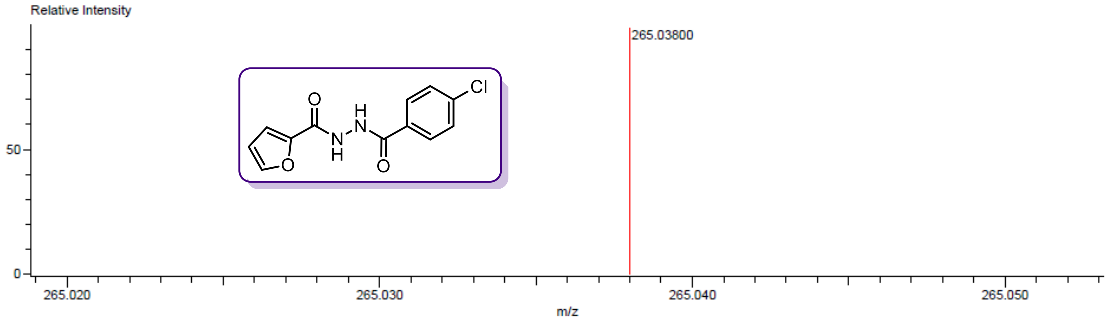

Mass	Intensity	Calc. Mass	Mass Difference (mmu)	Mass Difference (ppm)	Possible Formula	Unsaturation Number
265.03800	3911.69	265.03799	0.01	0.03	$^{12}C_{12}{}^{1}H_{10}{}^{35}Cl_{1}{}^{14}N_{2}{}^{16}O_{3}$	8.5

Spectrum 21. HRMS of N'-(4-chlorobenzoyl)furan-2-carbohydrazide (**4f**).

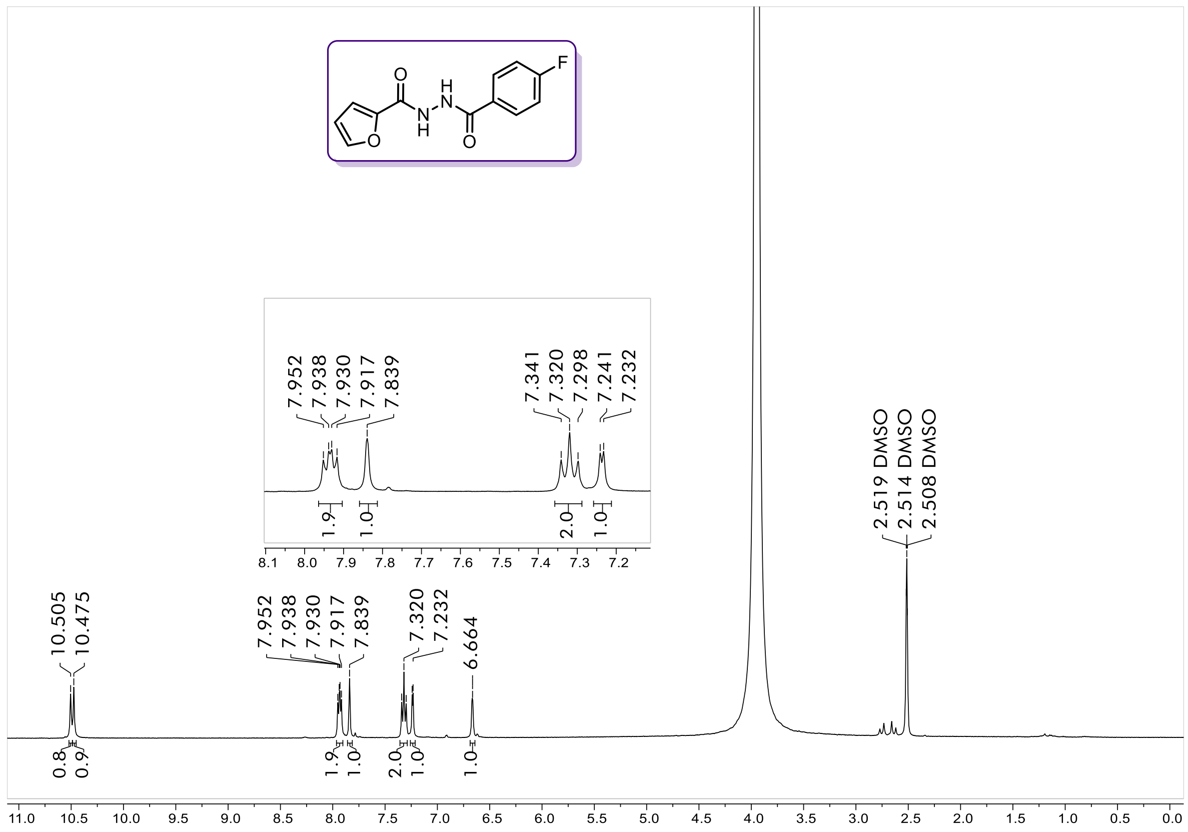

Spectrum 22. ^{1}H-NMR of *N*'-(4-fluorobenzoyl)furan-2-carbohydrazide (**4g**).

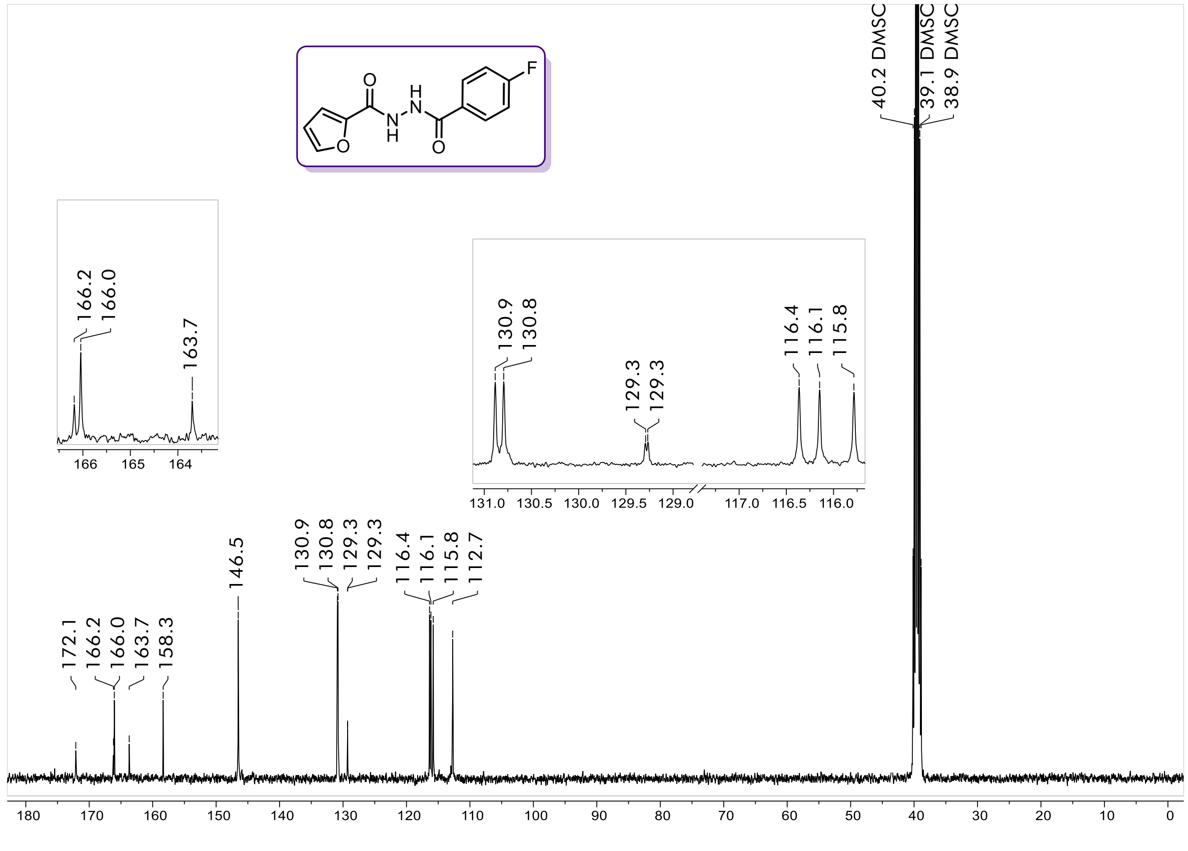

Spectrum 23. ^{13}C-NMR of *N*'-(4-fluorobenzoyl)furan-2-carbohydrazide (**4g**).

Description:
Ionization Mode:ESI+
History:Determine m/z[Peak Detect[Centroid,30,Area];Correct Base[];Smooth[5]];Correct Base[5.0%];Average(MS[...

Mass Calibration data:Cal_PEG_600
Created:10/31/2022 2:09:43 PM
Created by:AccuTOF

Charge number:1
Tolerance:6.00(ppm), 5.00 .. 15.00(mmu)
Unsaturation Number:0.0 .. 50.0 (Fraction:Both)
Element:^{12}C:0 .. 12, ^{1}H:0 .. 50, ^{19}F:0 .. 1, ^{127}I:0 .. 0, ^{14}N:2 .. 2, ^{16}O:0 .. 3

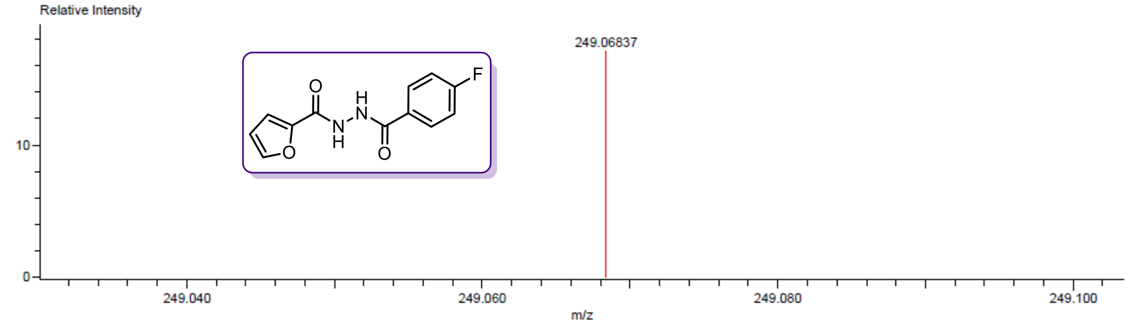

Mass	Intensity	Calc. Mass	Mass Difference (mmu)	Mass Difference (ppm)	Possible Formula	Unsaturation Number
249.06837	1384.41	249.06755	0.82	3.31	$^{12}C_{12}{}^{1}H_{10}{}^{19}F_{1}{}^{14}N_{2}{}^{16}O_{3}$	8.5

Spectrum 24. HRMS of *N*'-(4-fluorobenzoyl)furan-2-carbohydrazide (**4g**).

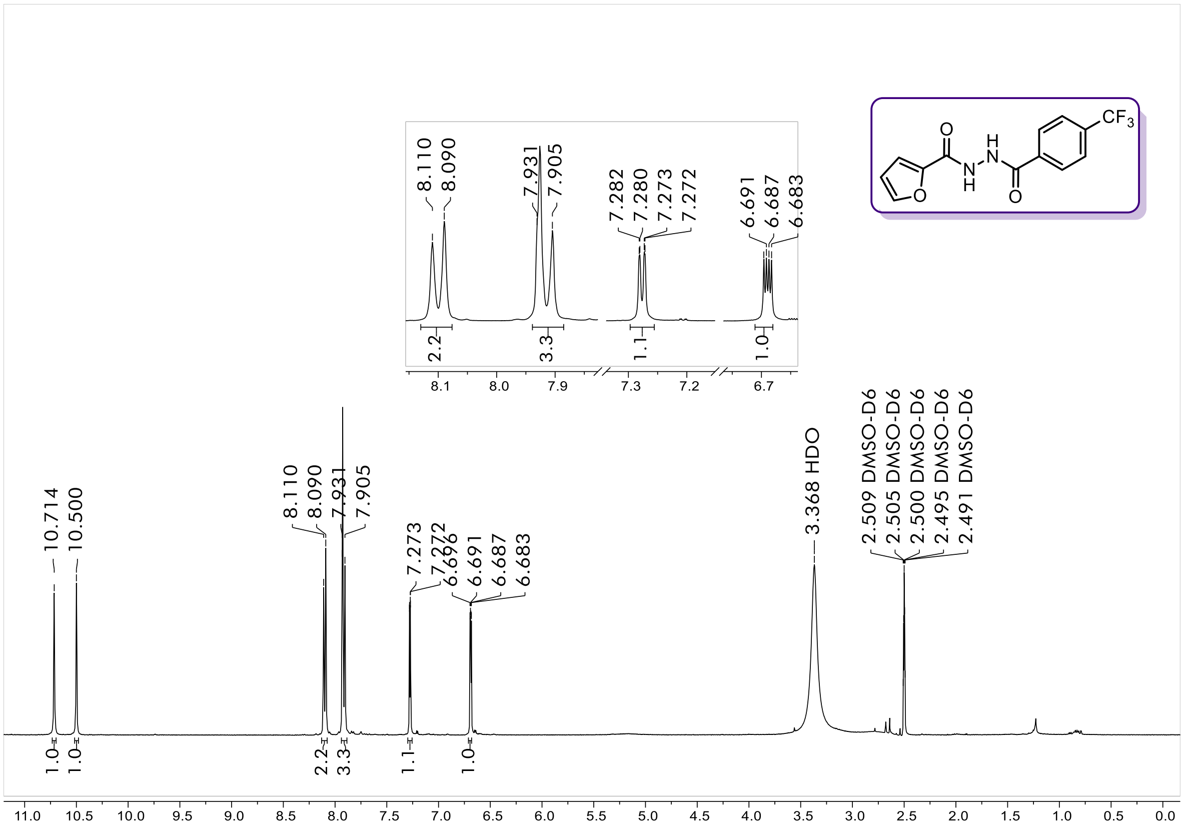

Spectrum 25. ^{1}H-NMR of *N*'-(4-(trifluoromethyl)benzoyl)furan-2-carbohydrazide (**4h**).

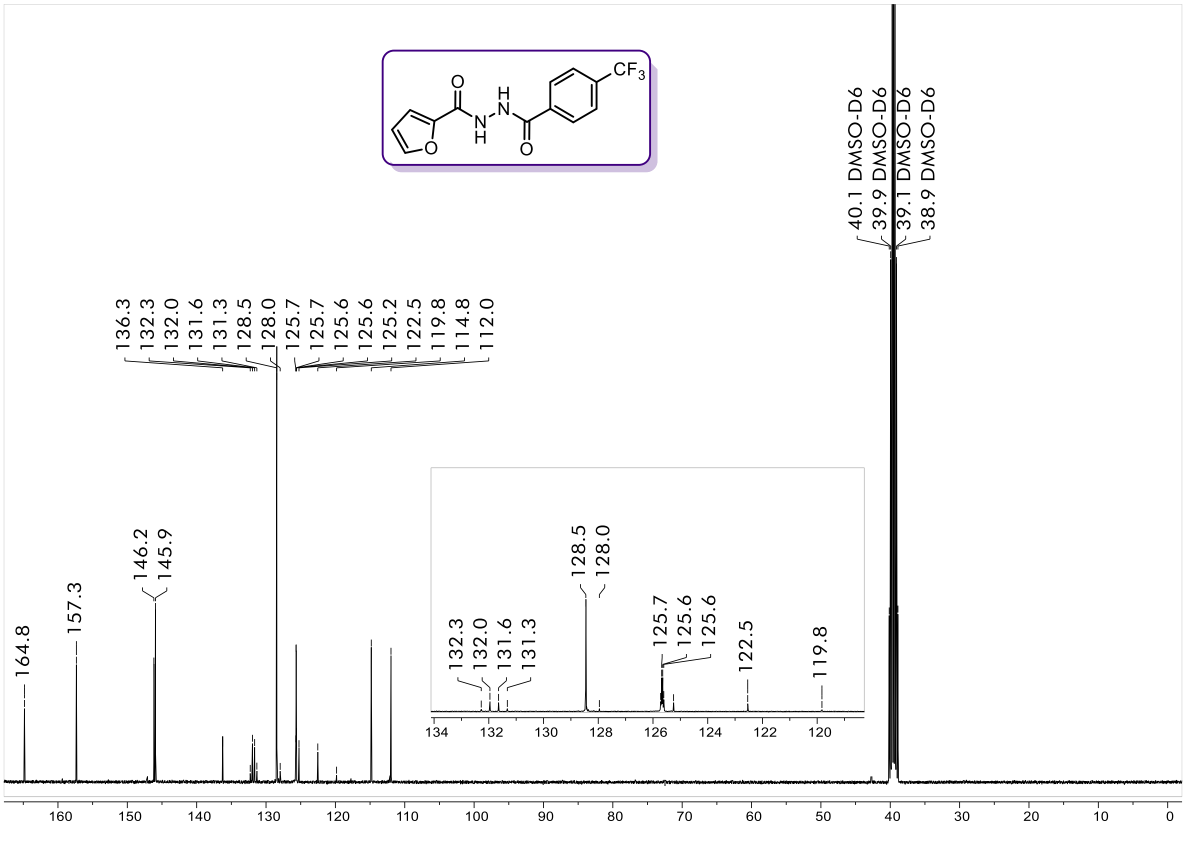

Spectrum 26. ^{13}C-NMR of *N*'-(4-(trifluoromethyl)benzoyl)furan-2-carbohydrazide (**4h**).

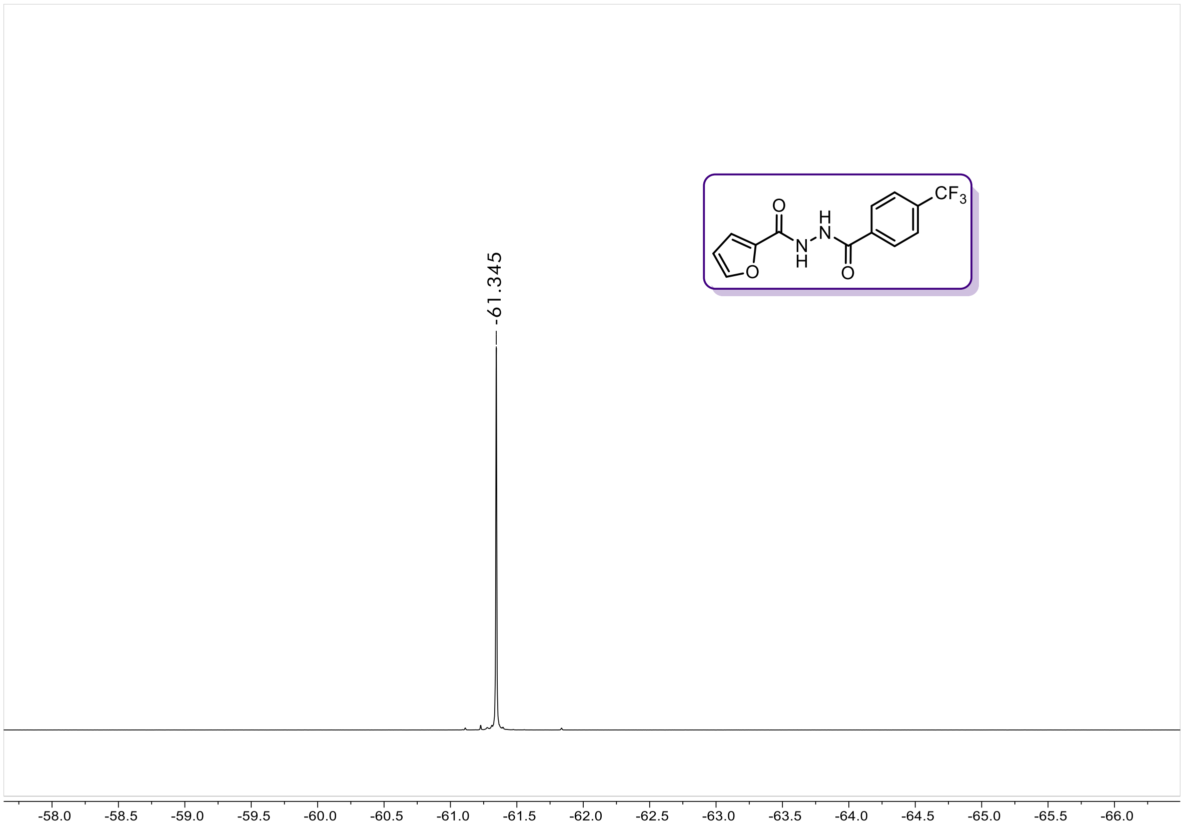

Spectrum 27. ^{19}F-NMR of *N*'-(4-(trifluoromethyl)benzoyl)furan-2-carbohydrazide (**4h**).

Description:
Ionization Mode:ESI+
History:Determine m/z[Peak Detect[Centroid,30,Area];Correct Base[];Smooth[5]];Correct Base[5.0%];Average(MS[...

Mass Calibration data:Cal_PEG_600
Created:11/24/2022 2:51:53 PM
Created by:AccuTOF

Charge number:1
Tolerance:2000.00(ppm), 5.00 .. 15.00(mmu)
Unsaturation Number:0.0 .. 24.0 (Fraction:Both)
Element:^{12}C:0 .. 13, ^{1}H:0 .. 60, ^{79}Br:0 .. 0, ^{19}F:3 .. 3, ^{14}N:2 .. 2, ^{16}O:2 .. 3

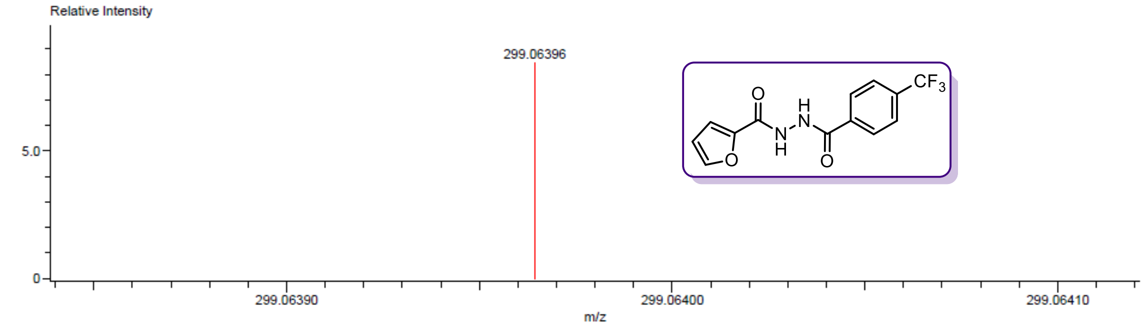

Mass	Intensity	Calc. Mass	Mass Difference (mmu)	Mass Difference (ppm)	Possible Formula	Unsaturation Number
299.06396	1639.35	299.06435	-0.39	-1.29	$^{12}C_{13}{}^{1}H_{10}{}^{19}F_{3}{}^{14}N_{2}{}^{16}O_{3}$	8.5

Spectrum 28. HRMS of *N*'-(4-(trifluoromethyl)benzoyl)furan-2-carbohydrazide (**4h**).

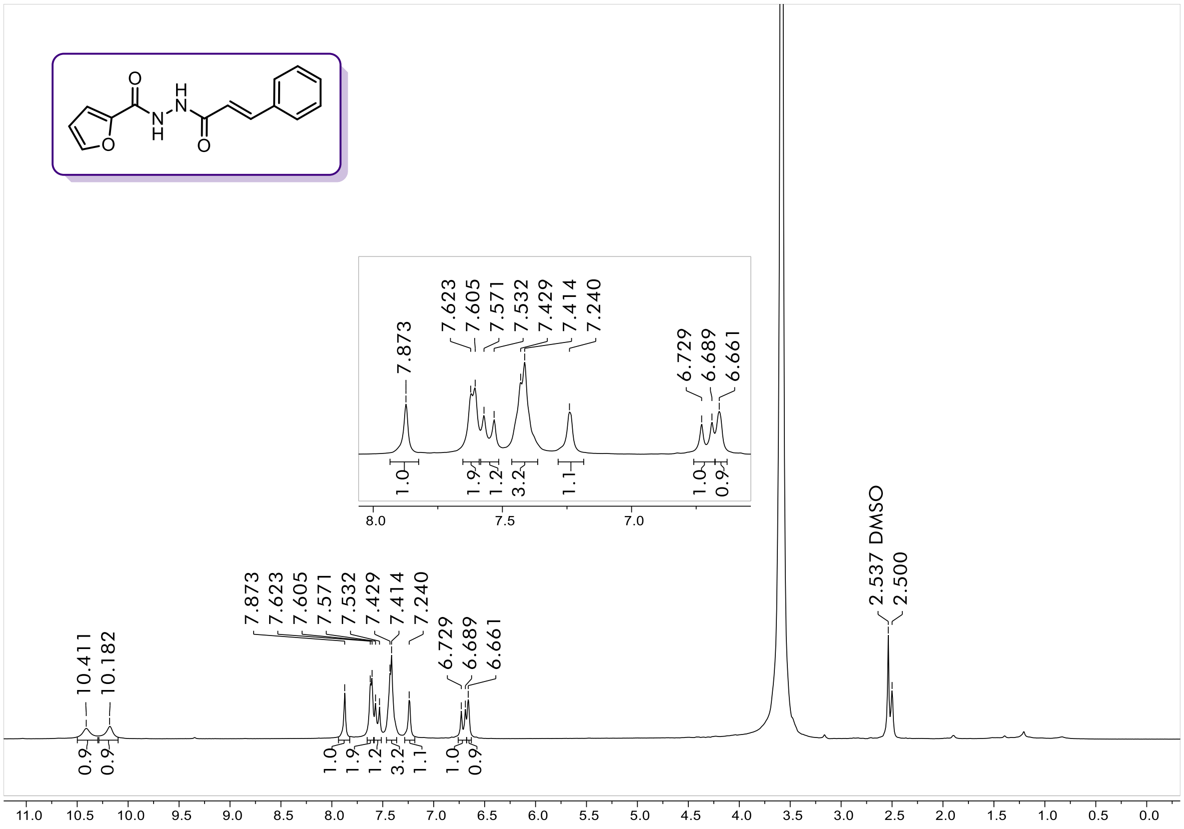

Spectrum 29. ^{1}H-NMR of *N*'-cinnamoylfuran-2-carbohydrazide (**4i**).

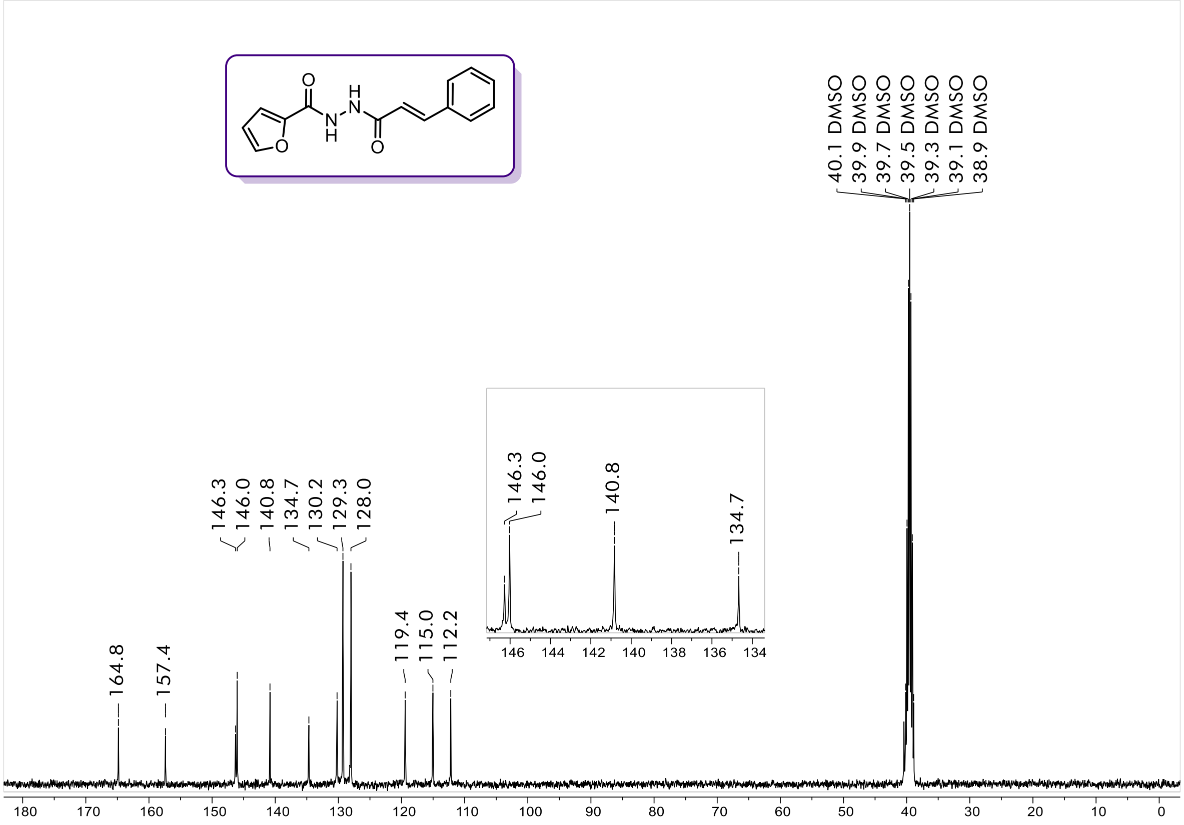

Spectrum 30. ^{13}C-NMR of *N*'-cinnamoylfuran-2-carbohydrazide (**4i**).

Description:
Ionization Mode:ESI+
History:Determine m/z[Peak Detect[Centroid,30,Area];Correct Base[];Smooth[5]];Correct Base[5.0%];Average(MS[...

Mass Calibration data:Cal_PEG_600
Created:1/16/2023 3:26:52 PM
Created by:AccuTOF

Charge number:1
Tolerance:5.00(ppm), 5.00 .. 15.00(mmu)
Unsaturation Number:0.0 .. 100.0 (Fraction:Both)
Element:^{12}C:0 .. 14, ^{1}H:0 .. 50, ^{14}N:0 .. 2, ^{16}O:0 .. 3, ^{31}P:0 .. 0

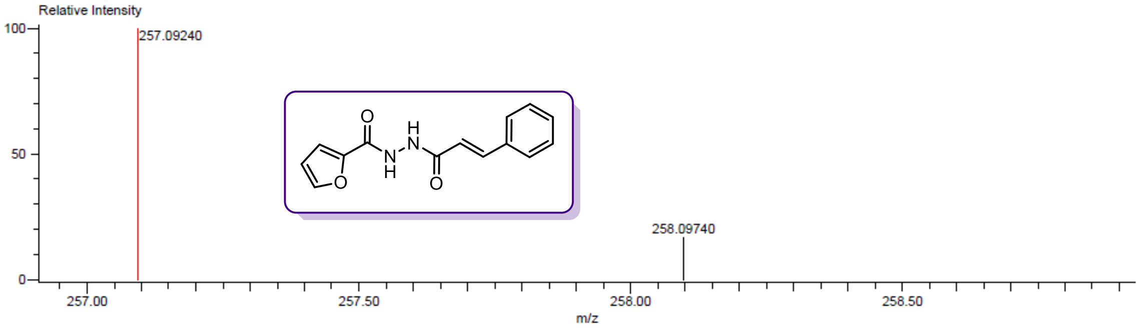

Mass	Intensity	Calc. Mass	Mass Difference (mmu)	Mass Difference (ppm)	Possible Formula	Unsaturation Number
257.09240	23939.19	257.09262	-0.22	-0.86	$^{12}C_{14}{}^{1}H_{13}{}^{14}N_{2}{}^{16}O_{3}$	9.5

Spectrum 31. HRMS of *N*'-cinnamoylfuran-2-carbohydrazide (**4i**).

NMR SPECTRA OF DIAMIDES 5a-f

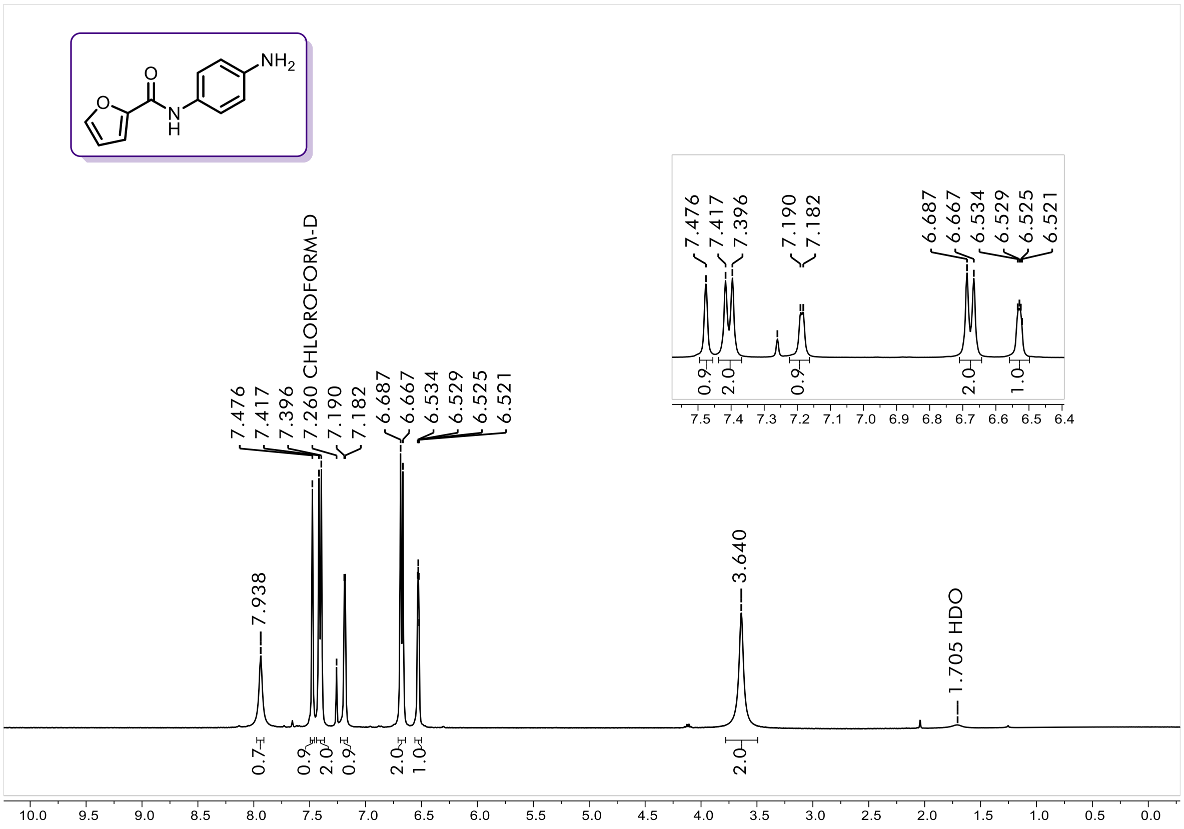

Spectrum 32. ^{1}H-NMR of *N*-(4-aminophenyl)furan-2-carboxamide **(10).**

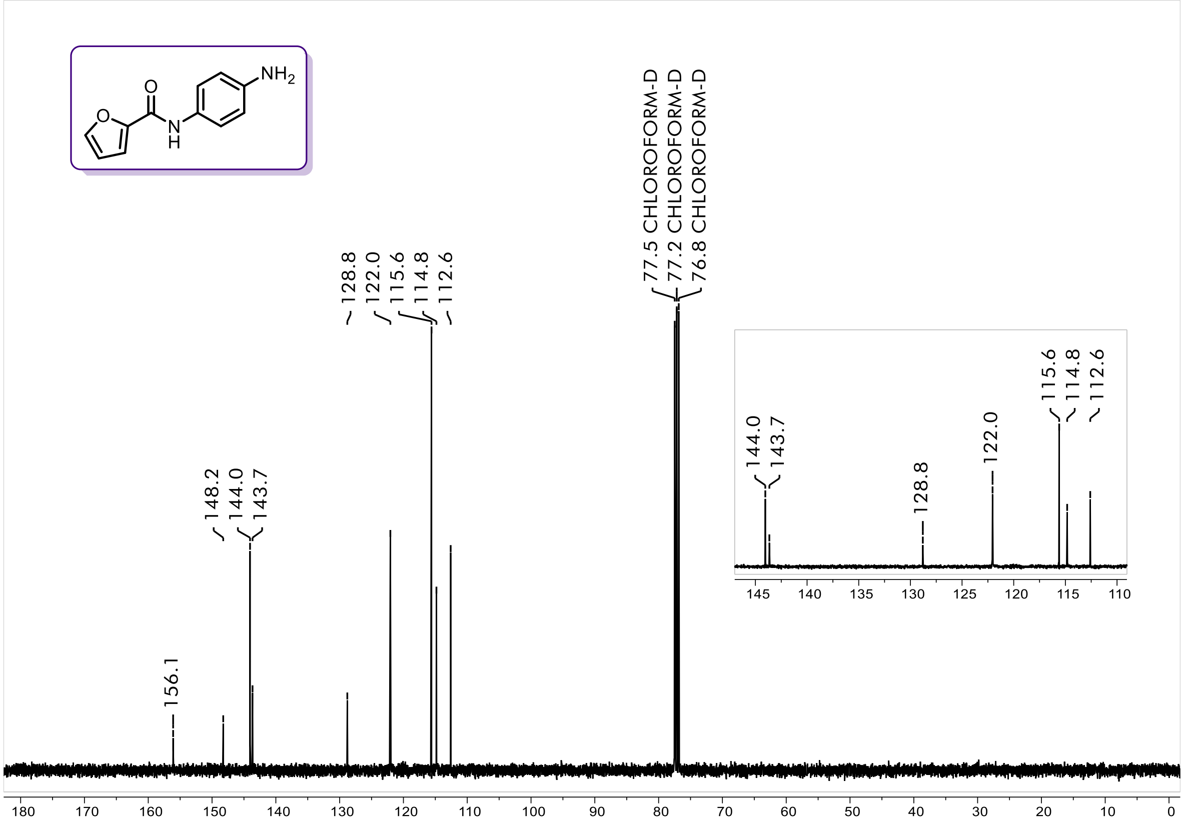

Spectrum 33. ^{13}C-NMR of *N*-(4-aminophenyl)furan-2-carboxamide **(10).**

Description:
Ionization Mode:ESI+
History:Determine m/z[Peak Detect[Centroid,30,Area];Correct Base[];Smooth[5]];Correct Base[5.0%];Average(MS[...

Mass Calibration data:Cal_PEG_600
Created:3/12/2024 10:18:22 AM
Created by:AccuTOF

Charge number:1 Tolerance:3.00(ppm), 5.00 .. 15.00(mmu) Unsaturation Number:-1.0 .. 100.0 (Fraction:Both)
Element:^{12}C:11 .. 11, ^{1}H:0 .. 20, ^{14}N:2 .. 2, ^{16}O:2 .. 2, ^{32}S:0 .. 0

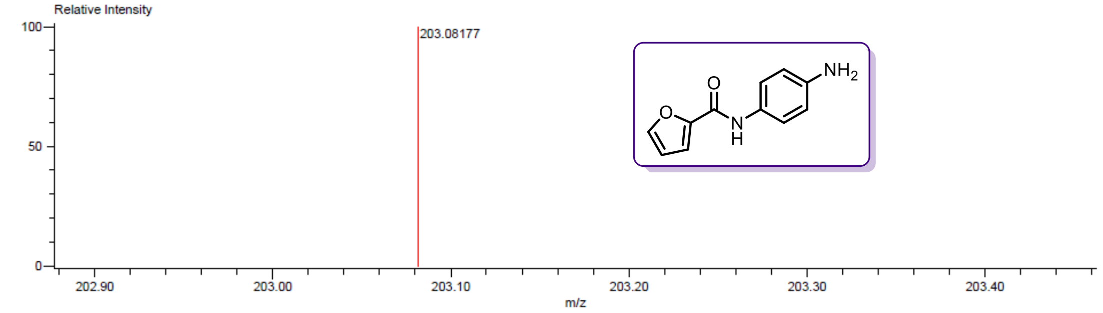

Mass	Intensity	Calc. Mass	Mass Difference (mmu)	Mass Difference (ppm)	Possible Formula	Unsaturation Number
203.08177	20319.68	203.08205	-0.28	-1.39	$^{12}C_{11}{}^{1}H_{11}{}^{14}N_2{}^{16}O_2$	7.5

Spectrum 34. HRMS of *N*-(4-aminophenyl)furan-2-carboxamide **(10).**

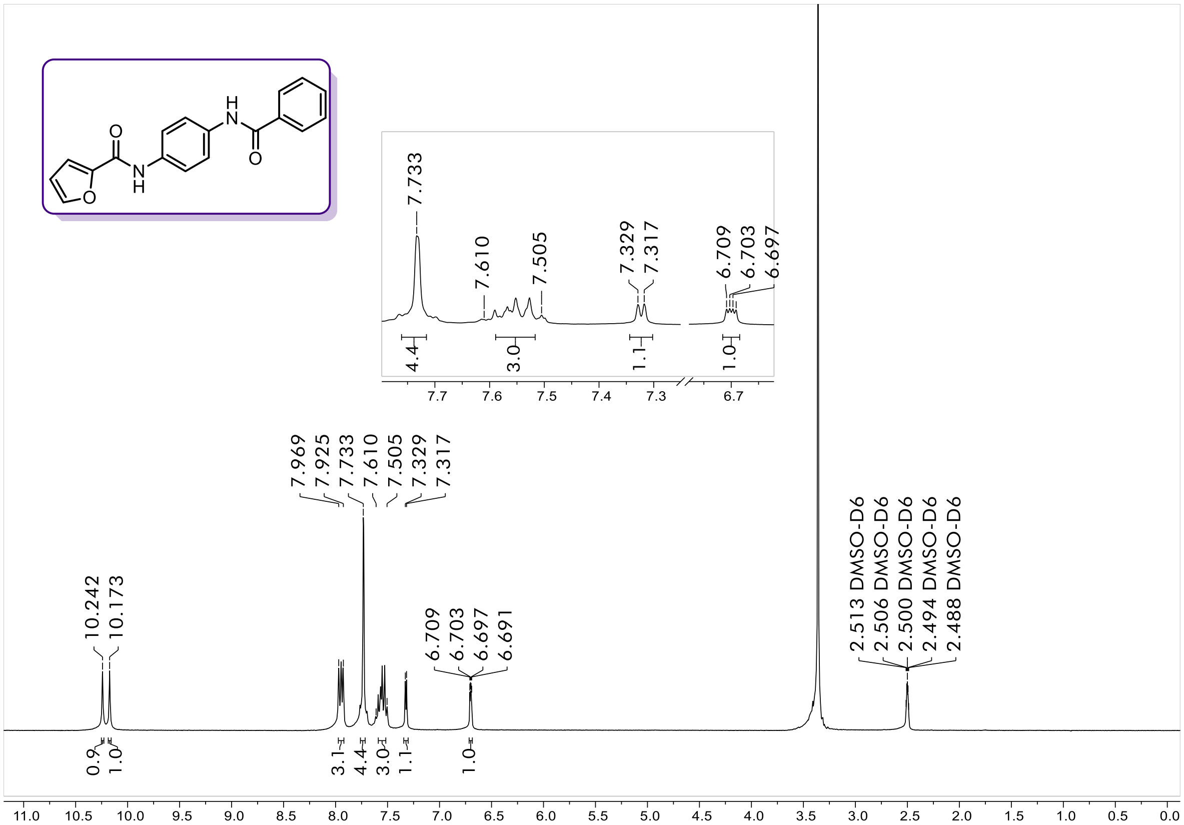

Spectrum 35. ^{1}H-NMR of *N*-(4-benzamidophenyl)furan-2-carboxamide **(5a).**

.

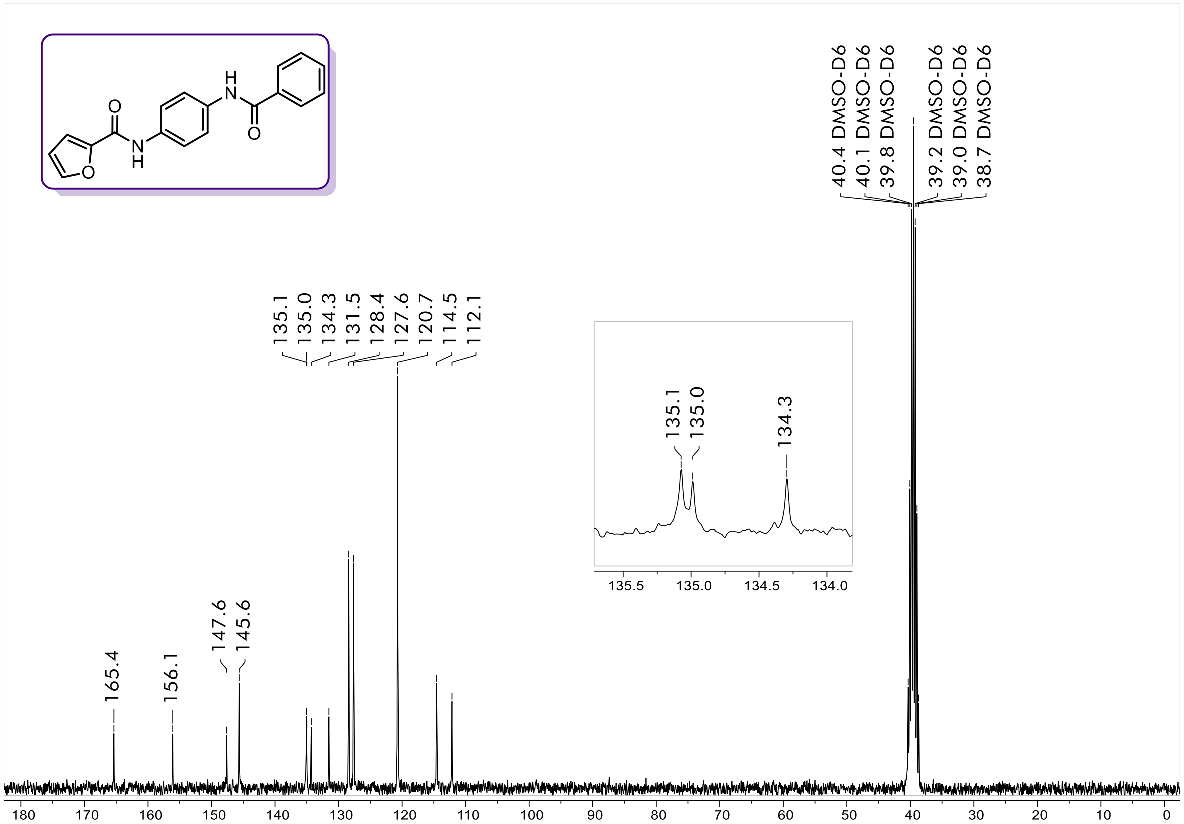

Spectrum 36. ^{13}C-NMR of *N*-(4-benzamidophenyl)furan-2-carboxamide **(5a).**

Description:
Ionization Mode:ESI+
History:Determine m/z[Peak Detect[Centroid,30,Area];Correct Base[];Smooth[5]];Correct Base[5.0%];Average(MS[...

Mass Calibration data:Cal_PEG_600
Created:10/20/2023 12:10:14 PM
Created by:AccuTOF

Charge number:1
Tolerance:4.00(ppm), 5.00 .. 15.00(mmu)
Unsaturation Number:0.0 .. 50.0 (Fraction:Both)
Element:^{12}C:0 .. 18, ^{1}H:0 .. 24, ^{14}N:0 .. 2, ^{16}O:0 .. 3, ^{32}S:0 .. 0, ^{33}S:0 .. 0, ^{34}S:0 .. 0

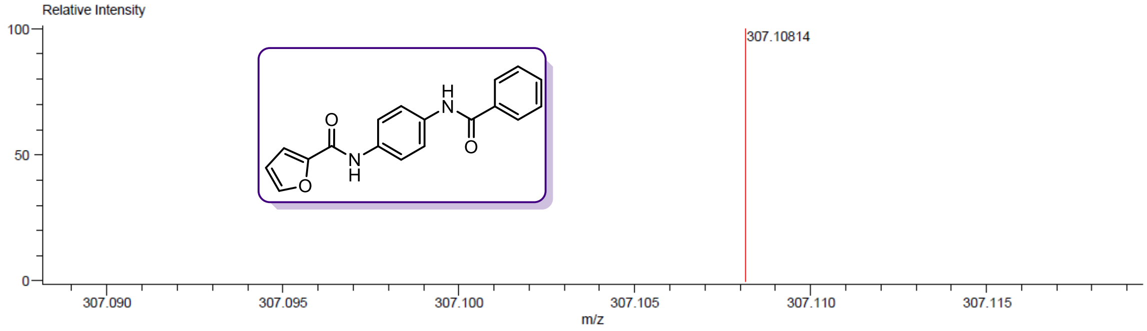

Mass	Intensity	Calc. Mass	Mass Difference (mmu)	Mass Difference (ppm)	Possible Formula	Unsaturation Number
307.10814	15419.69	307.10827	-0.13	-0.43	$^{12}C_{18}{}^{1}H_{15}{}^{14}N_2{}^{16}O_3$	12.5

Spectrum 37. HRMS of *N*-(4-benzamidophenyl)furan-2-carboxamide **(5a).**

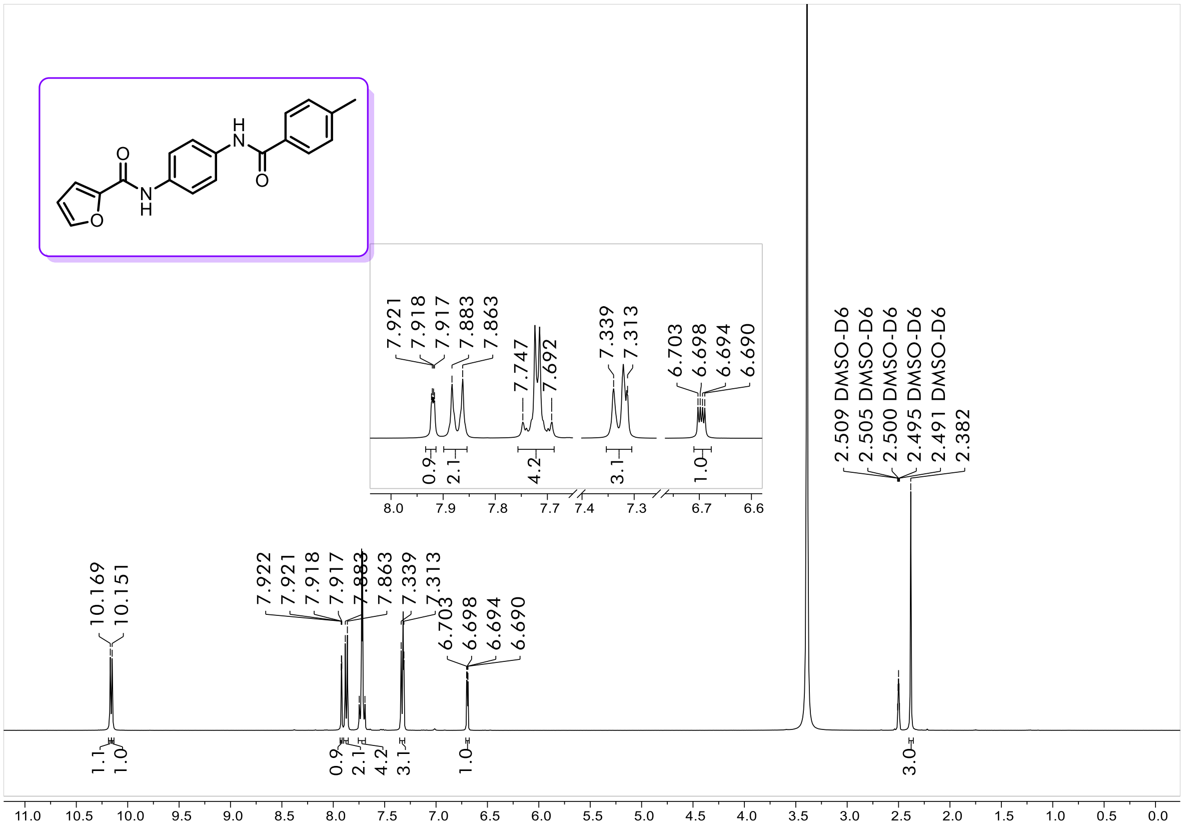

Spectrum 38. ^{1}H-NMR of N-(4-(4-methylbenzamido)phenyl)furan-2-carboxamide (**5b**).

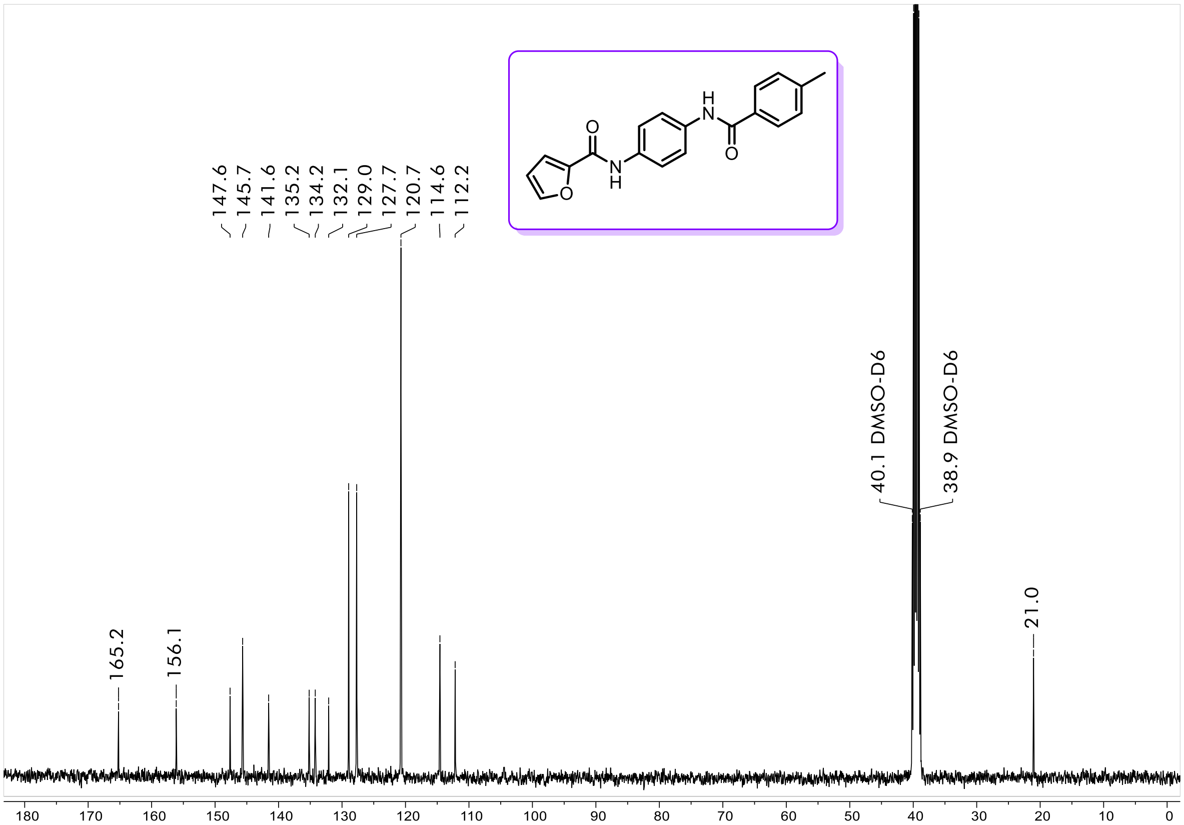

Spectrum 39. ^{13}C-NMR of N-(4-(4-methylbenzamido)phenyl)furan-2-carboxamide (**5b**).

Description:
Ionization Mode:ESI+
History:Determine m/z[Peak Detect[Centroid,30,Area];Correct Base[];Smooth[5]];Correct Base[5.0%];Average(MS[...

Mass Calibration data:Cal_PEG_600
Created:9/19/2023 2:21:19 PM
Created by:AccuTOF

Charge number:1
Tolerance:100.00(ppm), 5.00 .. 15.00(mmu)
Unsaturation Number:-1.0 .. 50.0 (Fraction:Both)
Element:^{12}C:0 .. 19, ^{1}H:0 .. 20, ^{35}Cl:0 .. 0, ^{14}N:0 .. 2, ^{16}O:0 .. 3

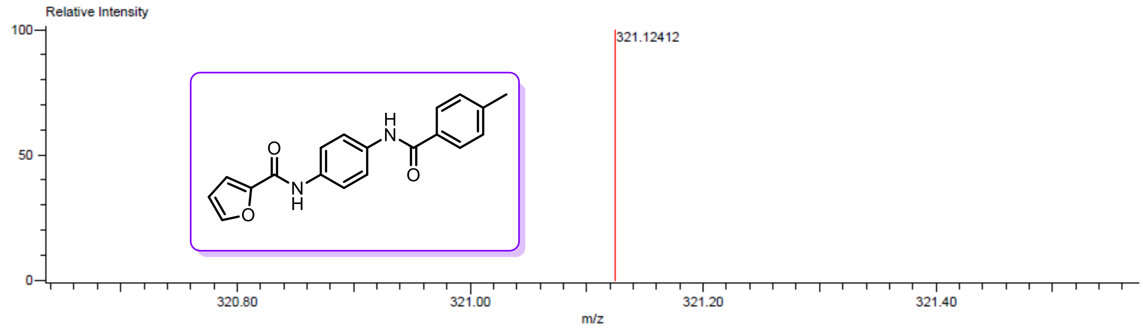

Mass	Intensity	Calc. Mass	Mass Difference (mmu)	Mass Difference (ppm)	Possible Formula	Unsaturation Number
321.12412	121794.26	321.12392	0.20	0.64	$^{12}C_{19}{}^{1}H_{17}{}^{14}N_2{}^{16}O_3$	12.5

Spectrum 40. HRMS of N-(4-(4-methylbenzamido)phenyl)furan-2-carboxamide (**5b**).

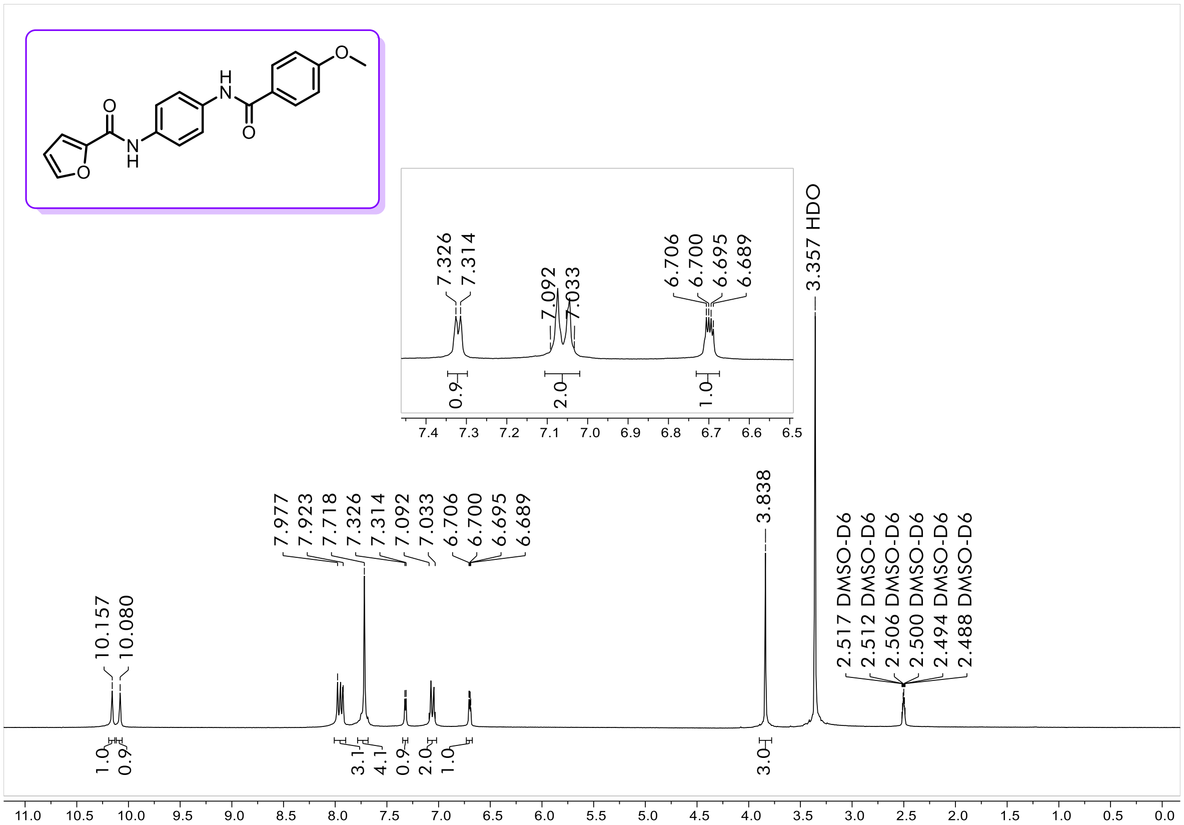

Spectrum 41. ^{1}H-NMR of *N*-(4-(4-methoxybenzamido)phenyl)furan-2-carboxamide (**5c**).

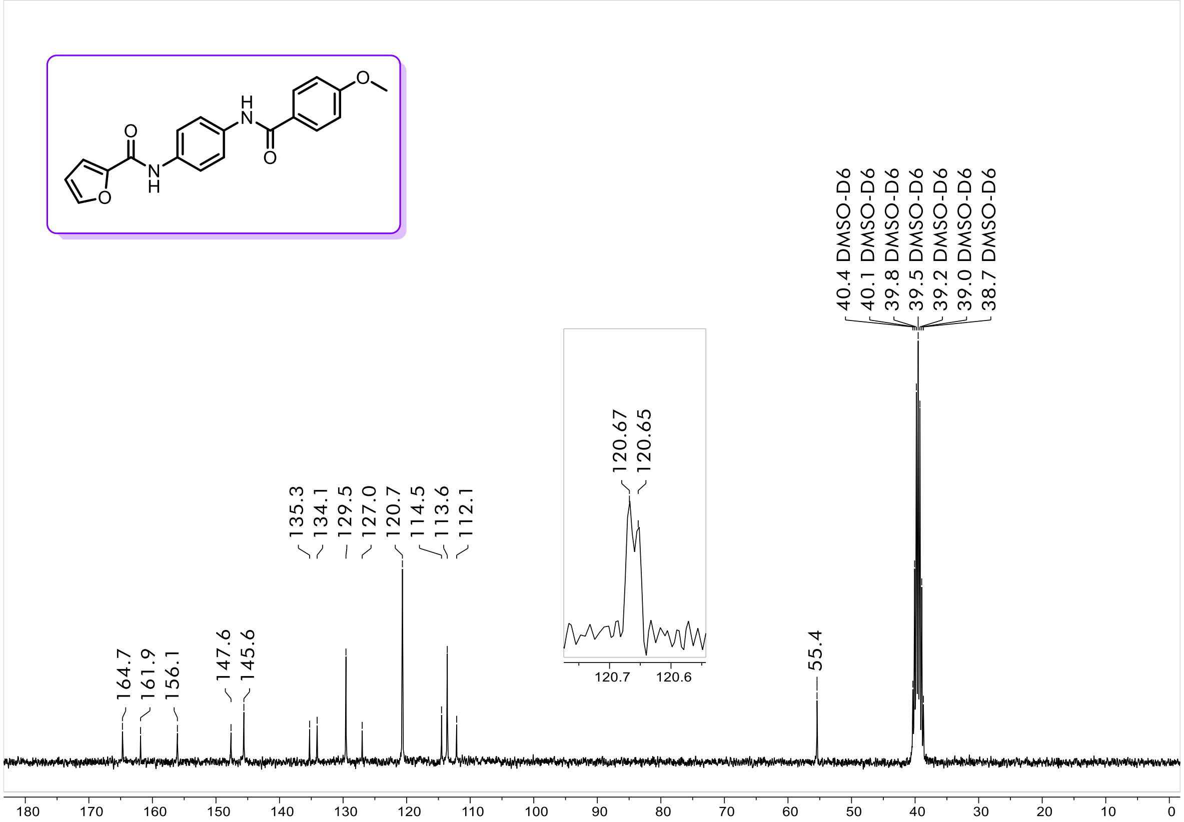

Spectrum 42. ^{13}C-NMR of *N*-(4-(4-methoxybenzamido)phenyl)furan-2-carboxamide (**5c**).

Description:
Ionization Mode:ESI+
History:Determine m/z[Peak Detect[Centroid,30,Area];Correct Base[];Smooth[5]];Correct Base[5.0%];Average(MS[...

Mass Calibration data:cal-PEG-600-nuevo-
Created:9/14/2022 4:03:34 PM
Created by:AccuTOF

Charge number:1
Tolerance:100.00(ppm), 5.00 .. 15.00(mmu)
Unsaturation Number:0.0 .. 100.0 (Fraction:Both)
Element:^{12}C:10 .. 19, ^{1}H:0 .. 30, ^{19}F:0 .. 0, ^{14}N:1 .. 2, ^{16}O:0 .. 4

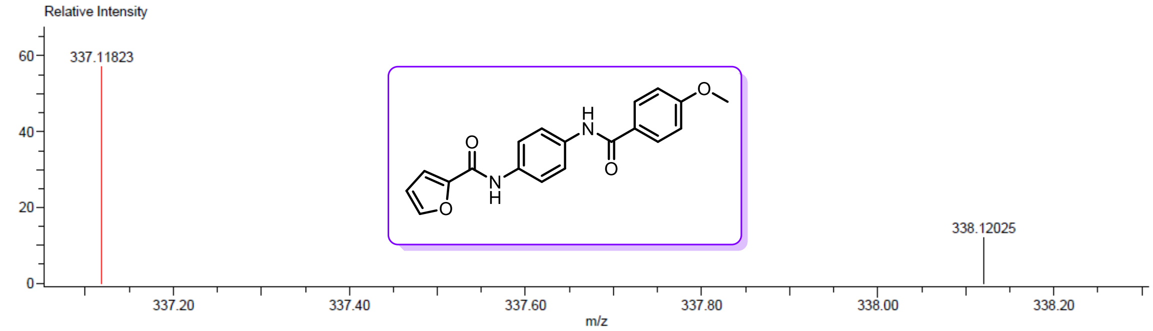

Mass	Intensity	Calc. Mass	Mass Difference (mmu)	Mass Difference (ppm)	Possible Formula	Unsaturation Number
337.11823	836337.24	337.11883	-0.60	-1.79	$^{12}C_{19}{}^{1}H_{17}{}^{14}N_2{}^{16}O_4$	12.5

Spectrum 43. HRMS of *N*-(4-(4-methoxybenzamido)phenyl)furan-2-carboxamide (**5c**).

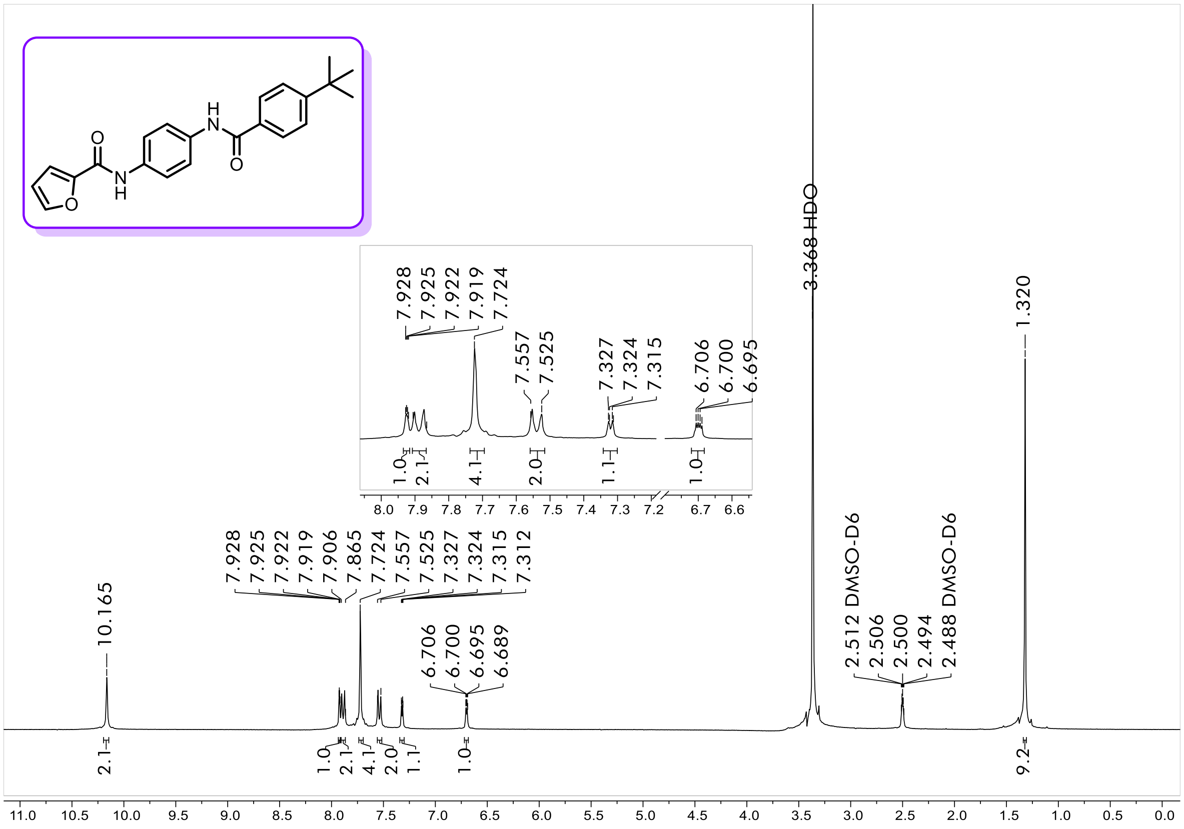

Spectrum 44. ^{1}H-NMR of *N*-(4-(4-(*tert*-butyl)benzamido)phenyl)furan-2-carboxamide (**5d**).

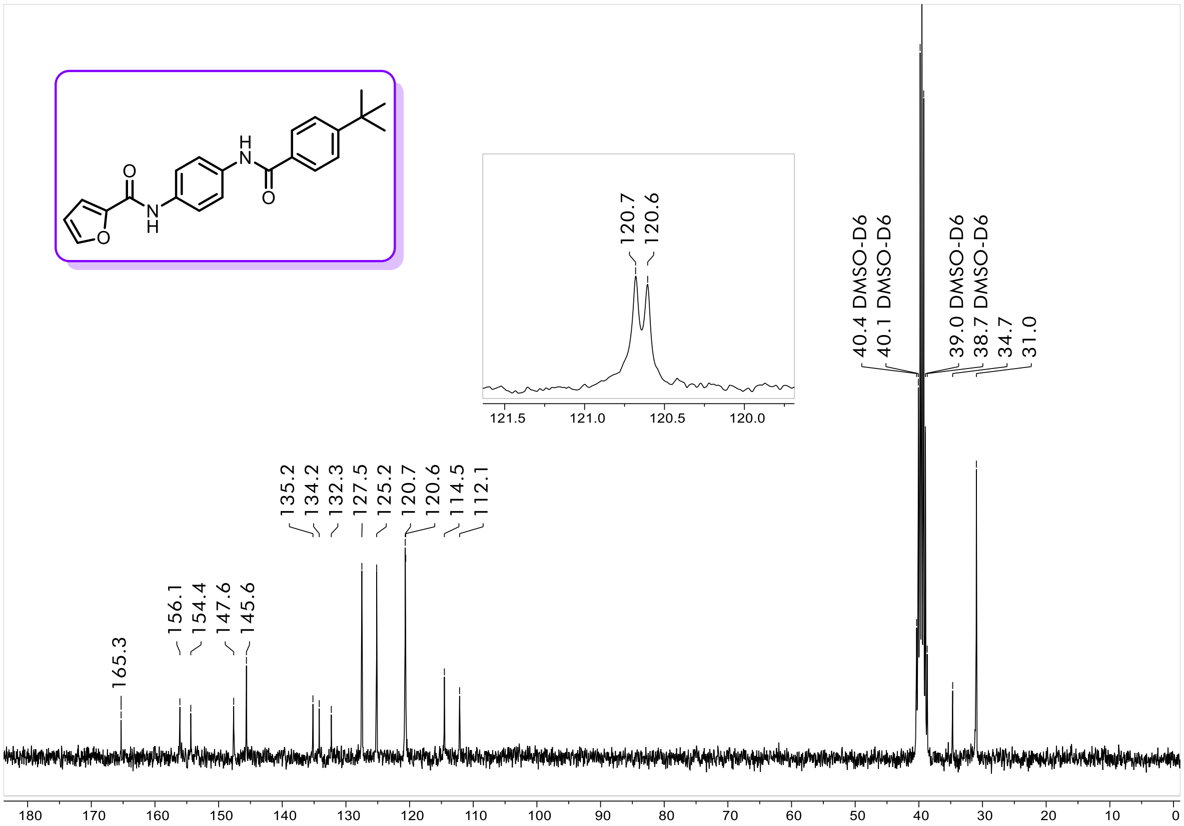

Spectrum 45. ^{13}C-NMR of *N*-(4-(4-(*tert*-butyl)benzamido)phenyl)furan-2-carboxamide (**5d**).

Description:
Ionization Mode:ESI+
History:Determine m/z[Peak Detect[Centroid,30,Area];Correct Base[];Smooth[5]];Correct Base[5.0%];Average(MS[...

Mass Calibration data:Cal_PEG_600
Created:10/26/2023 11:10:15 AM
Created by:AccuTOF

Charge number:1
Element:^{12}C:0 .. 22, ^{1}H:0 .. 23, ^{14}N:0 .. 2, ^{16}O:0 .. 3

Tolerance:100.00(ppm), 5.00 .. 15.00(mmu)

Unsaturation Number:0.0 .. 50.0 (Fraction:.5)

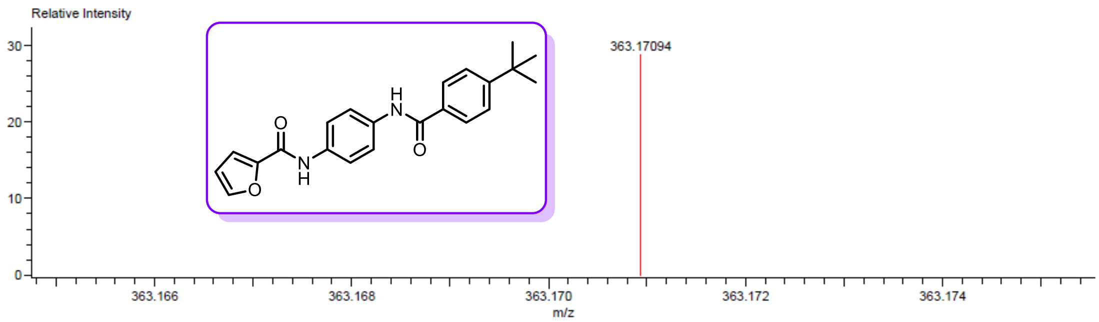

Mass	Intensity	Calc. Mass	Mass Difference (mmu)	Mass Difference (ppm)	Possible Formula	Unsaturation Number
363.17094	86412.72	363.17087	0.07	0.19	$^{12}C_{22}{}^{1}H_{23}{}^{14}N_{2}{}^{16}O_{3}$	12.5

Spectrum 45. HRMS of *N*-(4-(4-(*tert*-butyl)benzamido)phenyl)furan-2-carboxamide (**5d**).

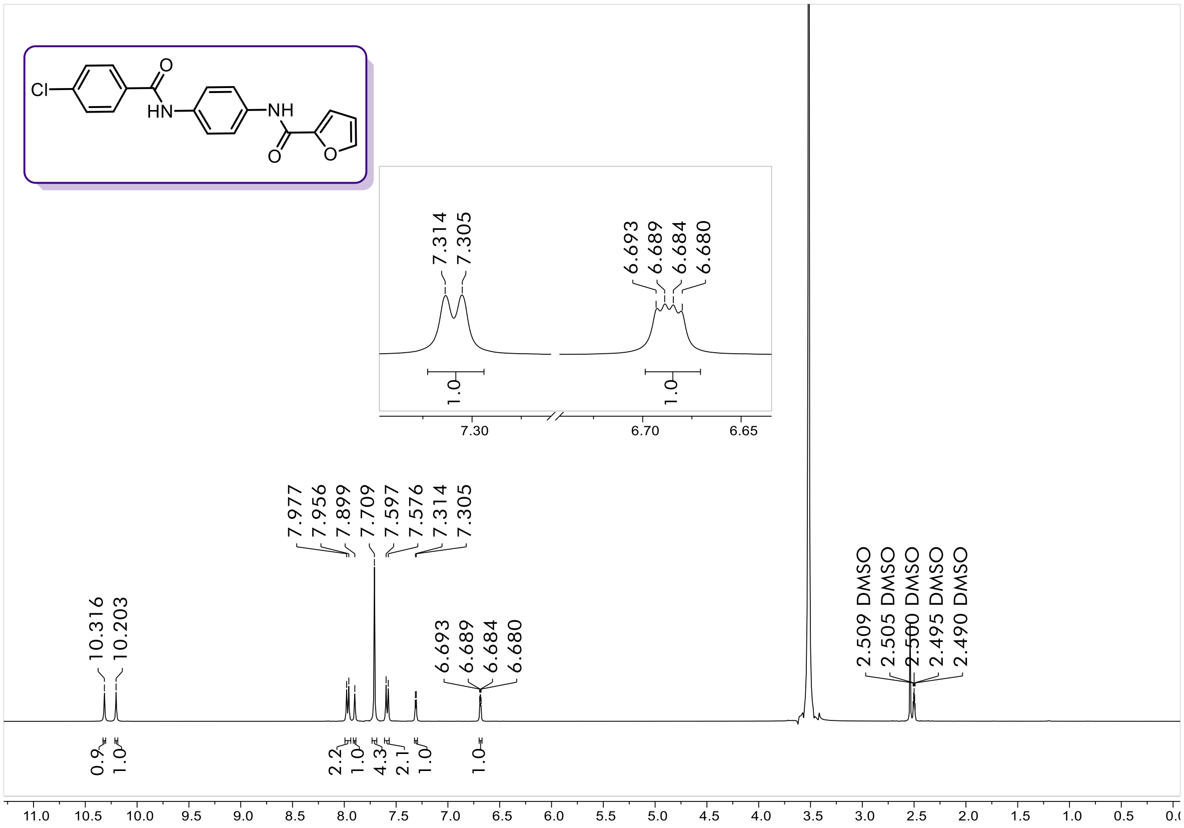

Spectrum 46. ^{1}H-NMR of *N*-(4-(4-chlorobenzamido)phenyl)furan-2-carboxamide (**5e**).

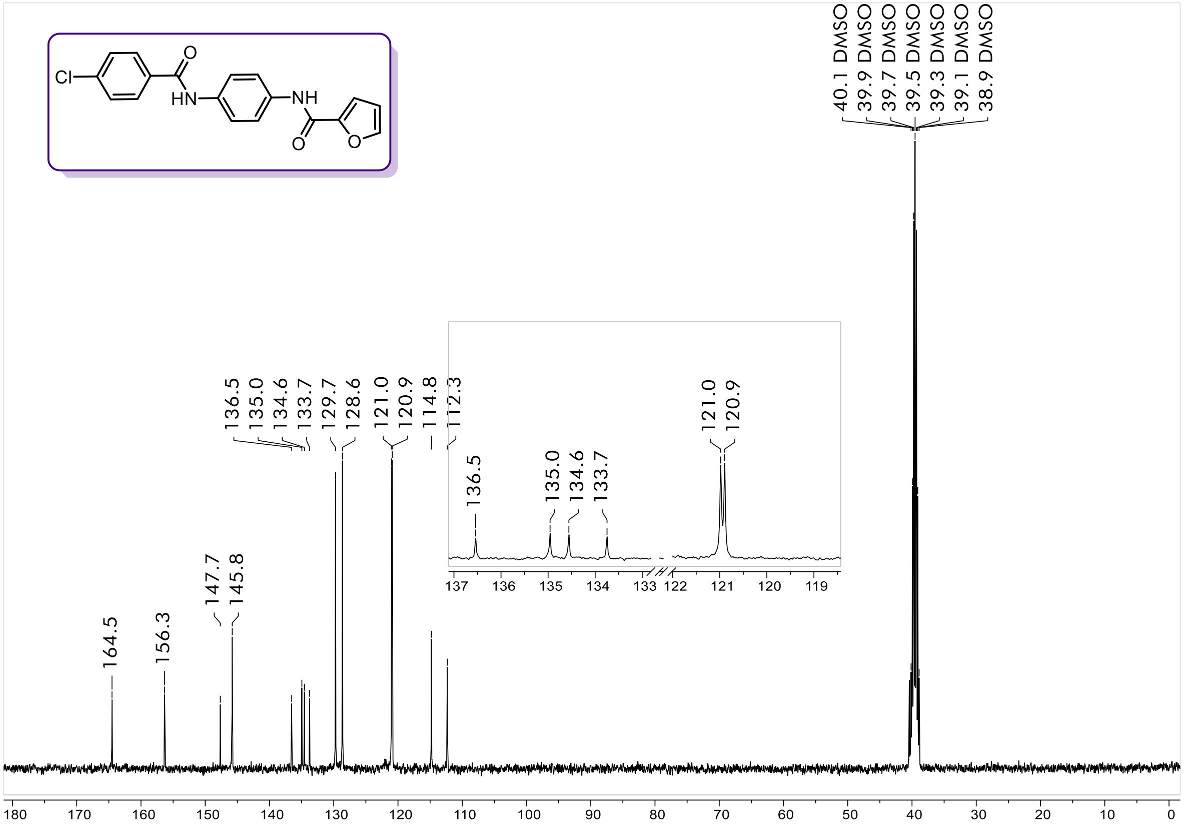

Spectrum 47. ^{13}C-NMR of *N*-(4-(4-chlorobenzamido)phenyl)furan-2-carboxamide (**5e**).

Description:
Ionization Mode:ESI+
History:Determine m/z[Peak Detect[Centroid,30,Area];Correct Base[];Smooth[5]];Correct Base[5.0%];Average(MS[...

Mass Calibration data:cal-PEG-600-nuevo
Created:9/14/2022 4:18:04 PM
Created by:AccuTOF

Charge number:1
Tolerance:100.00(ppm), 5.00 .. 15.00(mmu)
Unsaturation Number:0.0 .. 100.0 (Fraction:Both)
Element:^{12}C:10 .. 18, ^{1}H:0 .. 30, ^{35}Cl:0 .. 1, ^{14}N:1 .. 2, ^{16}O:0 .. 3

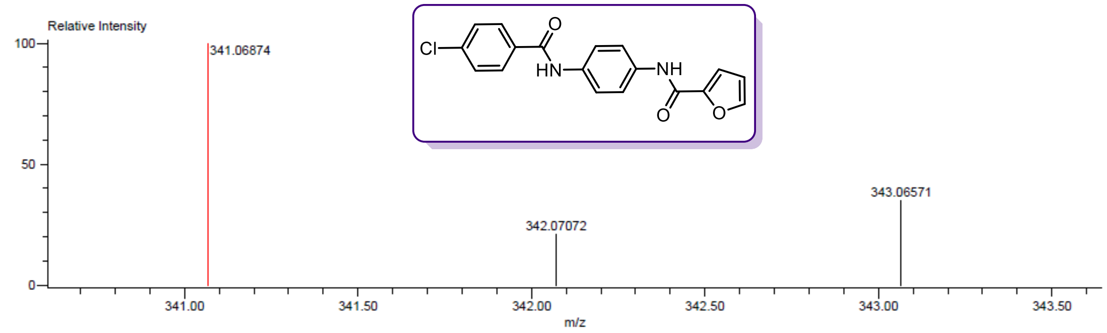

Mass	Intensity	Calc. Mass	Mass Difference (mmu)	Mass Difference (ppm)	Possible Formula	Unsaturation Number
341.06874	2418136.80	341.06929	-0.55	-1.61	$^{12}C_{18}{}^{1}H_{14}{}^{35}Cl_{1}{}^{14}N_{2}{}^{16}O_{3}$	12.5

Spectrum 48. HRMS of *N*-(4-(4-chlorobenzamido)phenyl)furan-2-carboxamide (**5e**).

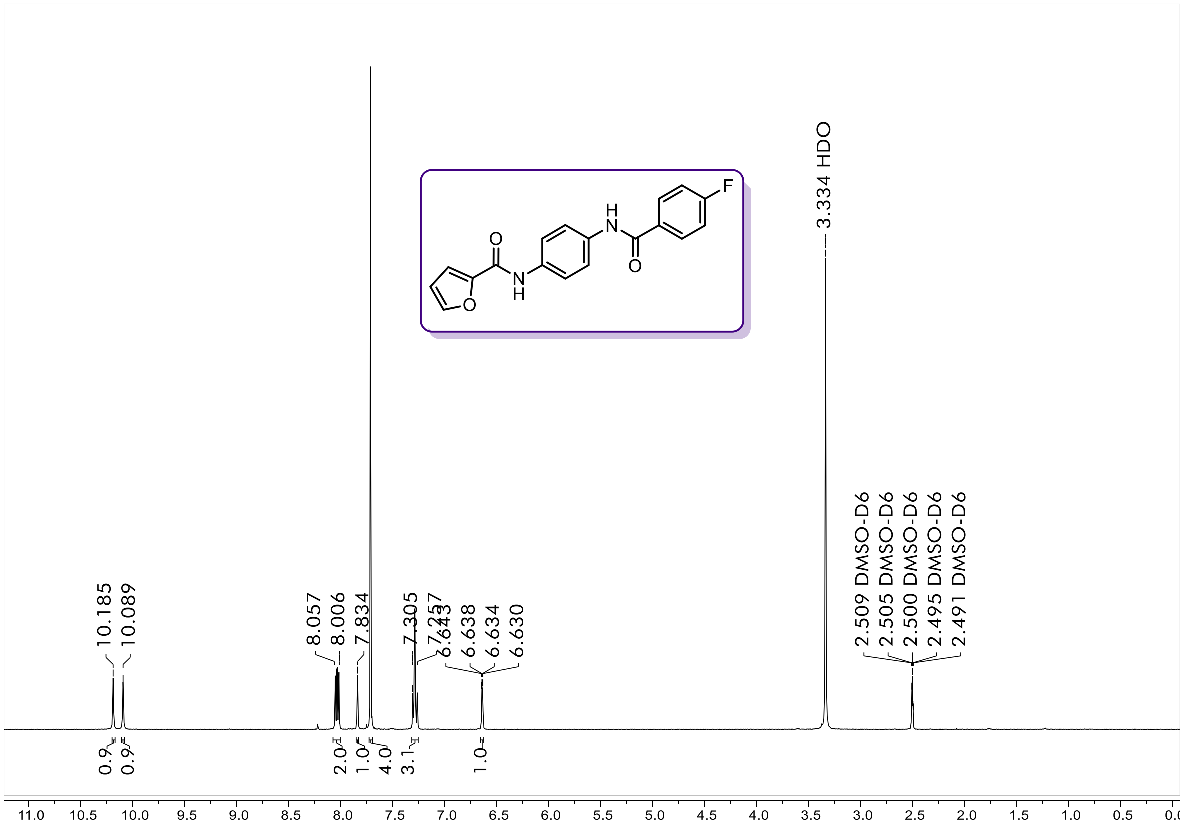

Spectrum 49. ^{1}H-NMR of *N*-(4-(4-fluorobenzamido)phenyl)furan-2-carboxamide (**5f**).

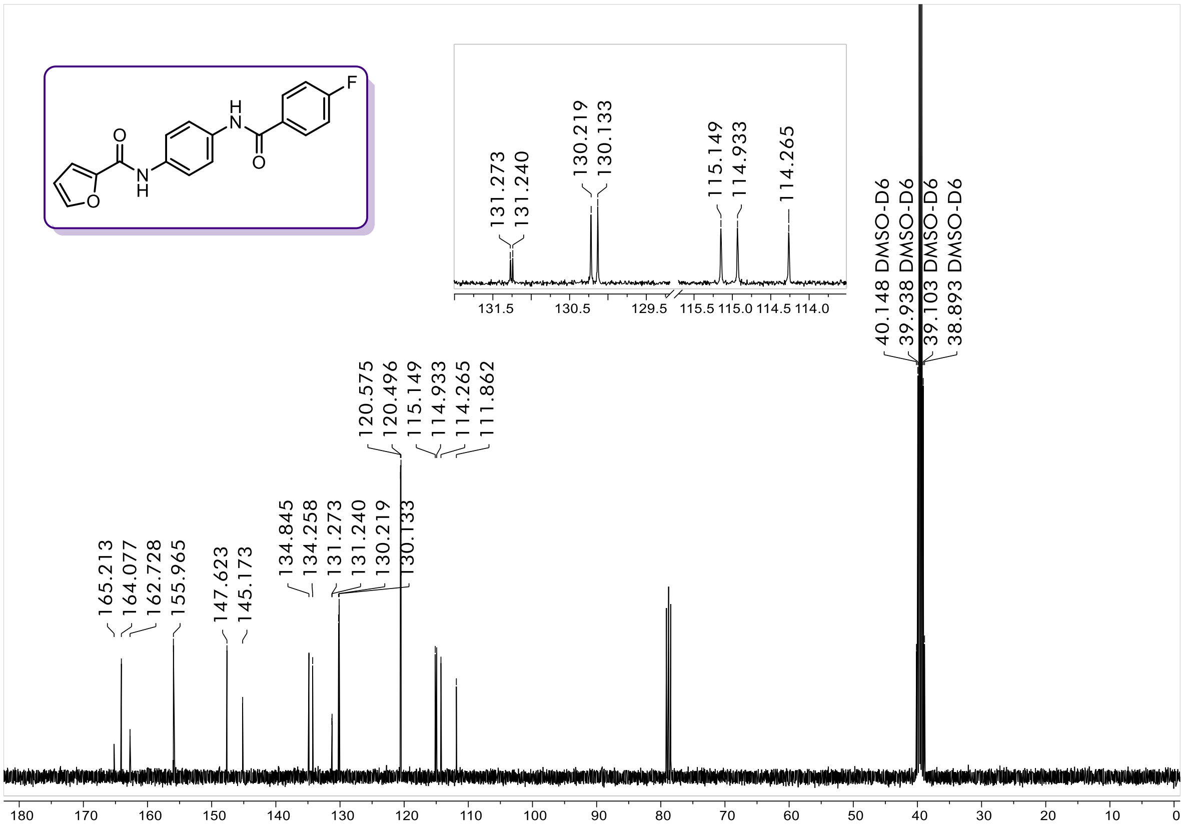

Spectrum 50. ^{13}C-NMR of *N*-(4-(4-fluorobenzamido)phenyl)furan-2-carboxamide (**5f**).

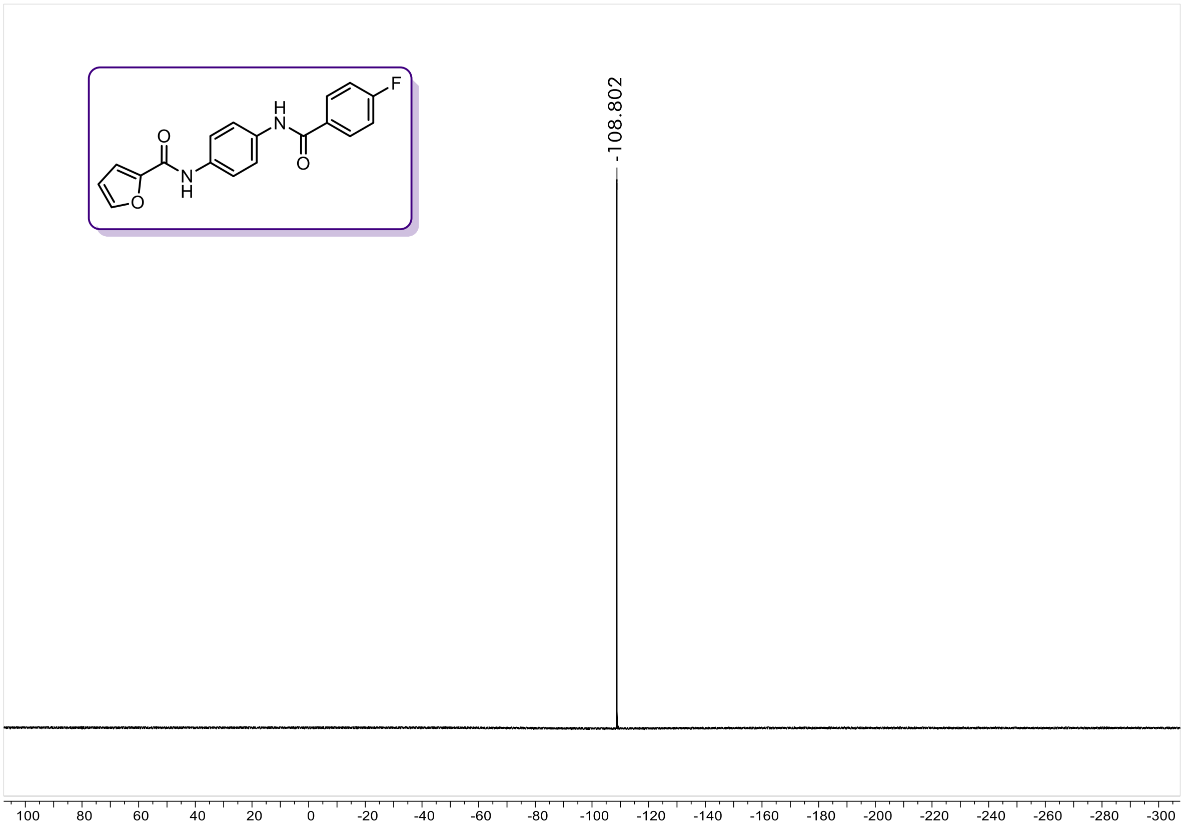

Spectrum 51. ^{19}F-NMR of *N*-(4-(4-fluorobenzamido)phenyl)furan-2-carboxamide (**5f**).

Description:
Ionization Mode:ESI+
History:Determine m/z[Peak Detect[Centroid,30,Area];Correct Base[];Smooth[5]];Correct Base[5.0%];Average(MS[...

Mass Calibration data:cal-PEG-600-nuevo
Created:9/7/2022 3:28:09 PM
Created by:AccuTOF

Charge number:1
Tolerance:4.00(mmu)
Unsaturation Number:0.0 .. 50.0 (Fraction:.5)
Element:^{12}C:10 .. 18, ^{1}H:10 .. 26, ^{35}Cl:0 .. 0, ^{19}F:0 .. 1, ^{14}N:1 .. 2, ^{16}O:1 .. 3

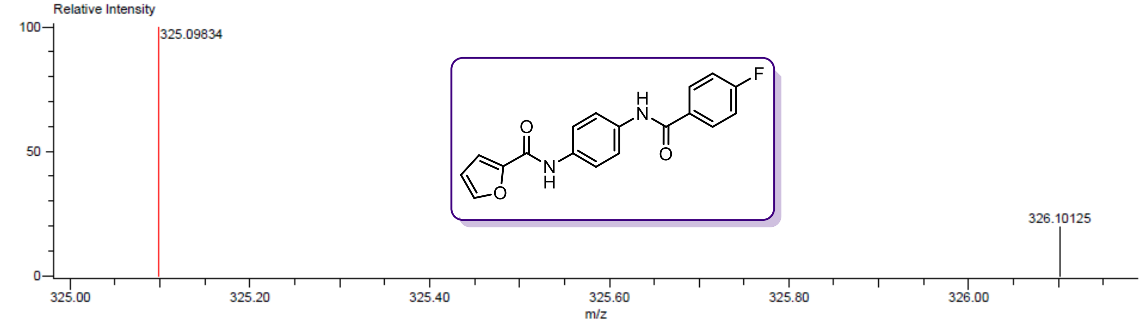

Mass	Intensity	Calc. Mass	Mass Difference (mmu)	Mass Difference (ppm)	Possible Formula	Unsaturation Number
325.09834	423060.94	325.09885	-0.50	-1.54	$^{12}C_{18}{}^{1}H_{14}{}^{19}F_{1}{}^{14}N_{2}{}^{16}O_{3}$	12.5

Spectrum 52. ^{19}F-NMR of *N*-(4-(4-fluorobenzamido)phenyl)furan-2-carboxamide (**5f**).

NMR SPECTRA OF ACYLAMINOBENZAMIDES 6a-e

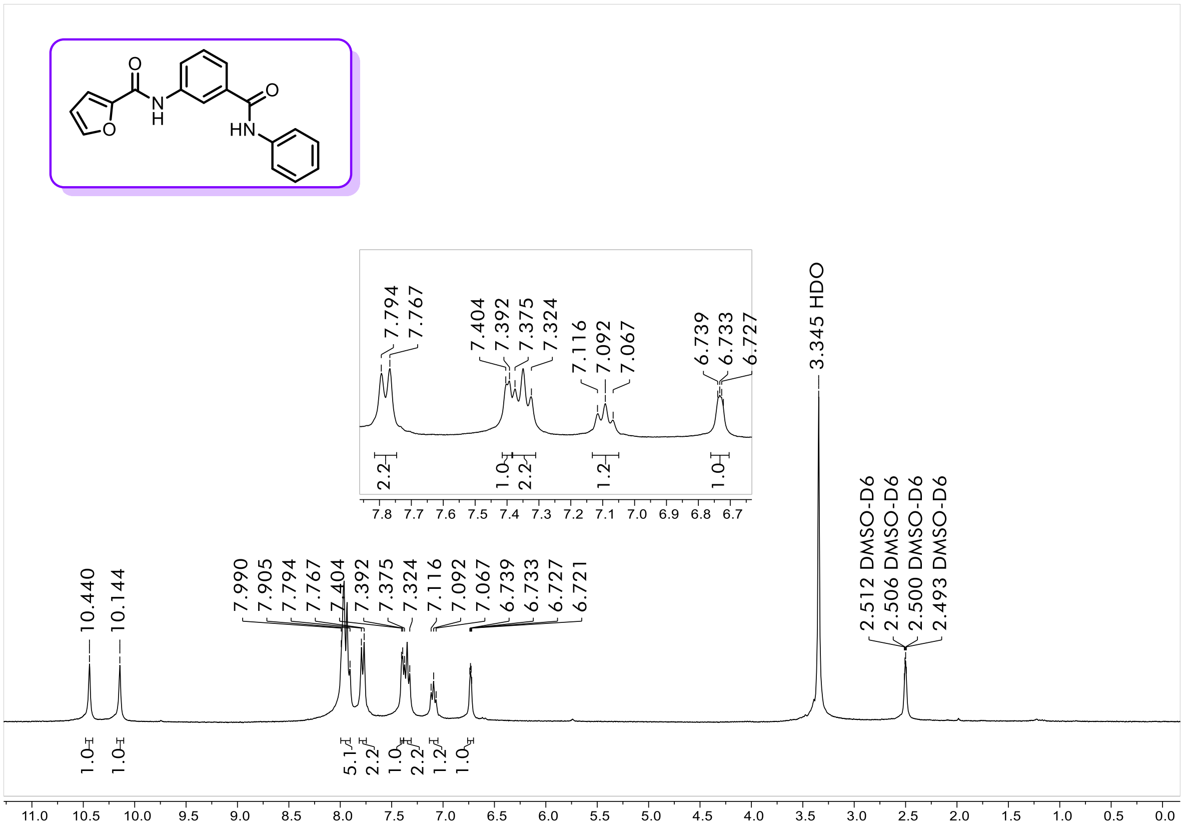

Spectrum 53. ^{1}H-NMR of *N*-(3-(phenylcarbamoyl)phenyl)furan-2-carboxamide (**6a**).

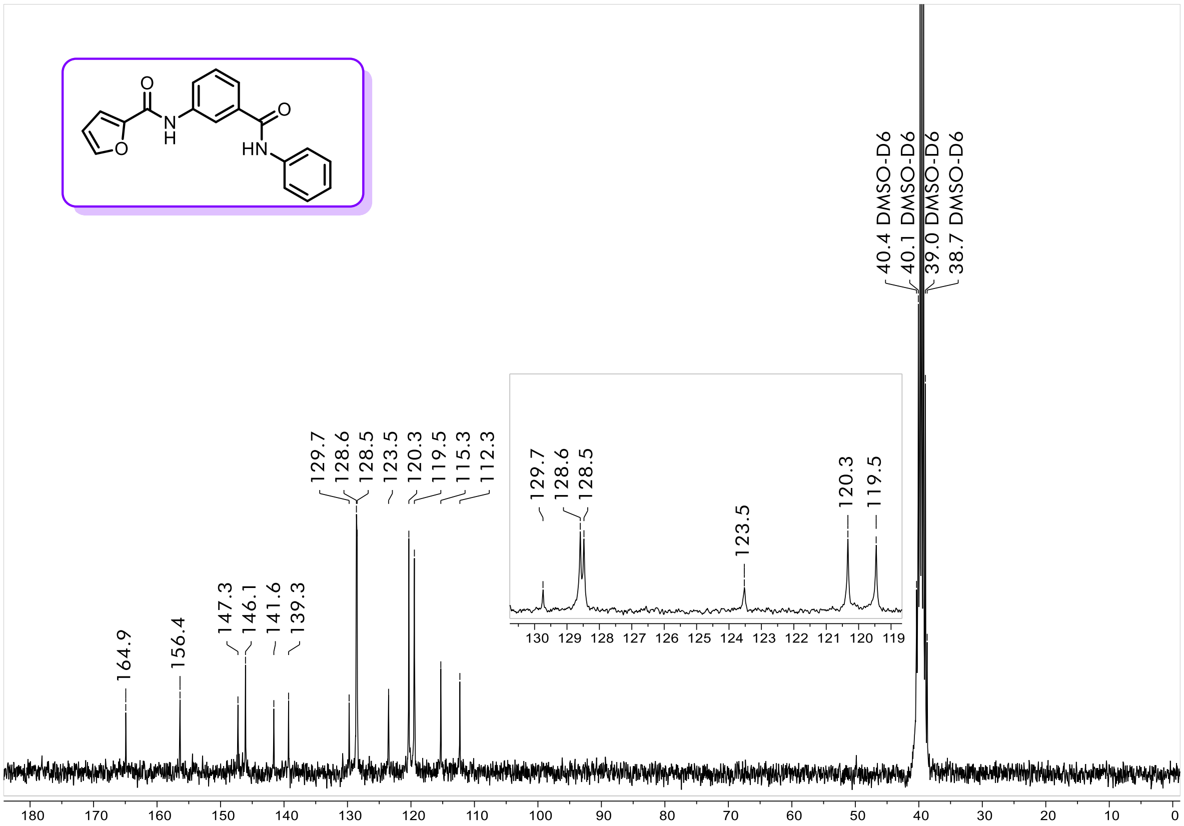

Spectrum 54. ^{13}C-NMR of *N*-(3-(phenylcarbamoyl)phenyl)furan-2-carboxamide (**6a**).

Description:
Ionization Mode:ESI+
History:Determine m/z[Peak Detect[Centroid,30,Area];Correct Base[];Smooth[5]];Correct Base[5.0%];Average(MS[...

Mass Calibration data:Cal_PEG_600
Created:8/18/2023 11:05:53 AM
Created by:AccuTOF

Charge number:1
Element:^{12}C:0 .. 18, ^{1}H:0 .. 60, ^{14}N:0 .. 2, ^{16}O:3 .. 3

Tolerance:30.00(ppm), 5.00 .. 15.00(mmu)

Unsaturation Number:-1.0 .. 50.0 (Fraction:Both)

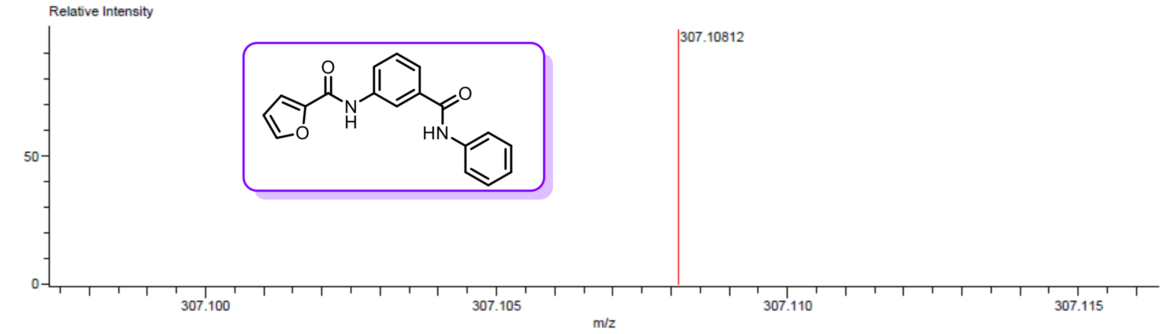

Mass	Intensity	Calc. Mass	Mass Difference (mmu)	Mass Difference (ppm)	Possible Formula	Unsaturation Number
307.10812	1064902.55	307.10827	-0.14	-0.46	$^{12}C_{18}{}^{1}H_{15}{}^{14}N_{2}{}^{16}O_{3}$	12.5

Spectrum 55. HRMS of *N*-(3-(phenylcarbamoyl)phenyl)furan-2-carboxamide (**6a**).

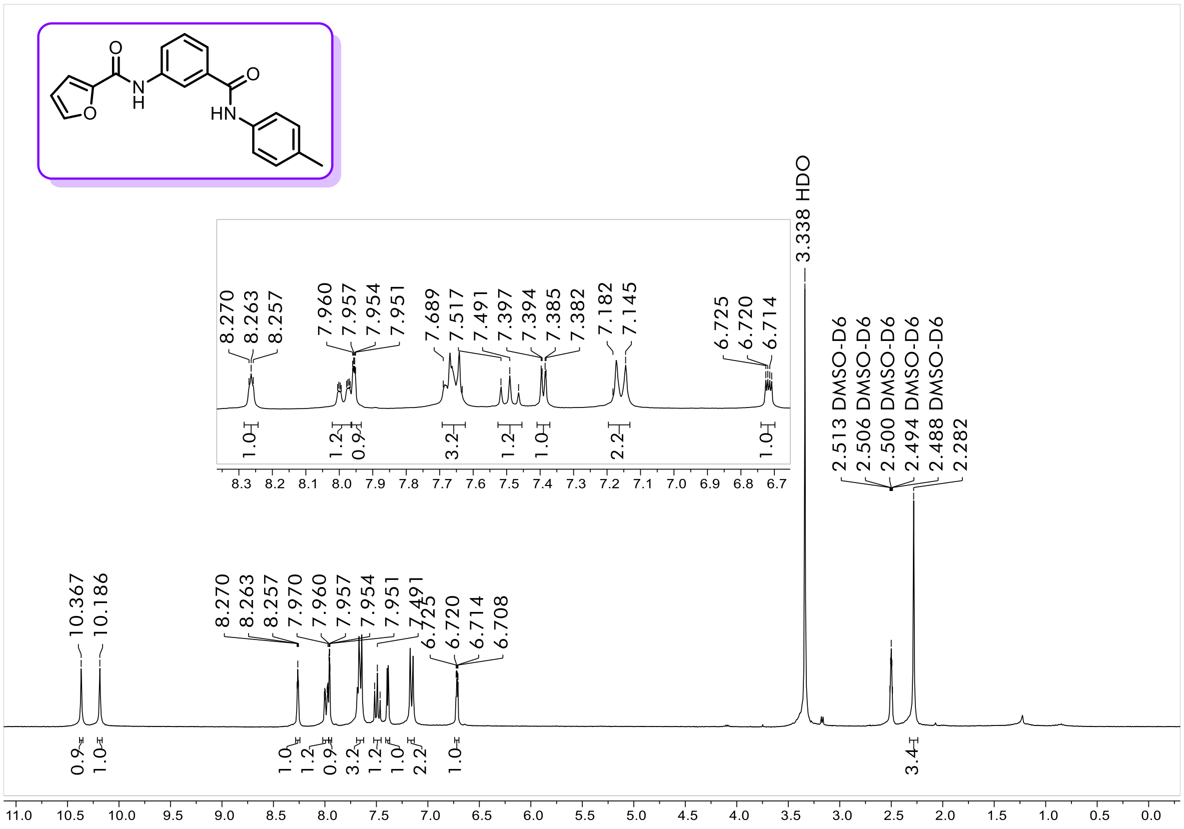

Spectrum 56. ¹H-NMR of *N*-(3-((4-methylphenyl)carbamoyl)phenyl)furan-2-carboxamide (**6b**).

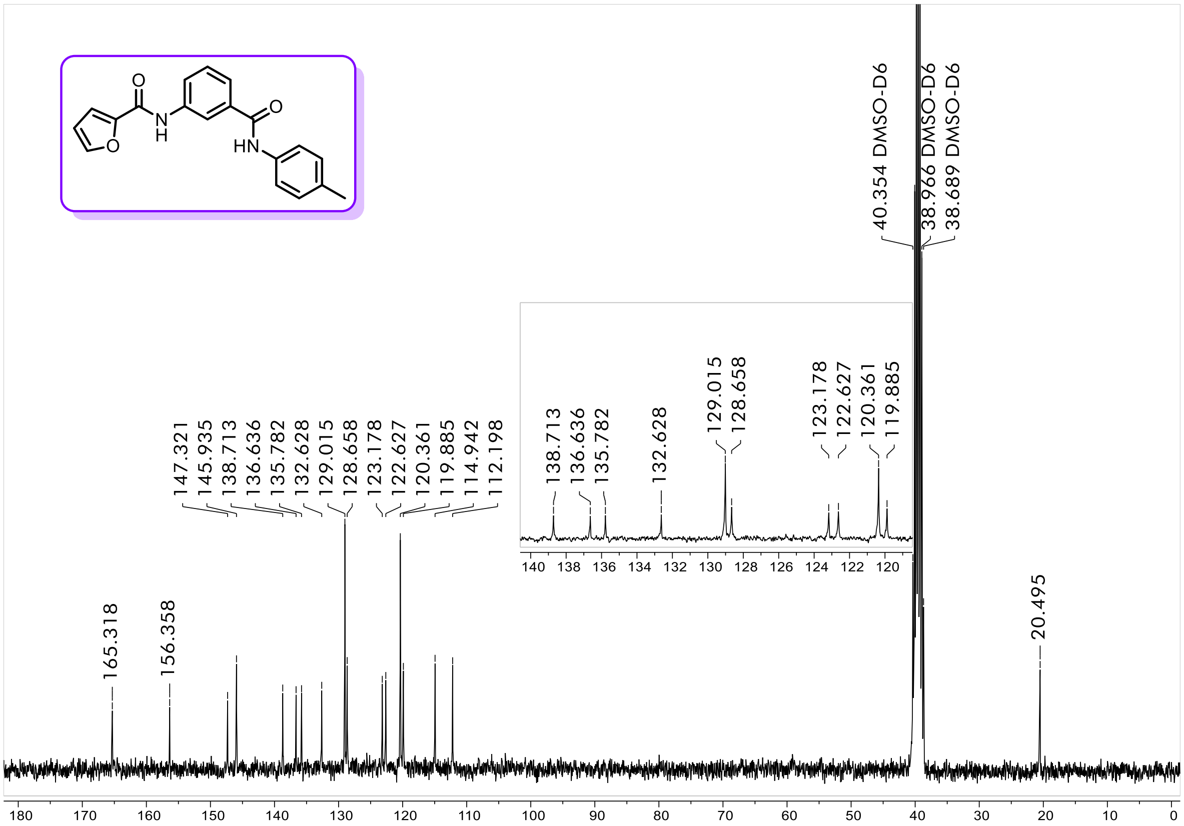

Spectrum 57. ¹³C-NMR of *N*-(3-((4-methylphenyl)carbamoyl)phenyl)furan-2-carboxamide (**6b**).

Description:
Ionization Mode:ESI+
History:Determine m/z[Peak Detect[Centroid,30,Area];Correct Base[];Smooth[5]];Correct Base[5.0%];Average(MS[...

Mass Calibration data:Cal_PEG_600
Created:8/18/2023 11:20:17 AM
Created by:AccuTOF

Charge number:1
Element:^{12}C:0 .. 19, ^{1}H:0 .. 60, ^{14}N:0 .. 2, ^{16}O:3 .. 3

Tolerance:30.00(ppm), 5.00 .. 15.00(mmu)

Unsaturation Number:-1.0 .. 50.0 (Fraction:Both)

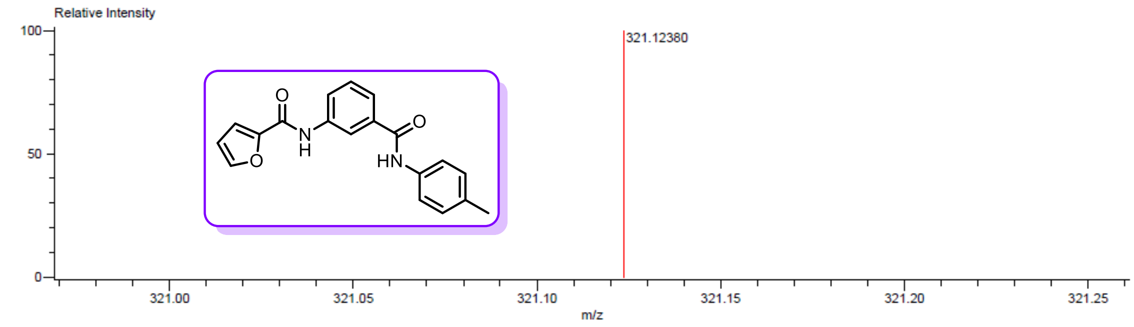

Mass	Intensity	Calc. Mass	Mass Difference (mmu)	Mass Difference (ppm)	Possible Formula	Unsaturation Number
321.12380	3938207.04	321.12392	-0.12	-0.36	$^{12}C_{19}{}^{1}H_{17}{}^{14}N_2{}^{16}O_3$	12.5

Spectrum 58. HRMS of *N*-(3-((4-methylphenyl)carbamoyl)phenyl)furan-2-carboxamide (**6b**).

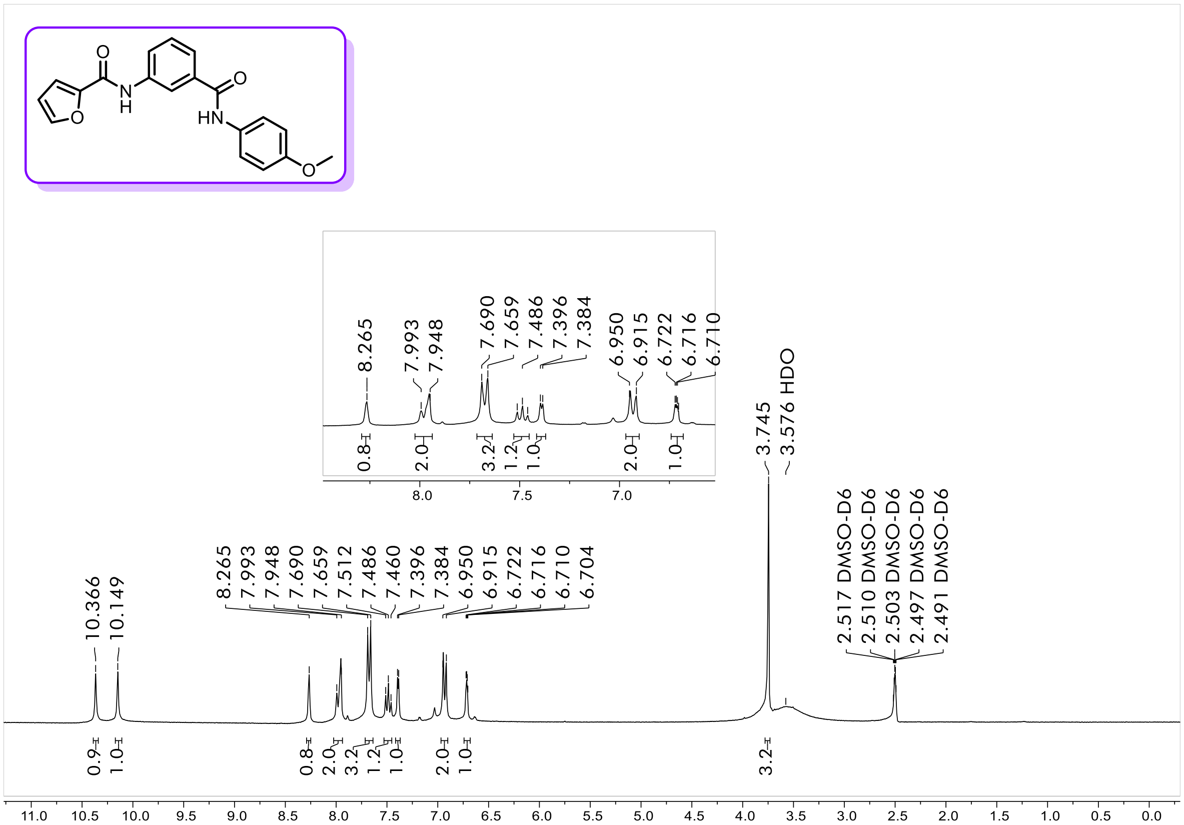

Spectrum 59. ^{1}H-NMR of *N*-(3-((4-methoxyphenyl)carbamoyl)phenyl)furan-2-carboxamide (**6c**).

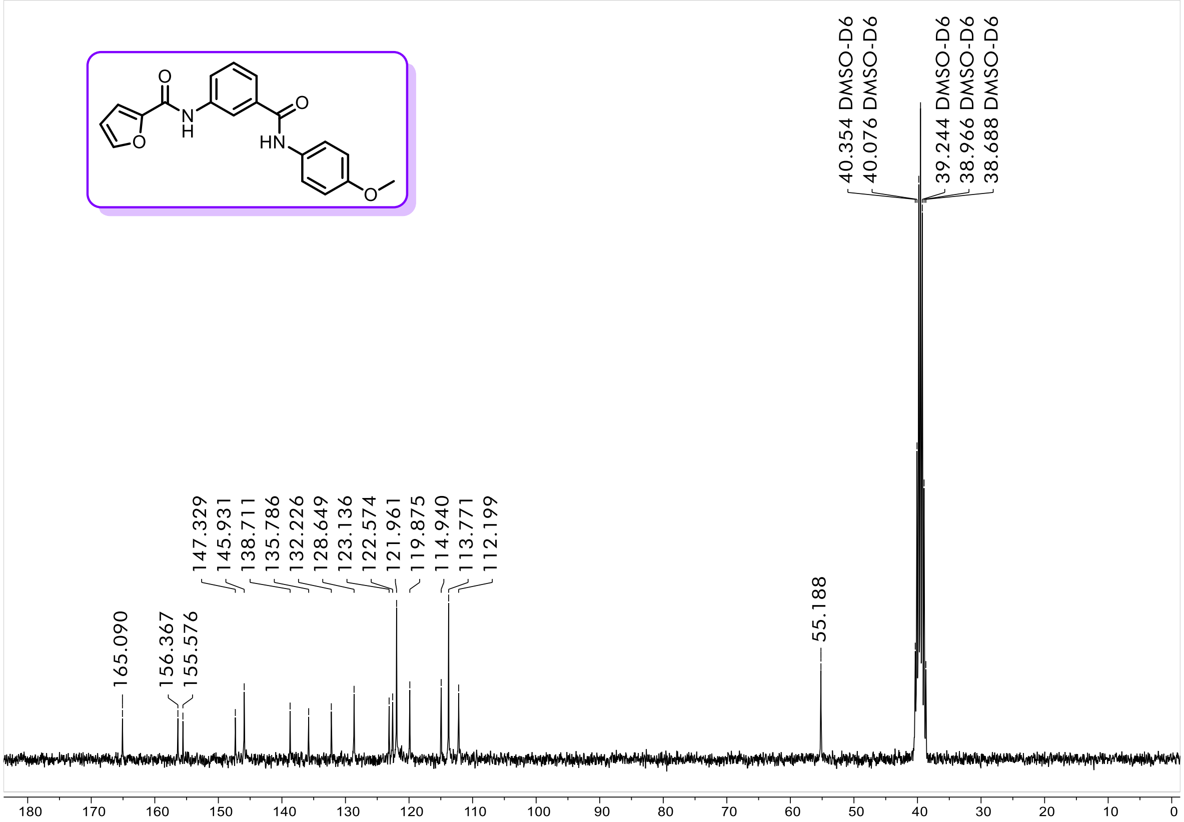

Spectrum 60. ^{13}C-NMR of *N*-(3-((4-methoxyphenyl)carbamoyl)phenyl)furan-2-carboxamide (**6c**).

Description:
Ionization Mode:ESI+
History:Determine m/z[Peak Detect[Centroid,30,Area];Correct Base[];Smooth[5]];Correct Base[5.0%];Average(MS[...

Mass Calibration data:Cal_PEG_600
Created:6/6/2023 10:48:23 AM
Created by:AccuTOF

Charge number:1
Element:^{12}C:12 .. 19, ^{1}H:13 .. 30, ^{14}N:0 .. 2, ^{16}O:2 .. 4

Tolerance:100.00(ppm), 5.00 .. 15.00(mmu)

Unsaturation Number:-1.0 .. 60.0 (Fraction:Both)

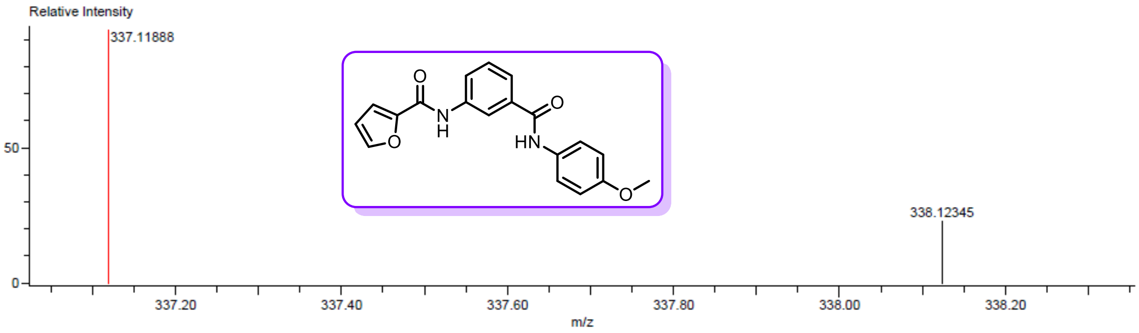

Mass	Intensity	Calc. Mass	Mass Difference (mmu)	Mass Difference (ppm)	Possible Formula	Unsaturation Number
337.11888	89341.57	337.11883	0.05	0.15	$^{12}C_{19}{}^{1}H_{17}{}^{14}N_2{}^{16}O_4$	12.5

Spectrum 61. HRMS of *N*-(3-((4-methoxyphenyl)carbamoyl)phenyl)furan-2-carboxamide (**6c**).

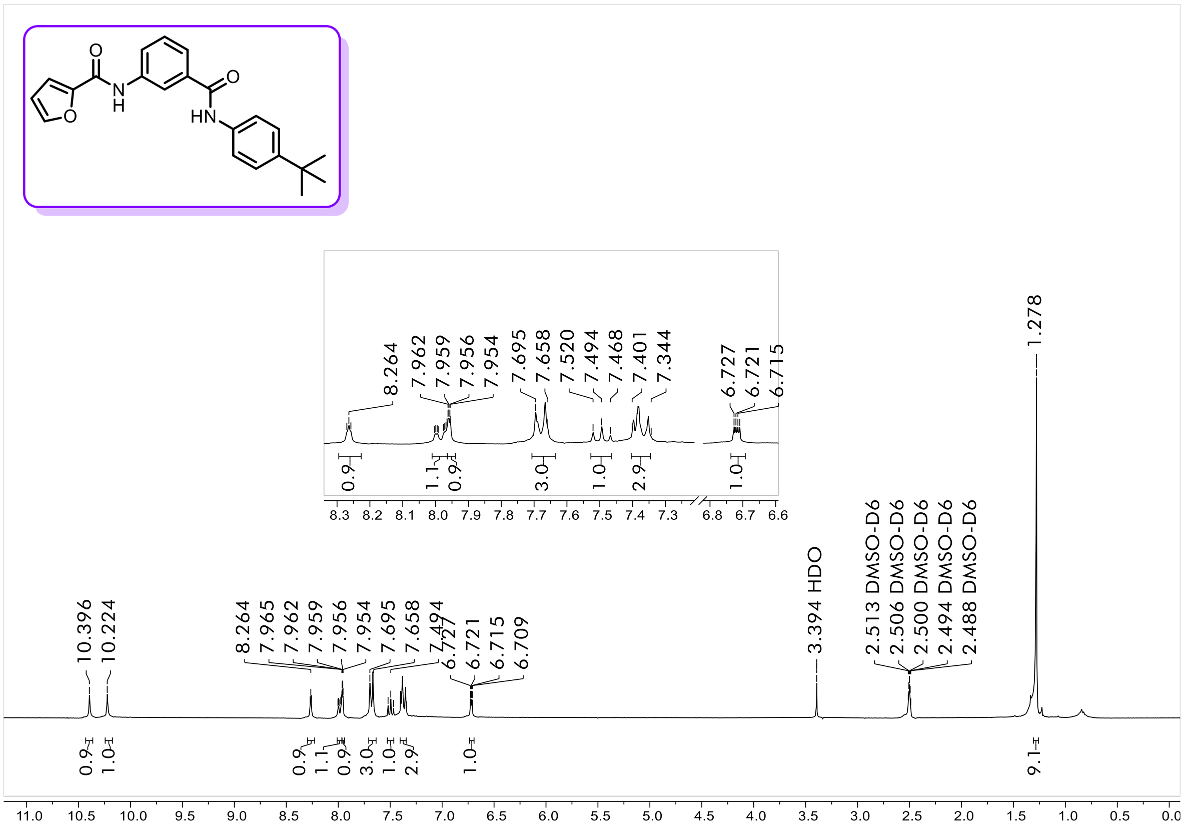

Spectrum 62. ^{1}H-NMR of *N*-(3-((4-(*tert*-butyl)phenyl)carbamoyl)phenyl)furan-2-carboxamide (**6d**).

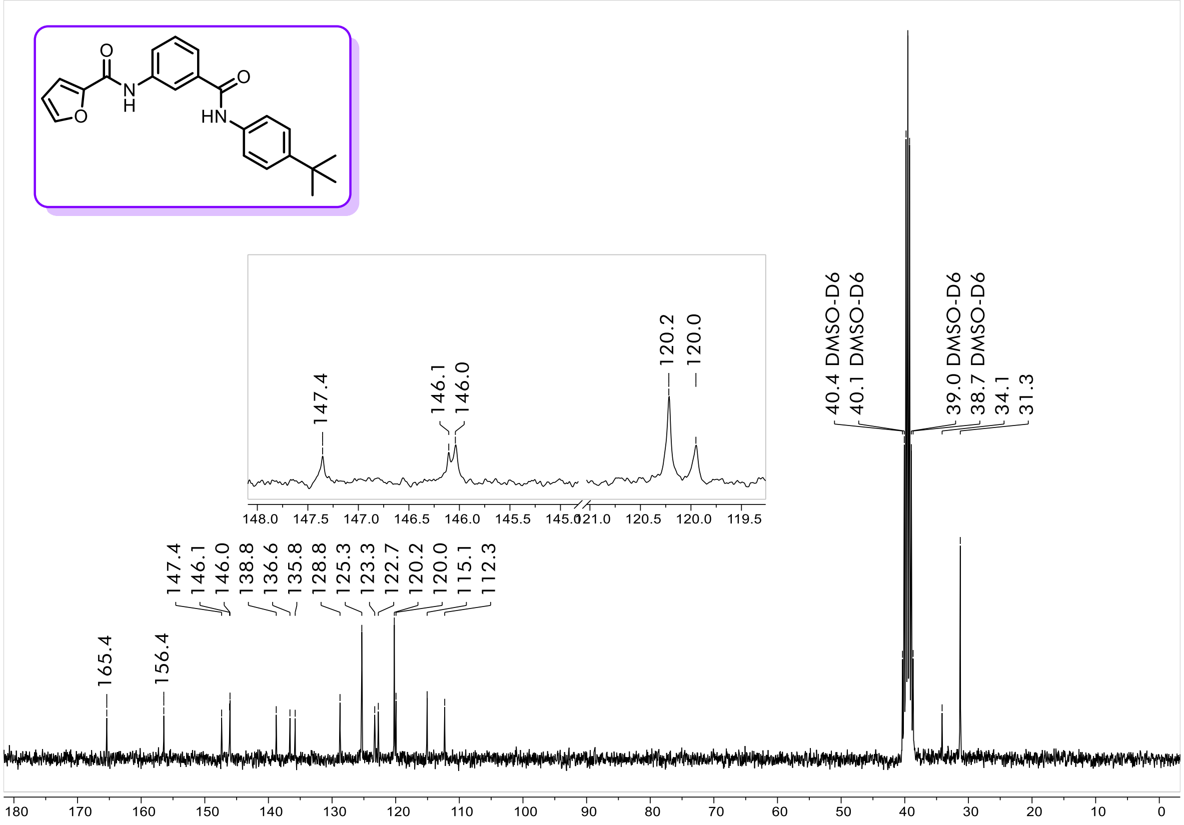

Spectrum 63. ^{13}C-NMR of *N*-(3-((4-(*tert*-butyl)phenyl)carbamoyl)phenyl)furan-2-carboxamide (**6d**).

Description:
Ionization Mode:ESI+
History:Determine m/z[Peak Detect[Centroid,30,Area];Correct Base[];Smooth[5]];Correct Base[5.0%];Average(MS[...

Mass Calibration data:Cal_PEG_600
Created:8/23/2023 1:33:53 PM
Created by:AccuTOF

Charge number:1
Element:^{12}C:0 .. 22, ^{1}H:0 .. 23, ^{14}N:0 .. 2, ^{16}O:0 .. 3

Tolerance:5.00(ppm), 5.00 .. 15.00(mmu)

Unsaturation Number:-1.0 .. 13.0 (Fraction:Both)

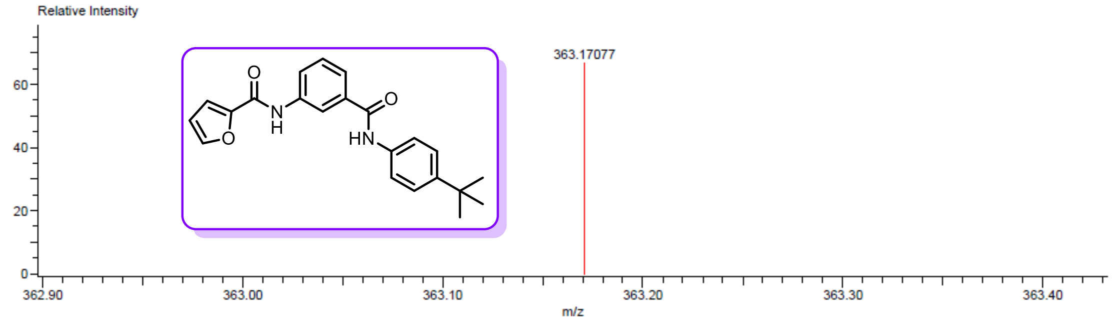

Mass	Intensity	Calc. Mass	Mass Difference (mmu)	Mass Difference (ppm)	Possible Formula	Unsaturation Number
363.17077	177896.59	363.17087	-0.10	-0.27	$^{12}C_{22}{}^{1}H_{23}{}^{14}N_2{}^{16}O_3$	12.5

Spectrum 64. HRMS of *N*-(3-((4-(*tert*-butyl)phenyl)carbamoyl)phenyl)furan-2-carboxamide (**6d**).

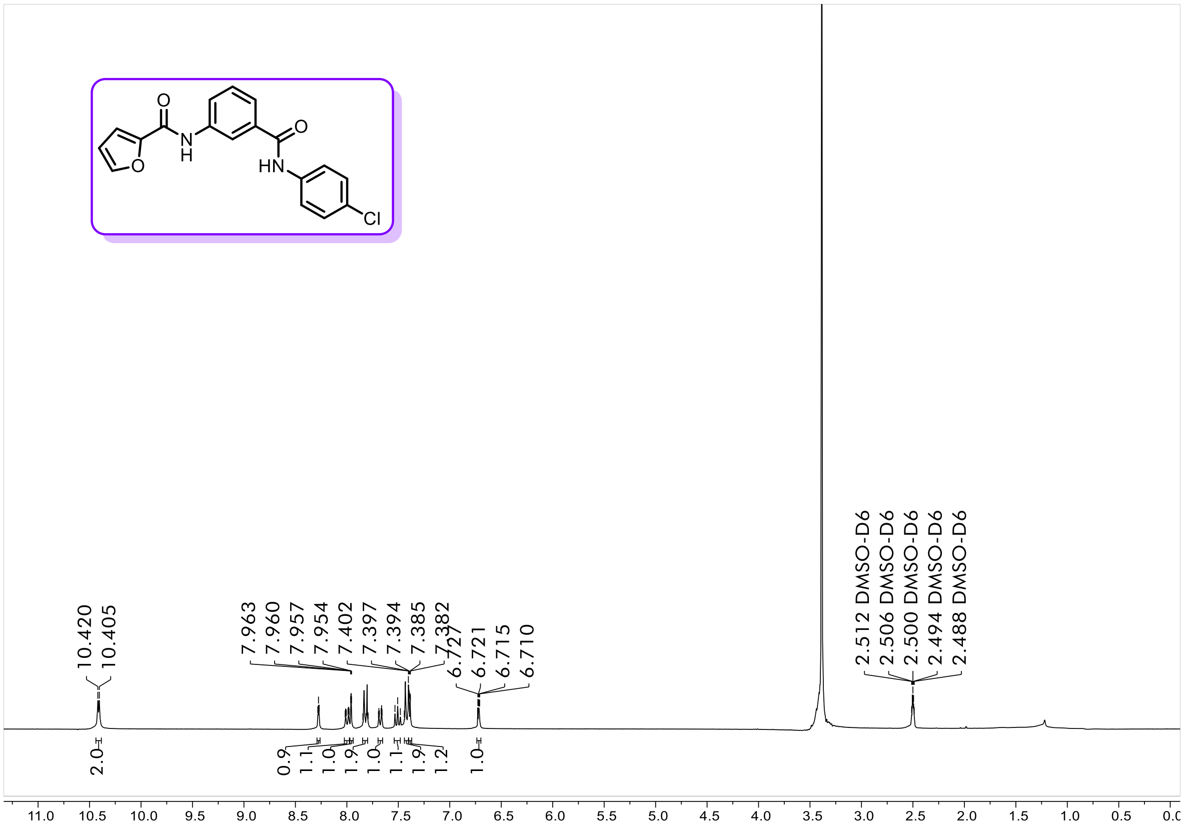

Spectrum 65. ^{1}H-NMR of *N*-(3-((4-chlorophenyl)carbamoyl)phenyl)furan-2-carboxamide (**6e**).

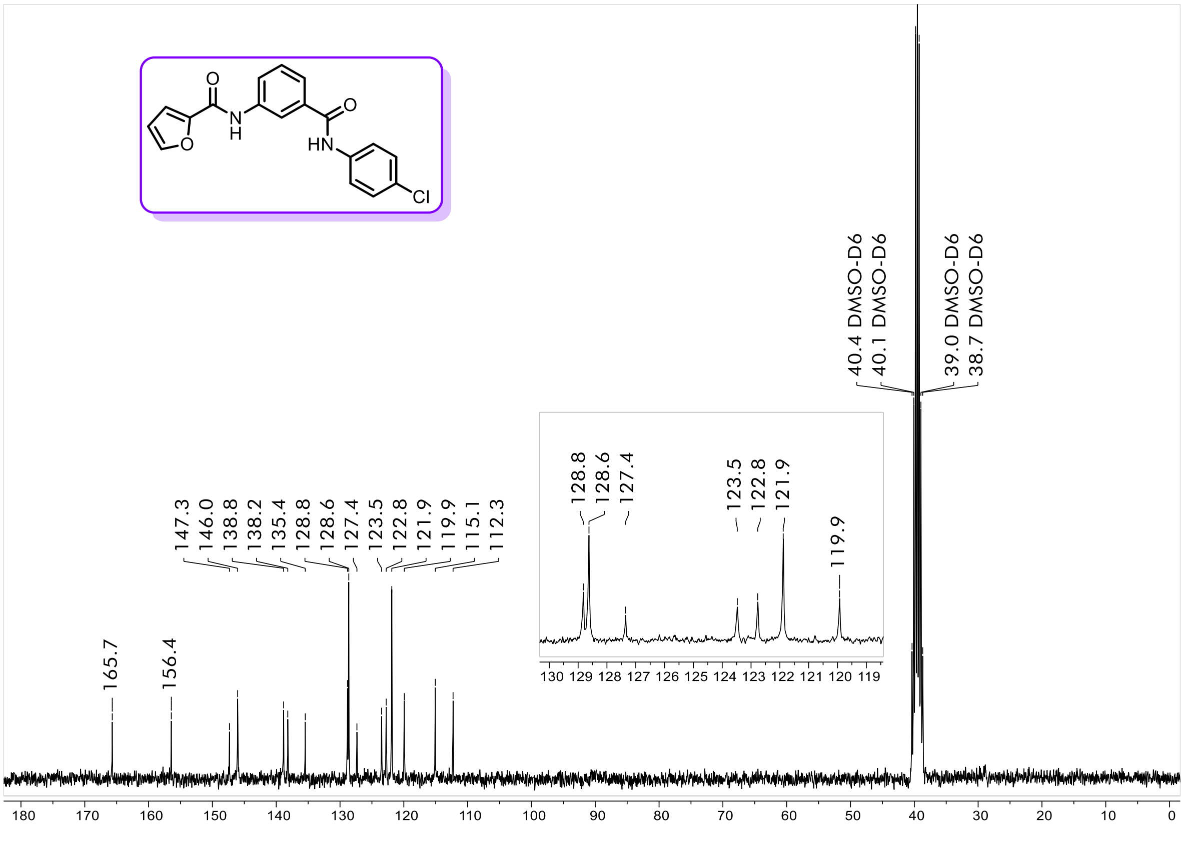

Spectrum 66. ^{13}C-NMR of *N*-(3-((4-chlorophenyl)carbamoyl)phenyl)furan-2-carboxamide (**6e**).

Description:
Ionization Mode:ESI+
History:Determine m/z[Peak Detect[Centroid,30,Area];Correct Base[];Smooth[5]];Correct Base[5.0%];Average(MS[...

Mass Calibration data:Cal_PEG_600
Created:11/24/2023 10:08:28 AM
Created by:AccuTOF

Charge number:1
Tolerance:4.00(mmu)
Unsaturation Number:-1.0 .. 70.0 (Fraction:Both)
Element:^{12}C:0 .. 18, ^{1}H:0 .. 60, ^{35}Cl:1 .. 1, ^{14}N:0 .. 2, ^{16}O:3 .. 3

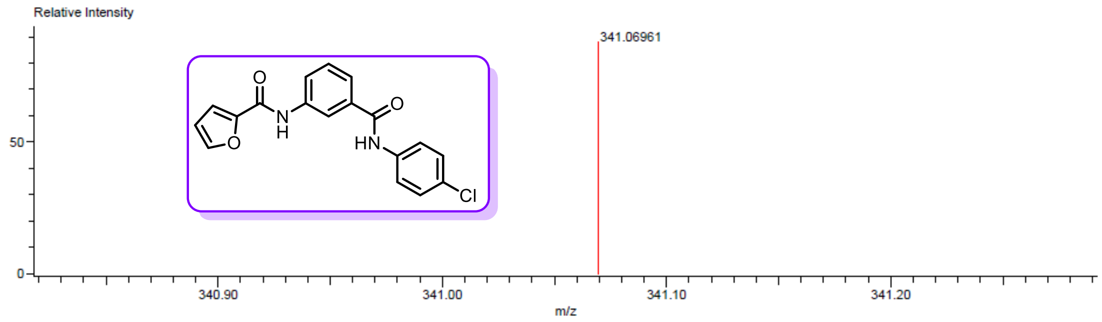

Mass	Intensity	Calc. Mass	Mass Difference (mmu)	Mass Difference (ppm)	Possible Formula	Unsaturation Number
341.06961	90840.77	341.06929	0.32	0.93	$^{12}C_{18}{}^{1}H_{14}{}^{35}Cl_{1}{}^{14}N_{2}{}^{16}O_{3}$	12.5

Spectrum 67 HRMS of *N*-(3-((4-chlorophenyl)carbamoyl)phenyl)furan-2-carboxamide (**6e**).

NMR SPECTRA OF TRIAZOLES 7a-e

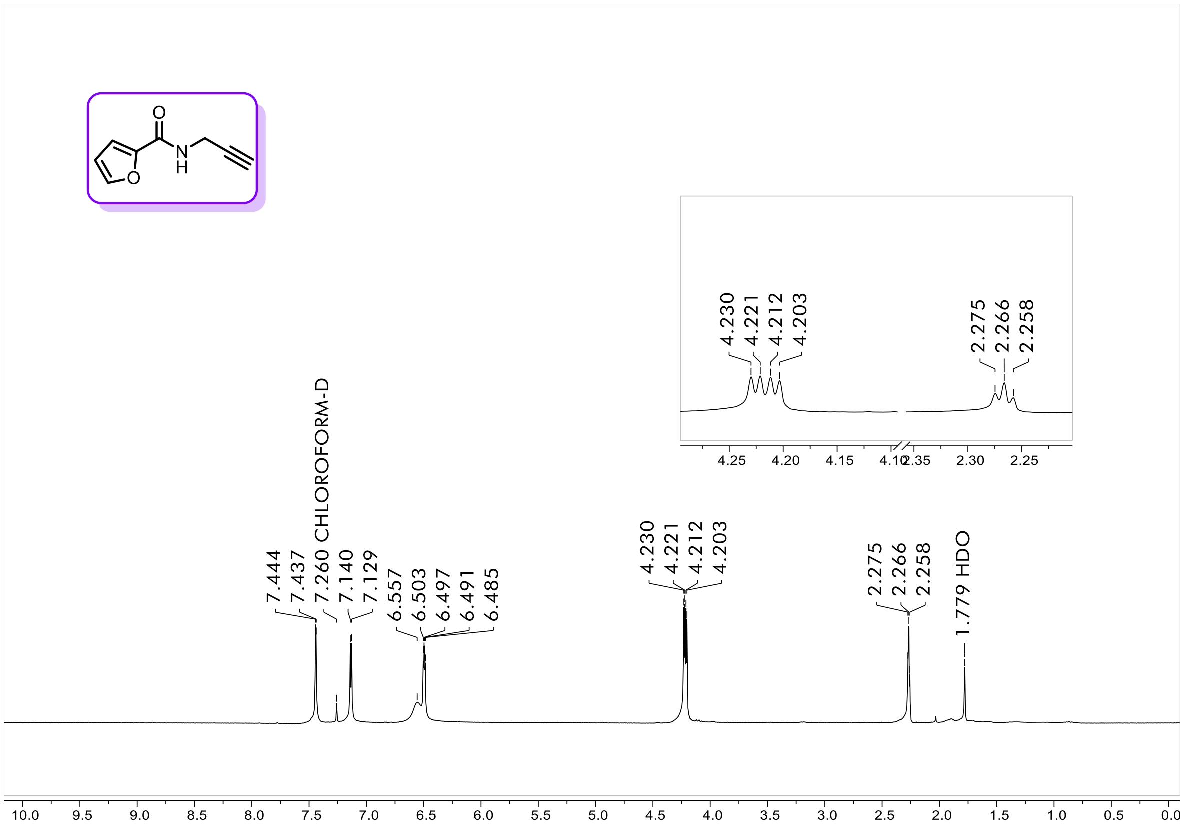

Spectrum 68 ^{1}H-NMR of *N*-(prop-2-yn-1-yl)furan-2-carboxamide (**15**).

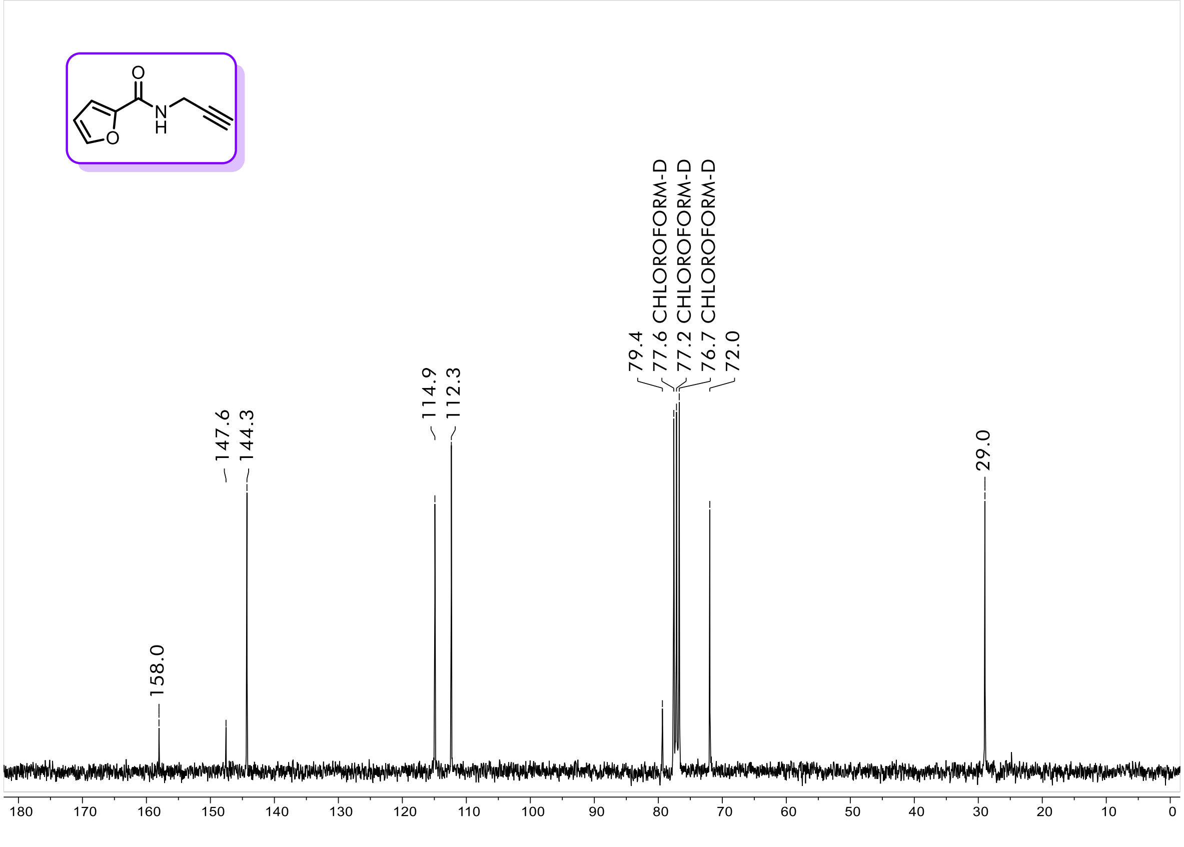

Spectrum 69 ^{13}C-NMR of *N*-(prop-2-yn-1-yl)furan-2-carboxamide (**15**).

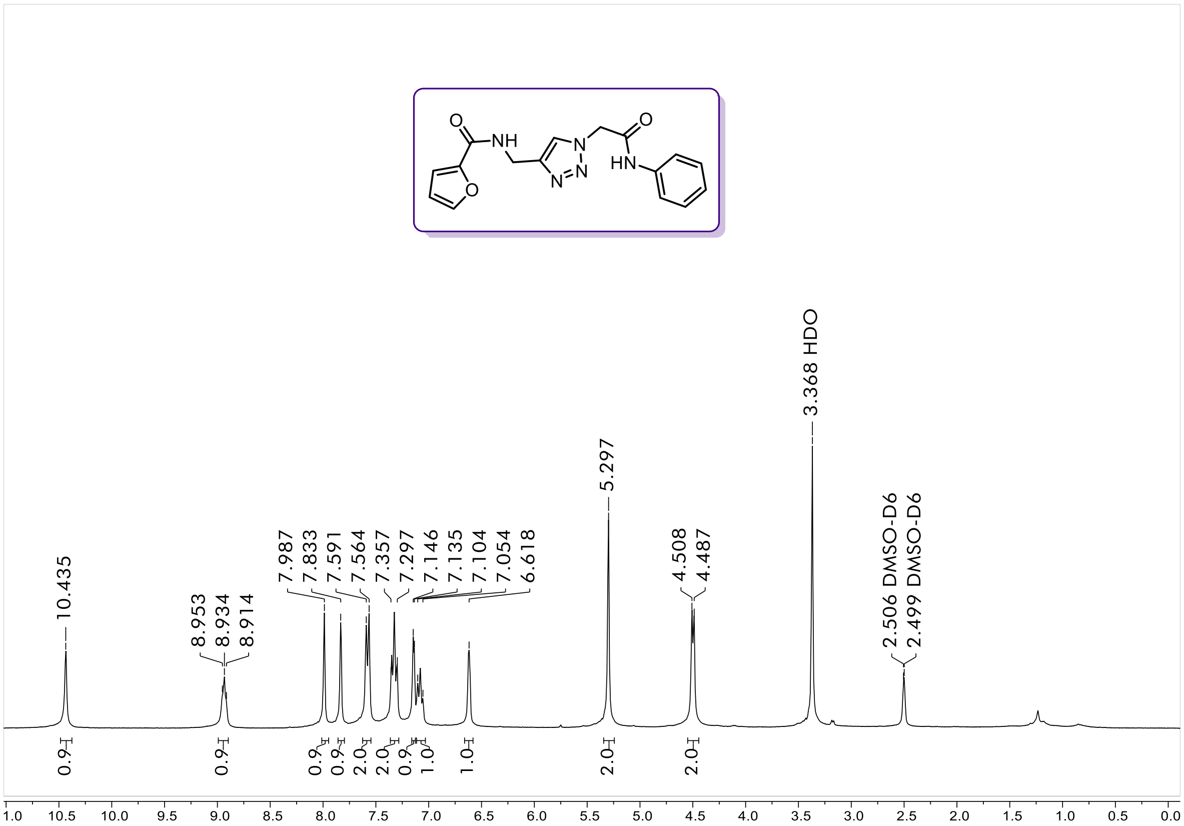

Spectrum 70. ^{1}H-NMR of *N*-((1-(2-oxo-2-(phenylamino)ethyl)-1*H*-1,2,3-triazol-4-yl)methyl)furan-2-carboxamide (**7a**).

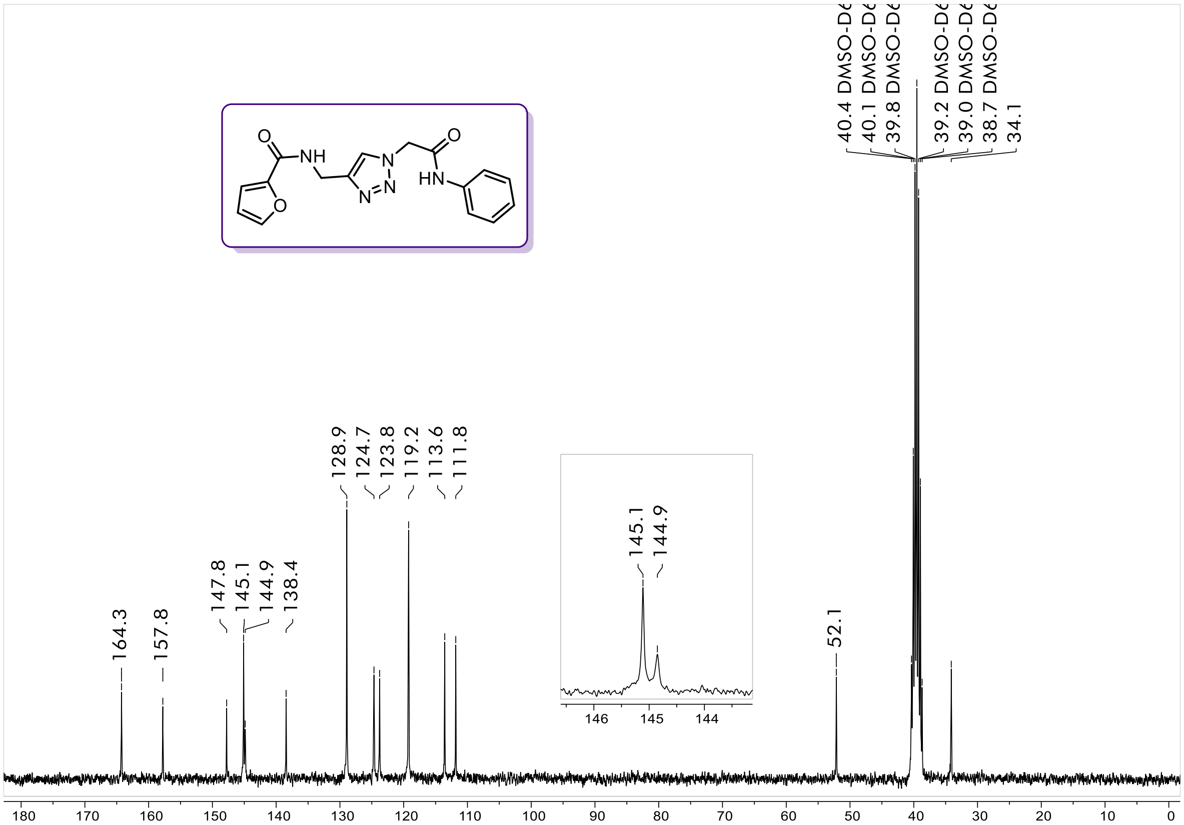

Spectrum 71. ^{13}C-NMR of *N*-((1-(2-oxo-2-(phenylamino)ethyl)-1*H*-1,2,3-triazol-4-yl)methyl)furan-2-carboxamide (**7a**).

Description:
Ionization Mode:ESI+
History:Determine m/z[Peak Detect[Centroid,30,Area];Correct Base[];Smooth[5]];Correct Base[5.0%];Average(MS[...

Mass Calibration data:Cal_PEG_600
Created:8/3/2023 2:18:22 PM
Created by:AccuTOF

Charge number:1
Element:^{12}C:0 .. 16, ^{1}H:0 .. 50, ^{14}N:3 .. 5, ^{16}O:3 .. 5

Tolerance:500.00(ppm), 5.00 .. 15.00(mmu)

Unsaturation Number:-1.0 .. 54.0 (Fraction:Both)

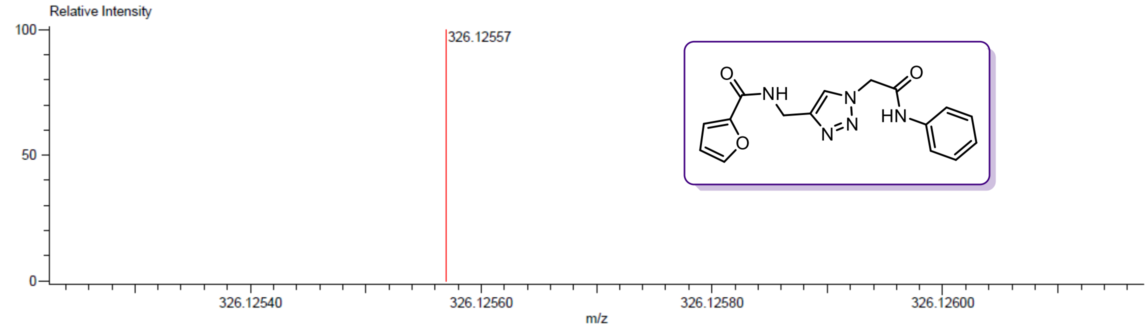

Mass	Intensity	Calc. Mass	Mass Difference (mmu)	Mass Difference (ppm)	Possible Formula	Unsaturation Number
326.12557	1604813.43	326.12531	0.26	0.79	$^{12}C_{16}{}^{1}H_{16}{}^{14}N_{5}{}^{16}O_{3}$	11.5

Spectrum 72. HRMS of *N*-((1-(2-oxo-2-(phenylamino)ethyl)-1*H*-1,2,3-triazol-4-yl)methyl)furan-2-carboxamide (**7a**).

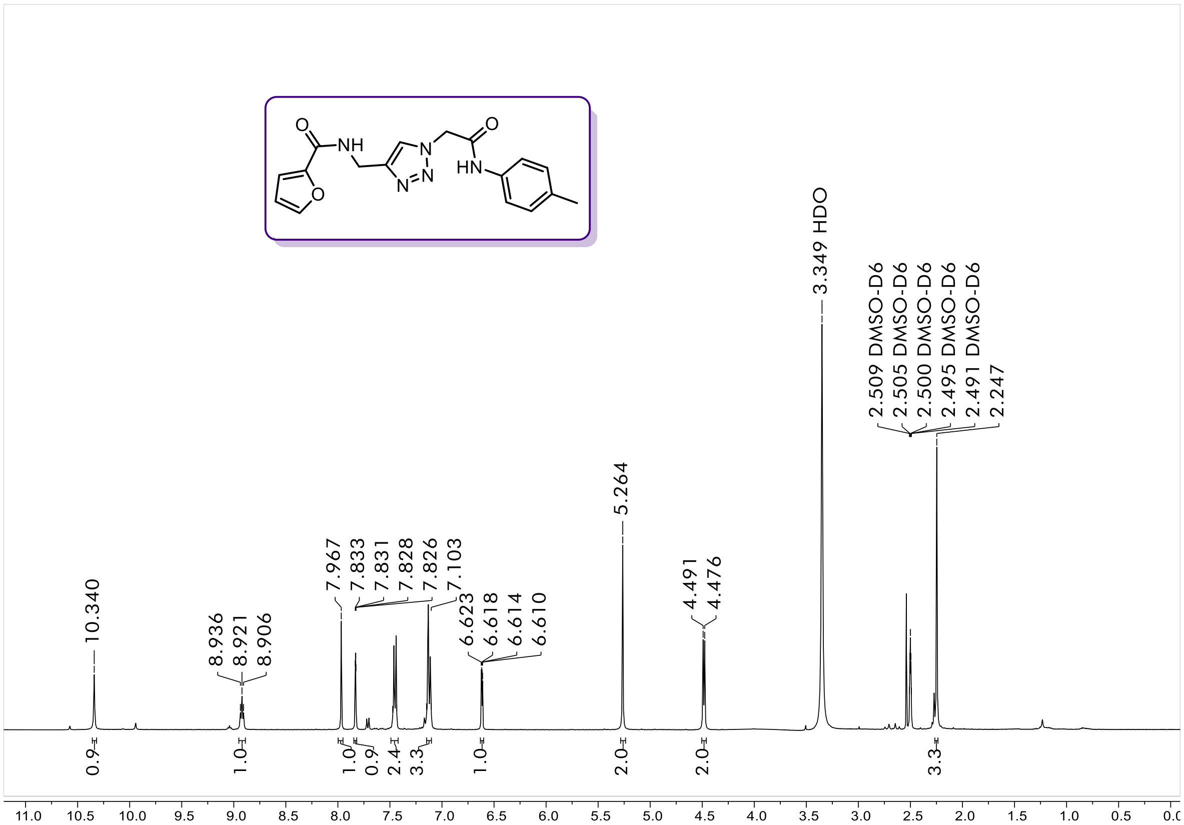

Spectrum 73. ^{1}H-NMR of *N*-((1-(2-oxo-2-(*p*-tolylamino)ethyl)-1*H*-1,2,3-triazol-4-yl)methyl)furan-2-carboxamide (**7b**).

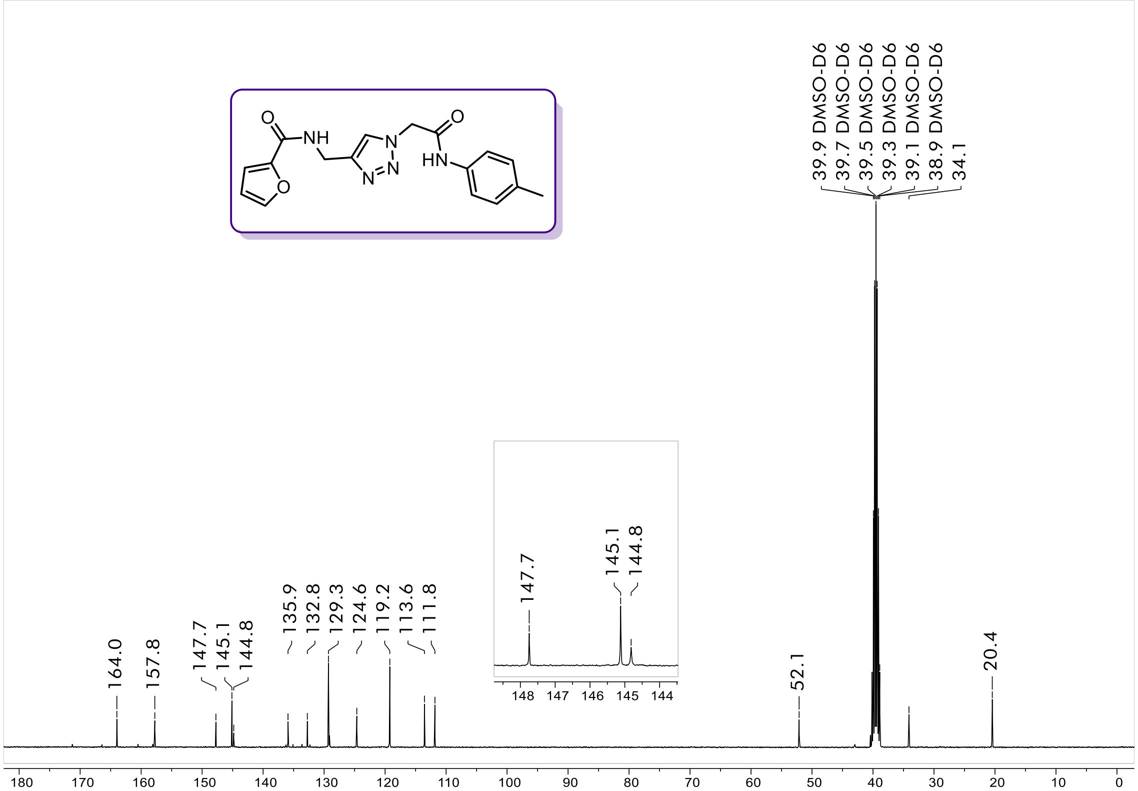

Spectrum 74. ^{13}C-NMR of *N*-((1-(2-oxo-2-(*p*-tolylamino)ethyl)-1*H*-1,2,3-triazol-4-yl)methyl)furan-2-carboxamide (**7b**).

Description:
Ionization Mode:ESI+
History:Determine m/z[Peak Detect[Centroid,30,Area];Correct Base[];Smooth[5]];Correct Base[5.0%];Average(MS[...

Mass Calibration data:Cal_PEG_600
Created:8/10/2023 1:24:24 PM
Created by:AccuTOF

Charge number:1
Element:^{12}C:0 .. 17, ^{1}H:0 .. 18, ^{14}N:5 .. 5, ^{16}O:3 .. 3

Tolerance:4.00(mmu)

Unsaturation Number:-1.0 .. 30.0 (Fraction:.5)

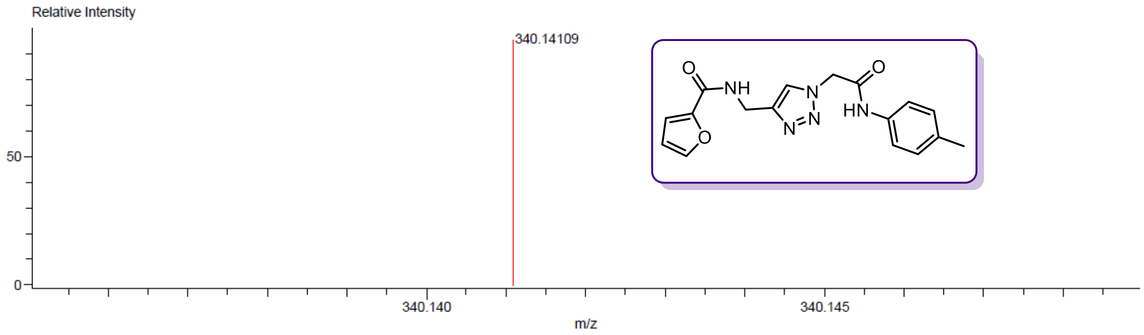

Mass	Intensity	Calc. Mass	Mass Difference (mmu)	Mass Difference (ppm)	Possible Formula	Unsaturation Number
340.14109	84712.29	340.14096	0.13	0.37	$^{12}C_{17}{}^{1}H_{18}{}^{14}N_5{}^{16}O_3$	11.5

Spectrum 75. HRMS of *N*-((1-(2-oxo-2-(*p*-tolylamino)ethyl)-1*H*-1,2,3-triazol-4-yl)methyl)furan-2-carboxamide (**7b**).

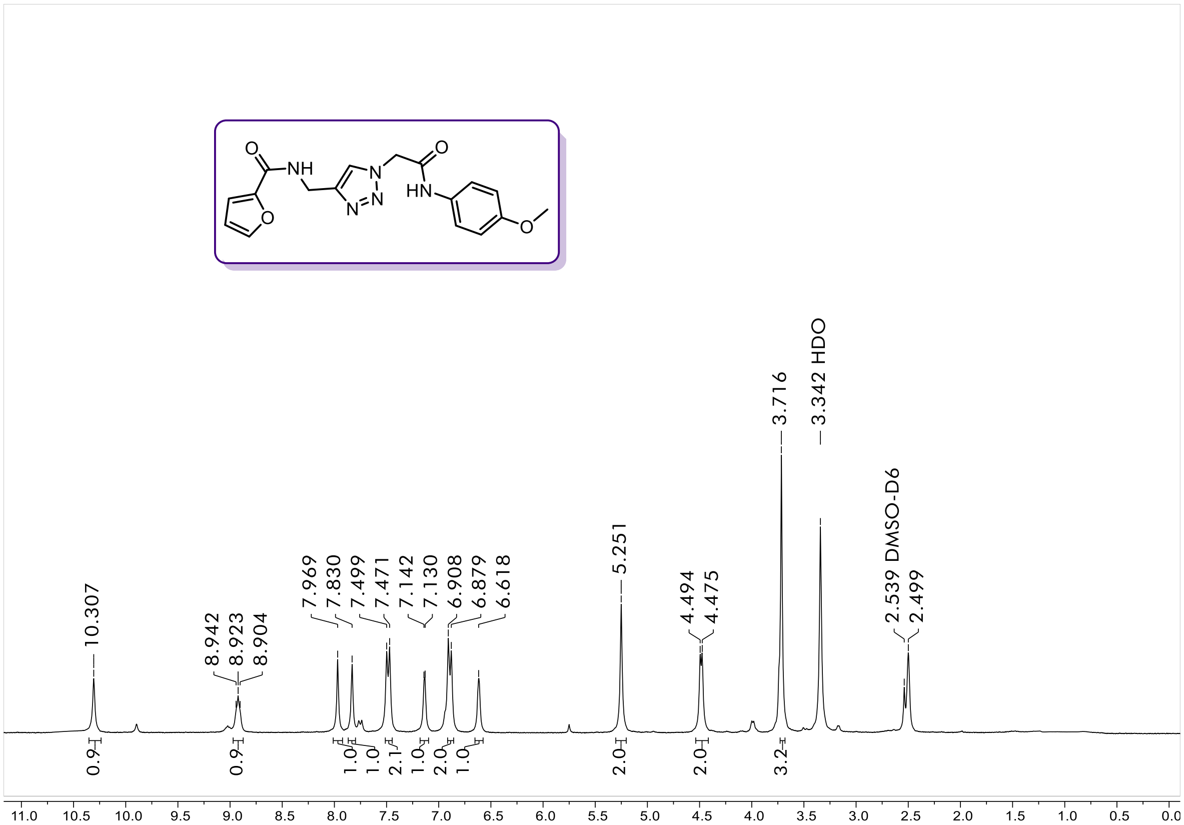

Spectrum 76. ^{1}H-NMR of *N*-((1-(2-((4-methoxyphenyl)amino)-2-oxoethyl)-1*H*-1,2,3-triazol-4-yl)methyl)furan-2-carboxamide (**7c**).

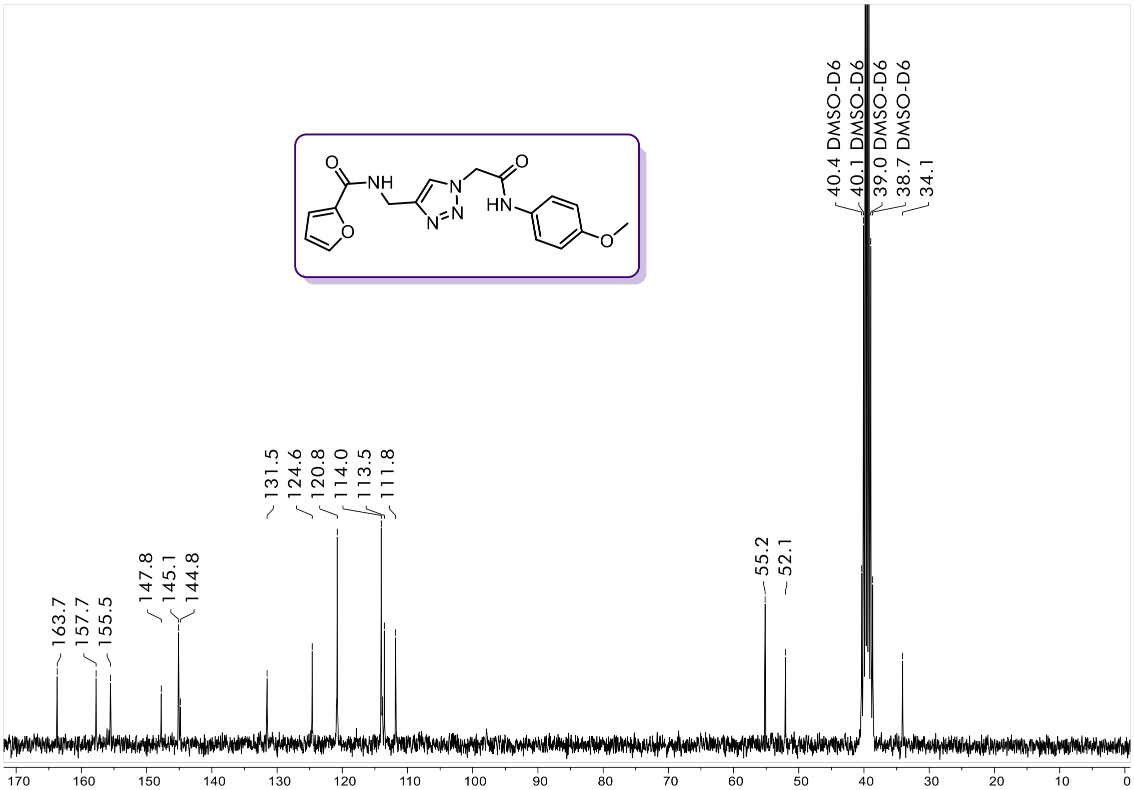

Spectrum 77. ^{13}C-NMR of *N*-((1-(2-((4-methoxyphenyl)amino)-2-oxoethyl)-1*H*-1,2,3-triazol-4-yl)methyl)furan-2-carboxamide (**7c**).

Description:
Ionization Mode:ESI+
History:Determine m/z[Peak Detect[Centroid,30,Area];Correct Base[];Smooth[5]];Correct Base[5.0%];Average(MS[...

Mass Calibration data:Cal_PEG_600
Created:8/7/2023 4:17:32 PM
Created by:AccuTOF

Charge number:1
Element:^{12}C:0 .. 17, ^{1}H:0 .. 18, ^{14}N:0 .. 5, ^{16}O:0 .. 4

Tolerance:5.00(ppm), 5.00 .. 15.00(mmu)

Unsaturation Number:-1.0 .. 30.0 (Fraction:Both)

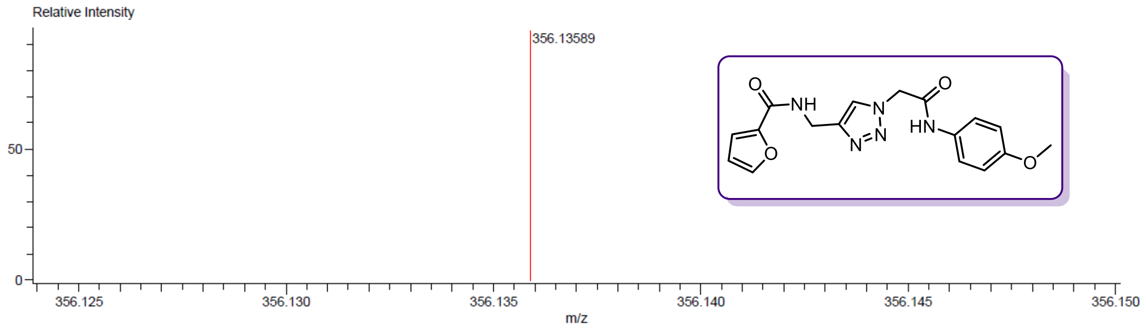

Mass	Intensity	Calc. Mass	Mass Difference (mmu)	Mass Difference (ppm)	Possible Formula	Unsaturation Number
356.13589	111338.03	356.13588	0.02	0.04	$^{12}C_{17}{}^{1}H_{18}{}^{14}N_{5}{}^{16}O_{4}$	11.5

Spectrum 78. HRMS of *N*-((1-(2-((4-methoxyphenyl)amino)-2-oxoethyl)-1*H*-1,2,3-triazol-4-yl)methyl)furan-2-carboxamide (**7c**).

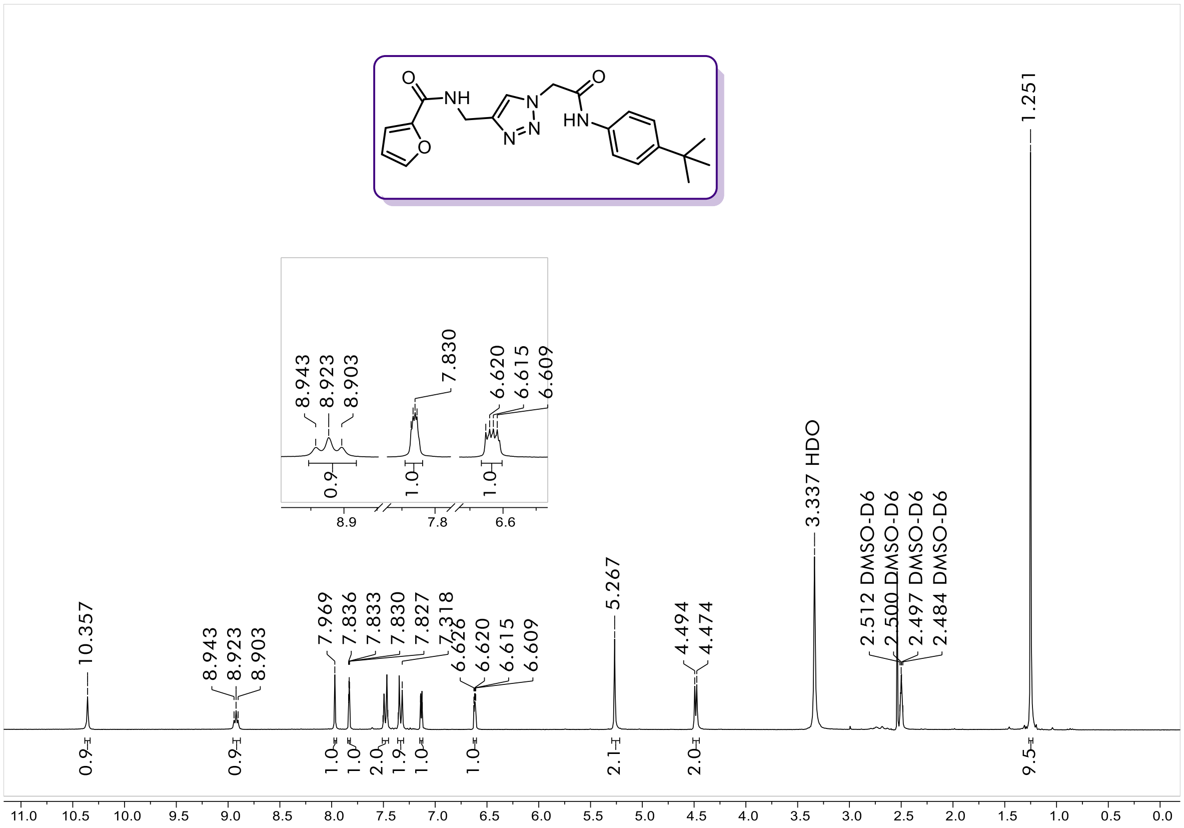

Spectrum 79. ^{1}H-NMR *N*-((1-(2-((4-(*tert*-butyl)phenyl)amino)-2-oxoethyl)-1*H*-1,2,3-triazol-4-yl)methyl)furan-2-carboxamide (**7d**).

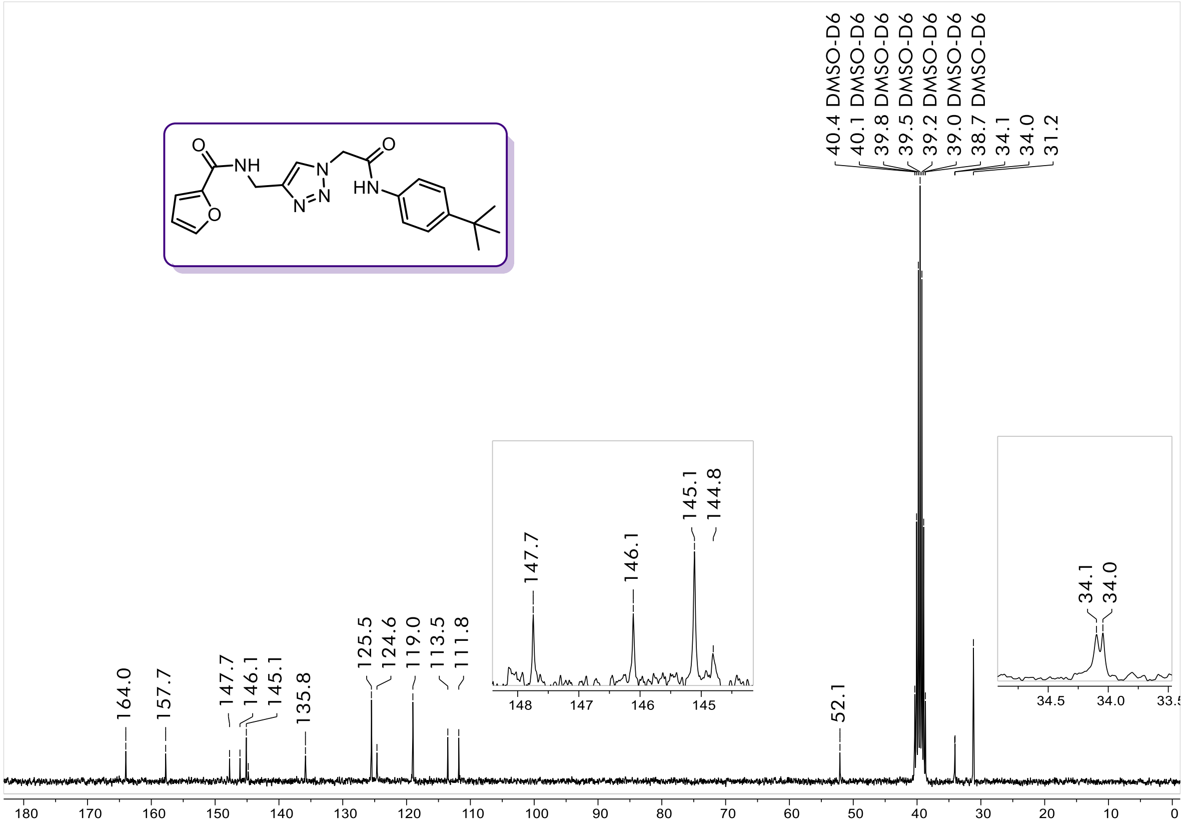

Spectrum 80. ^{13}C-NMR *N*-((1-(2-((4-(*tert*-butyl)phenyl)amino)-2-oxoethyl)-1*H*-1,2,3-triazol-4-yl)methyl)furan-2-carboxamide (**7d**).

Description:
Ionization Mode:ESI+
History:Determine m/z[Peak Detect[Centroid,30,Area];Correct Base[];Smooth[5]];Correct Base[5.0%];Average(MS[...

Mass Calibration data:Cal_PEG_600
Created:8/16/2023 11:54:18 AM
Created by:AccuTOF

Charge number:1
Tolerance:5.00(ppm), 5.00 .. 15.00(mmu)
Unsaturation Number:-1.0 .. 17.0 (Fraction:Both)
Element:^{12}C:0 .. 20, ^{1}H:0 .. 35, ^{127}I:0 .. 0, ^{14}N:0 .. 5, ^{16}O:1 .. 3

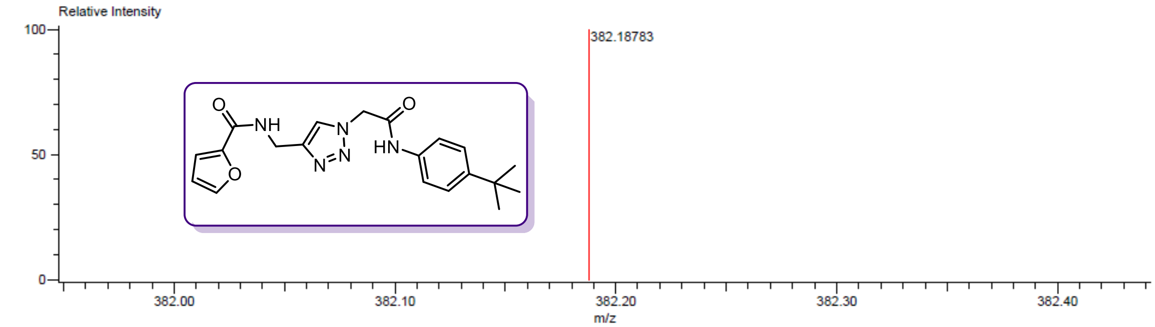

Mass	Intensity	Calc. Mass	Mass Difference (mmu)	Mass Difference (ppm)	Possible Formula	Unsaturation Number
382.18783	3435542.05	382.18791	-0.09	-0.23	$^{12}C_{20}{}^{1}H_{24}{}^{14}N_{5}{}^{16}O_{3}$	11.5

Spectrum 81. HRMS *N*-((1-(2-((4-(*tert*-butyl)phenyl)amino)-2-oxoethyl)-1*H*-1,2,3-triazol-4-yl)methyl)furan-2-carboxamide (**7d**).

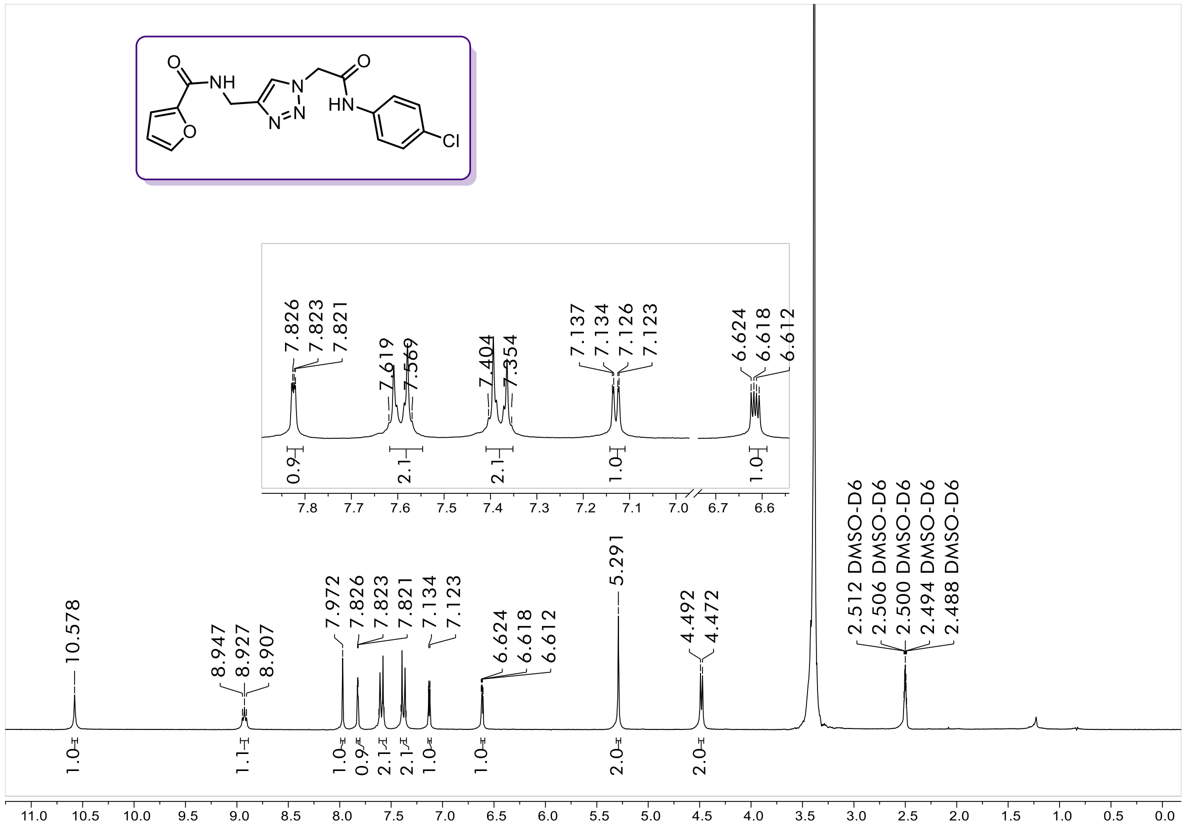

Spectrum 82. ^{1}H-NMR of *N*-((1-(2-((4-chlorophenyl)amino)-2-oxoethyl)-1*H*-1,2,3-triazol-4-yl)methyl)furan-2-carboxamide (**7e**).

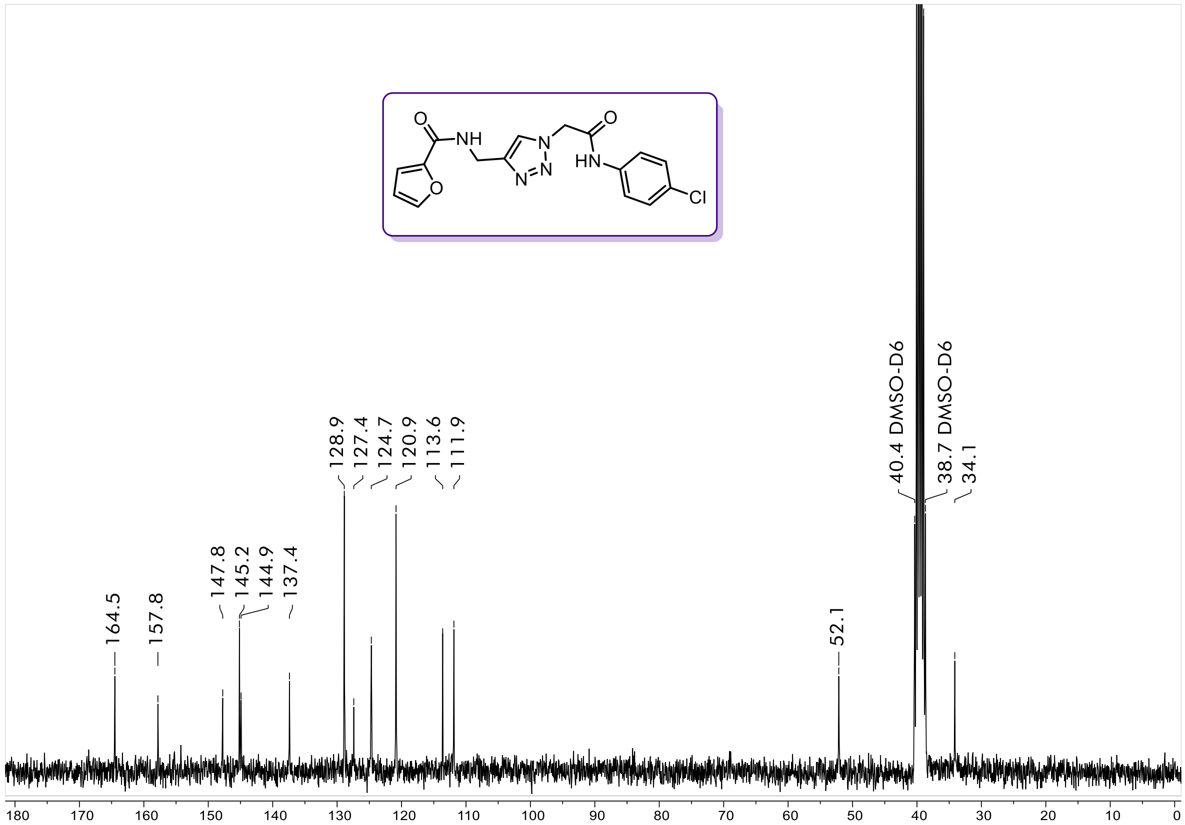

Spectrum 83. ^{13}C-NMR of *N*-((1-(2-((4-chlorophenyl)amino)-2-oxoethyl)-1*H*-1,2,3-triazol-4-yl)methyl)furan-2-carboxamide (**7e**).

Ionization Mode:ESI+
History:Determine m/z[Peak Detect[Centroid,30,Area];Correct Base[];Smooth[5]];Correct Base[5.0%];Average(MS[...

Created:8/3/2023 2:22:44 PM
Created by:AccuTOF

Charge number:1
Element:^{12}C:0 .. 16, ^{1}H:0 .. 50, ^{35}Cl:1 .. 1, ^{14}N:3 .. 5, ^{16}O:3 .. 5

Tolerance:500.00(ppm), 5.00 .. 15.00(mmu)

Unsaturation Number:-1.0 .. 54.0 (Fraction:Both)

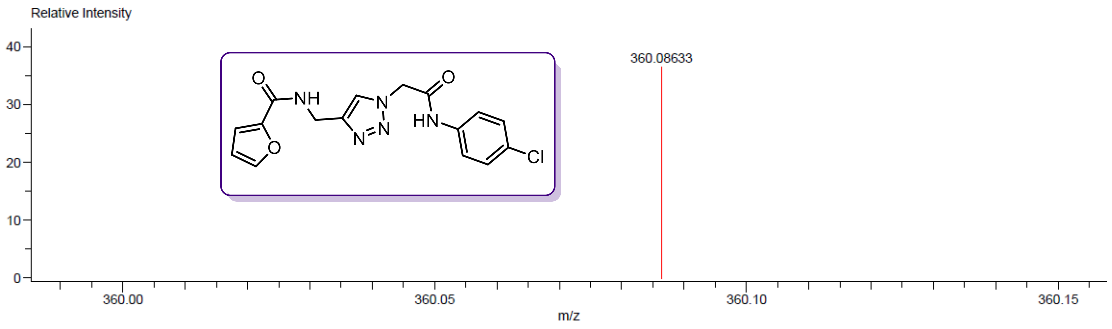

Mass	Intensity	Calc. Mass	Mass Difference (mmu)	Mass Difference (ppm)	Possible Formula	Unsaturation Number
360.08633	352361.07	360.08634	-0.01	-0.04	$^{12}C_{16}{}^{1}H_{15}{}^{35}Cl_{1}{}^{14}N_{5}{}^{16}O_{3}$	11.5

Spectrum 83. HRMS of *N*-((1-(2-((4-chlorophenyl)amino)-2-oxoethyl)-1*H*-1,2,3-triazol-4-yl)methyl)furan-2-carboxamide (**7e**).

Figures S1-S4

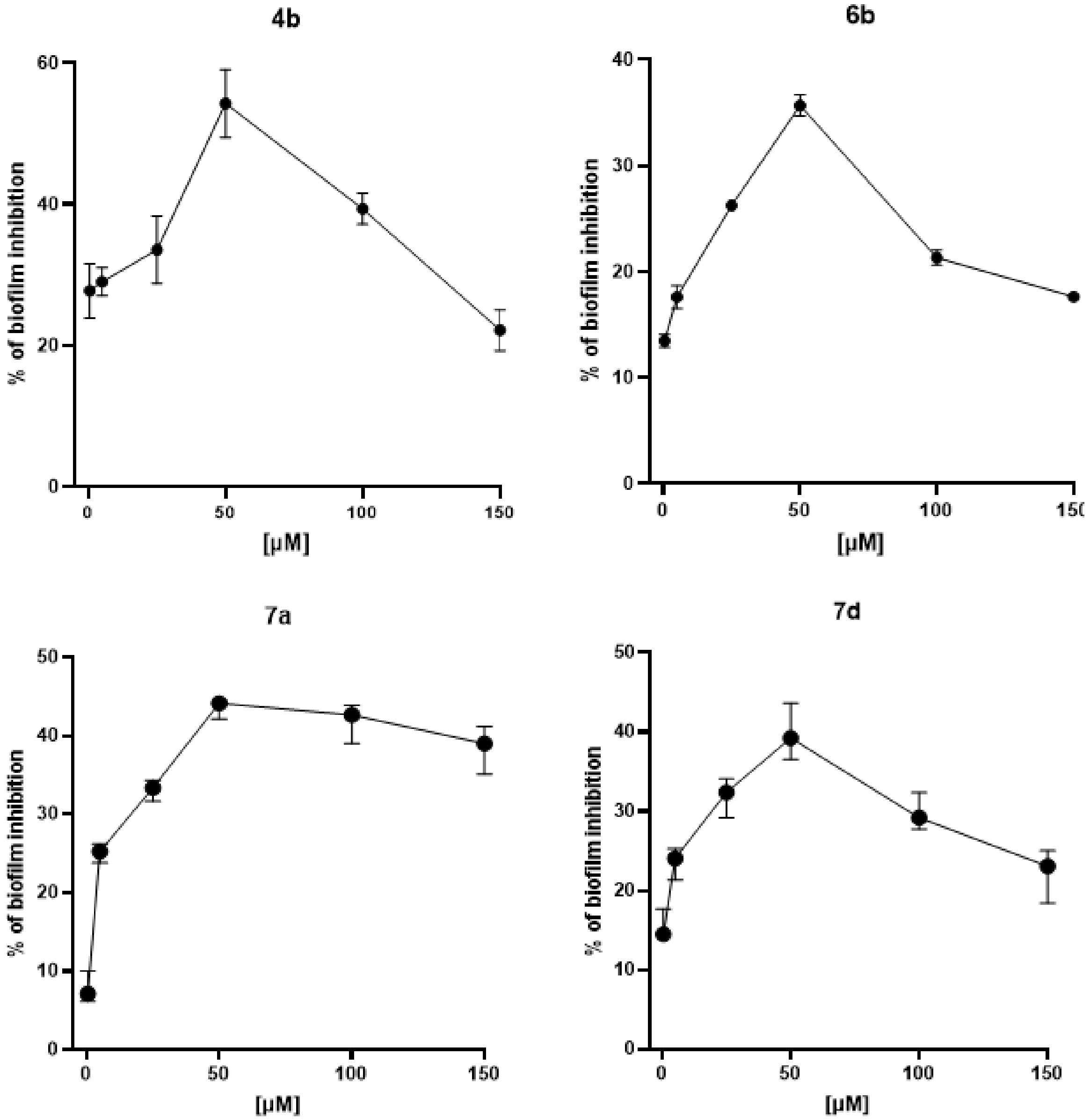

Figure S1. Concentration-response curve (antibiofilm capacity) for **4b, 6b, 7a** and **7d**. After 50 μM, the carboxamides precipitated. Data represents the mean ± S.E.M. from three independent experiments.

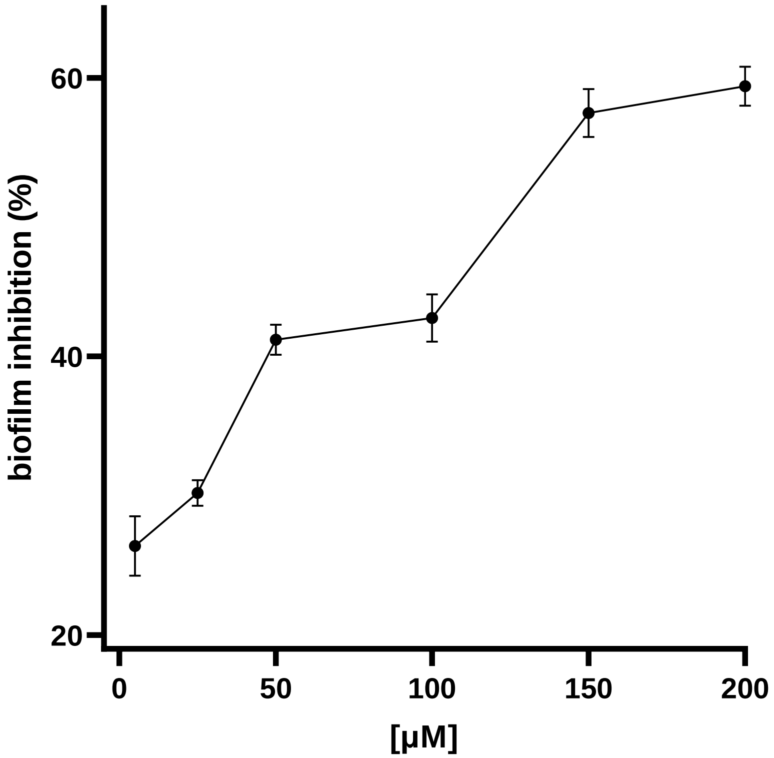

Figure S2. IC_{50} calculation of furanone **2** (C30). Data represents the mean ± S.E.M. from three independent experiments.

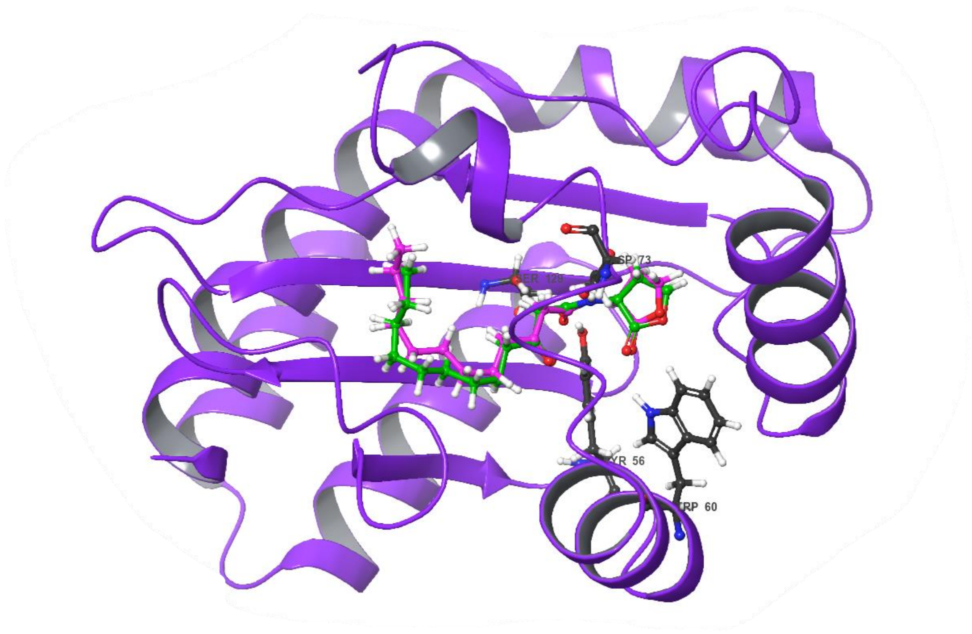

Figure S3. Docking validation. Co-crystallized (green) and docked (magenta) autoinducer C12 have a similar binding pose (orientation and conformation) and share the same hydrogen bonds with Tyr56, Trp60, Asp73, and Ser129.

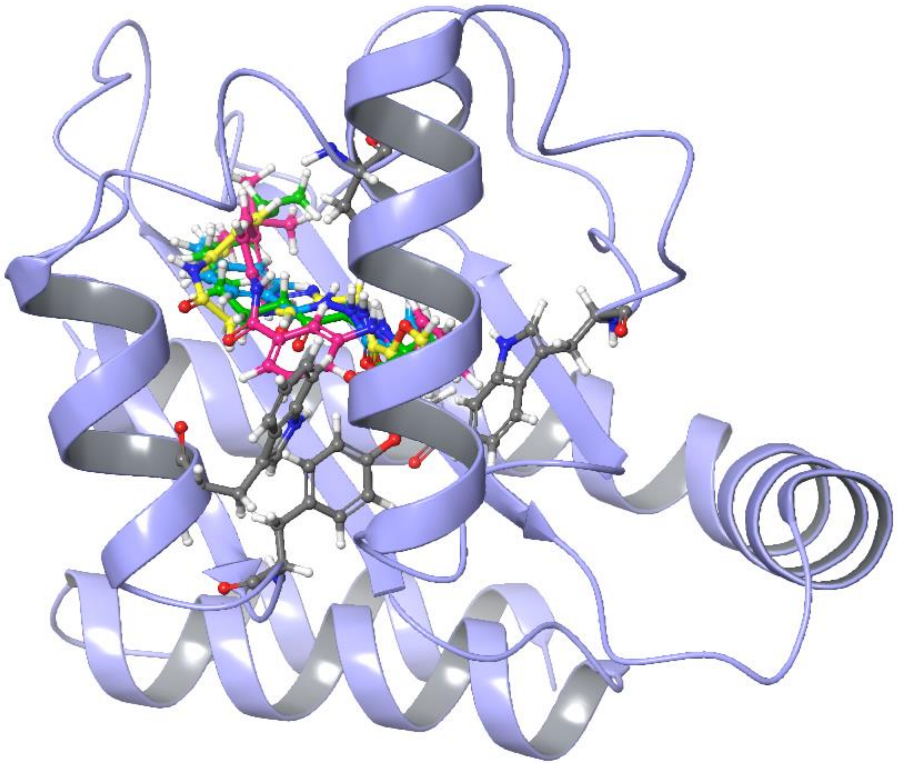

Figure S4. Binding poses of selected carboxamides inside LasR after docking study. Green: autoinducer (C12); blue: **4b**; magenta: 7d; Yellow: **7e**.
